# Heart Attack Education and EMS Response in High-Risk, Low EMS Usage Areas: A Stepped-Wedge Cluster-Randomized Trial

**DOI:** 10.1001/jamanetworkopen.2026.8823

**Published:** 2026-04-27

**Authors:** Janet E. Bray, Ziad Nehme, Judith C. Finn, Jessica Kasza, Janelle Woods, Robyn A. Clark, Dion Stub, Dominique A. Cadilhac, Joosup Kim, Ben J. Smith, Susie Cartledge, Alison Beauchamp, Rhys Bowden, Natasha Dodge, Elizabeth Flemming-Judge, Clara Chow, Nicholas Cox, William van Gaal, Voltaire Nadurata, Peter Cameron

**Affiliations:** 1 School of Public Health and Preventive Medicine, Monash University, Victoria, Australia; 2 Prehospital, Resuscitation & Emergency Care Research Unit, Curtin University, Perth, Australia; 3 Ambulance Victoria, Victoria, Australia; 4 Department of Paramedicine, Monash University, Victoria, Australia; 5 National Heart Foundation of Australia, Victoria, Australia; 6 Caring Futures Institute, Flinders University, South Australia, Australia; 7 Alfred Health, Victoria, Australia; 8 Stroke and Ageing Research, Department of Medicine, School of Clinical Sciences at Monash Health, Monash University, Victoria, Australia; 9 Stroke and Critical Care Research Group, Florey Institute of Neuroscience and Mental Health, University of Melbourne, Victoria, Australia; 10 Prevention Research Collaboration, University of Sydney, New South Wales, Australia; 11 School of Rural Health, Monash University, Victoria, Australia; 12 University of Sydney, New South Wales, Australia; 13 Westmead Hospital, New South Wales, Australia; 14 Western Health, Victoria, Australia; 15 Department of Medicine, University of Melbourne, Victoria, Australia; 16 Northern Health, Victoria, Australia; 17 Bendigo Health, Victoria, Australia

## Abstract

**Importance:**

Patient delays in recognizing and acting on acute coronary syndrome (ACS) symptoms worsen outcomes.

**Objective:**

To evaluate the effectiveness of a heart attack education intervention (Heart Matters) aiming to improve recognition and response to ACS symptoms in communities with elevated cardiovascular risk and low emergency medical service (EMS) use.

**Design, Setting, and Participants:**

This stepped-wedge cluster randomized clinical trial was conducted in 8 high-risk communities (local government areas; 4 metropolitan and 4 rural with a combined population of approximately 792 000 adult residents) in Victoria, Australia (December 2021 to March 2023 with follow-up to March 2024), with clusters crossing to the intervention every 4 months. Data were obtained from administrative datasets and registries (analysis complete June 2025).

**Intervention:**

A multicomponent community education program including 490 community sessions (approximately 10 088 residents), more than 174 110 households mailouts, more than 50 000 resource handouts, opportunistic media, and a geotargeted social-media campaign (reach of approximately 350 000 residents). The control period did not include any educational campaign.

**Main Outcome and Measures:**

The primary outcome was the proportion of patients with ACS transported to hospitals by EMS, as recorded in the Victorian Emergency Minimum Dataset. Outcomes were analyzed at the patient level using mixed-effects regression models, reporting risk differences (RDs) and odds ratios (ORs). A program evaluation was also conducted to assess implementation and inform potential replication and scale-up.

**Results:**

Among 1775 patients with ACS (865 intervention and 910 control; 924 [52.1%] aged ≥65 years; 1193 male [67.2%]), EMS use was unexpectedly high during the initial control period, coinciding with a COVID-19 wave. EMS transport occurred in 624 of 910 patients with ACS (68.6%) in the control period and 548 of 865 patients (63.4%) in the intervention period (adjusted RD, –8.98%; 95% CI, –17.50% to –0.46%; *P* = .04; adjusted OR, 0.67; 95% CI, 0.45 to 1.01; *P* = .05). Reductions were more pronounced in metropolitan areas (RD –10.73%; 95% CI –20.43% to –1.03%) and during a severe flooding event (RD –13.50%; 95% CI –26.52% to –0.47%). Early treatment-seeking was also lower, although estimates were imprecise. The program evaluation identified COVID-19 pandemic and seasonal impacts, as well as community concerns regarding EMS costs, demand, and wait times.

**Conclusions and Relevance:**

In this community ACS education trial conducted in high-risk regions, the intervention did not improve EMS use or prehospital care-seeking. External contextual factors, including the COVID-19 pandemic and natural disasters, appeared to influence patient behavior and may have attenuated intervention effects, highlighting the complexity of achieving behavioral change through community education alone.

**Trial Registration:**

ClinicalTrials.gov Identifier: NCT04995900

## Key Points

**Question:**

Does community-based heart attack education in regions with high cardiovascular risk and low emergency medical services (EMS) use improve patients’ response to acute coronary syndrome (ACS)?

**Findings:**

In this stepped-wedge, cluster-randomized trial including 1775 patients with ACS, there was a 9% reduction in EMS use during the intervention. Coordinators reported that the COVID-19 pandemic and seasonal weather effects reduced event attendance, and that the community raised concerns about costs, emergency demand, and wait times.

**Meaning:**

These findings suggest that community education interventions are complex and can be influenced by external factors that may negate the intervention’s impact.

## Teaser

This stepped-wedge cluster-randomized trial evaluates the effectiveness of a heart attack education intervention to improve recognition and response to acute coronary syndrome symptoms in communities with elevated cardiovascular risk and low emergency medical service (EMS) use in Australia.

## Introduction

Acute coronary syndrome (ACS) remains a leading cause of death and disability worldwide.^1^ Prompt treatment is critical,^2^ yet patient delay is common^3^ and results in prolonged ischemic times and worse clinical outcomes.^4,5,6^ Many patients with ACS delay seeking care or do not call emergency medical services (EMS),^6,7^ limiting opportunities for early diagnosis, triage, and delivery of guideline-directed therapies.^8,9,10,11^ Barriers to EMS use include poor symptom recognition, underestimation of severity, cost concerns, and misconceptions about EMS use.^8,12^

Strategies to reduce delays and increase EMS use show mixed effectiveness, and few contemporary community trials exist.^13^ Lessons from the largest trial to date, the REACT trial, highlight the importance of targeting high-risk regions with low awareness and poor treatment-seeking behavior rather than the general population.^14,15^ To address these gaps, the Heart Matters trial^16^ evaluated whether a multifaceted community education campaign in high-risk local government areas (LGAs) improved ACS knowledge within these communities, increased awareness of personal risk, and prompted appropriate treatment-seeking behaviors when experiencing ACS.

## Methods

### Trial Design

This community-based, pragmatic, stepped-wedge, cluster-randomized clinical trial was conducted in 8 high-risk LGAs (clusters) in Victoria, Australia, between December 2021 and March 2023, with continued follow-up of the primary outcome to March 2024.^16^ The analysis, using administrative and registry data, was completed in June 2025. A cluster-randomized design was chosen because the intervention was implemented at the population level, with the stepped-wedge design selected for practicality and resource constraints.

High-risk LGAs were identified by examining previously collected data across the state, including ACS presentations to hospitals,^17^ out-of-hospital cardiac arrest incidence,^18^ and surveys on ACS knowledge^19^ and risk factors.^20^ Eight areas were selected from a list of 16 high-risk LGAs based on sample size and geographic separation to prevent contamination. The 8 selected clusters randomly transitioned from control to intervention, every 4 months, with a 2-month transition period. The trial protocol (Supplement 1) was approved by the Monash University Human Ethics Committee under a waiver of informed consent due to the use of deifentified data. The trial is reported in accordance with the Consolidated Standards of Reporting Trials Extension (CONSORT Extension) for stepped-wedge cluster-randomized trials.^21^

There were some changes from the original trial protocol (Supplement 1). We initially planned to interview patients with ACS during their hospital stay to determine their exposure to the intervention and its impact on their choice of mode of transport to the hospital and prehospital delay times. However, hospital-based research was not possible at the study’s commencement due to the COVID-19 pandemic. Prehospital delay times for residents were instead collected via a registry for ACS patients who received revascularization.

### Setting

An LGA is a defined administrative region governed by a local council, responsible for providing community services, infrastructure, and regulatory functions within its boundaries. All adult residents of the selected LGAs were eligible to receive the intervention. At the time of the study, the 8 areas represented approximately 17% of the state’s population (approximately 792 000 adults) (eTable 1 in Supplement 2). Four LGAs were in metropolitan Melbourne, including 1 LGA in the inner city and 3 LGAs on the city outskirts, and 4 LGAs were in rural locations.

### Trial Intervention and Implementation Strategy

The intervention and its implementation are summarized in Supplement 1 in adherence with the Template for Intervention Description and Replication Checklist.^22^ In brief, the intervention was a community-based educational intervention designed to improve awareness of personal risk, recognition of ACS symptoms, and highlight the importance of timely EMS use and presentation to hospital. Given the size of the regions and to aid targeting the intervention, the same data that identified LGAs were used to identify high-risk demographics and priority postcodes within each cluster.

Key elements of the intervention included in-person and online education sessions delivered by trained Heart Matters coordinators; specifically developed videos featuring a celebrity paramedic promoting EMS use for chest pain, as well as patient videos featuring people of various demographic backgrounds recalling their symptoms and actions; distribution of the Heart Foundation Heart Attack Action Plans, including a mailout to households in the highest risk priority postcodes; opportunistic local media coverage (eg, local radio or newspapers) and a 2-month LGA geotargeted social media (eg, Facebook) campaign to all LGAs run from Heart Foundation social media accounts and state-wide media in the last 2 months. Where possible, the Heart Matters coordinators were health care professionals who either resided or worked in the areas. Educational content, which was codesigned and pretested with partner organizations and consumers, was based on the Common-Sense Model of Self-Regulation,^23^ addressing ACS risk factors, symptom recognition, and known barriers to EMS use in patients with ACS.^3,8,12,17^ Workshops with consumers and experts were held before trial commencement to gather insights into how to reach culturally and linguistically diverse populations and to evaluate the educational materials and resources. The Heart Attack Action Plans were translated, and the videos were subtitled into the 5 most common languages in the LGAs.

Engagement strategies were implemented under the guidance of an experienced community engagement and project manager (J.W.). Approaches that had previously proven successful in engaging local communities in educational activities by partner organizations were adopted.^24^ The coordinators were encouraged to use their local knowledge and connections, and where possible, collaborate with local government, community leaders, and health service practitioners, to identify community groups, local events, and promotional opportunities. To maximize awareness of Heart Matters activities, local advertising channels, including newspapers, pharmacies, general practices, and a dedicated website, were also used. Coordinators were also asked to document any external heart attack education that they were aware of that occurred in their LGAs.

### Randomization

Clusters were randomized into sequences determining transition from control to intervention using a computer-generated schedule prepared by the study statistician with external oversight to ensure allocation blinding; cluster numbers replaced LGA names for analysis. Two bordering LGA pairs were randomized together to minimize contamination, with an additional LGA included to maintain statistical power (see protocol in Supplement 1 for full details).

### Data Collection and Outcomes

Primary and secondary outcomes, aside from resident surveys, were obtained from administrative datasets and clinical registries, including data provided by the Victorian Department of Health, Services Australia, the Victorian Cardiac Outcomes Registry, and Ambulance Victoria. Data sources are detailed in the protocol (Supplement 1) and undergo internal quality assurance.^16^ Country of birth, sex and gender, Indigenous status, and preferred spoken language were self-reported in the datasets. To examine longer-term effects, the primary outcome was collected for 12 months after the conclusion of the intervention.

The primary outcome was the proportion of patients with ACS transported to public hospitals by EMS, as captured in the externally validated^25^ Victorian Emergency Minimum Dataset. To assess COVID-19 pandemic–related changes, which saw increased EMS use for ACS,^26^ baseline data were examined for the 12 months before trial commencement. Use was slightly higher in intervention regions compared with 2019 (64% vs 61%), but below the state average (70%). We prespecified 27 secondary outcomes examining intended intervention effects (eg, changes to time to health seeking for ACS, ACS symptom knowledge, and heart health checks), and potential system impacts (eg, emergency call and presentations for unspecified chest pain) (Supplement 1).^16^

### Program Evaluation

A detailed program evaluation was conducted alongside the trial, guided by the Reach, Effectiveness, Adoption, Implementation, and Maintenance (RE-AIM) framework,^36^ to assess implementation and inform potential replication and scale-up. The results of this evaluation will be outlined here and published in full elsewhere. Evaluation components included (1) attempted contact and participation rates in education sessions, with reporting on participant numbers, event duration, delivery mode, location, resources used, and session content; (2) use of online resources, tracked by the Heart Foundation (eg, website analytics); (3) lessons learned, as captured in diaries and qualitative interviews with coordinators; (4) intervention reach, measured by the proportion of residents exposed to campaign activities and social media metrics; and (5) acceptability, assessed through participant surveys (Supplement 1) and social media engagement metrics (eg, video viewing duration). These data were descriptively summarized and triangulated to aid the interpretation of trial outcomes.

### Statistical Analysis

All outcomes were compared between the control and intervention periods, excluding data collected during the initial 2-month transition periods. The full statistical analysis plan (Supplement 1) was finalized before the datasets were locked and analysis had commenced. Based on an assumed baseline EMS use of 61%,^17,20^ a sample of 2240 patients with ACS (40 per LGA per 2-month period, excluding the transition periods) would provide 80% power to detect an 11%^17,27^ absolute increase in EMS use (intraclass correlation coefficient = 0.09).^28^ No attrition adjustment was required because complete capture was achieved from routine data.

**Figure 1.**
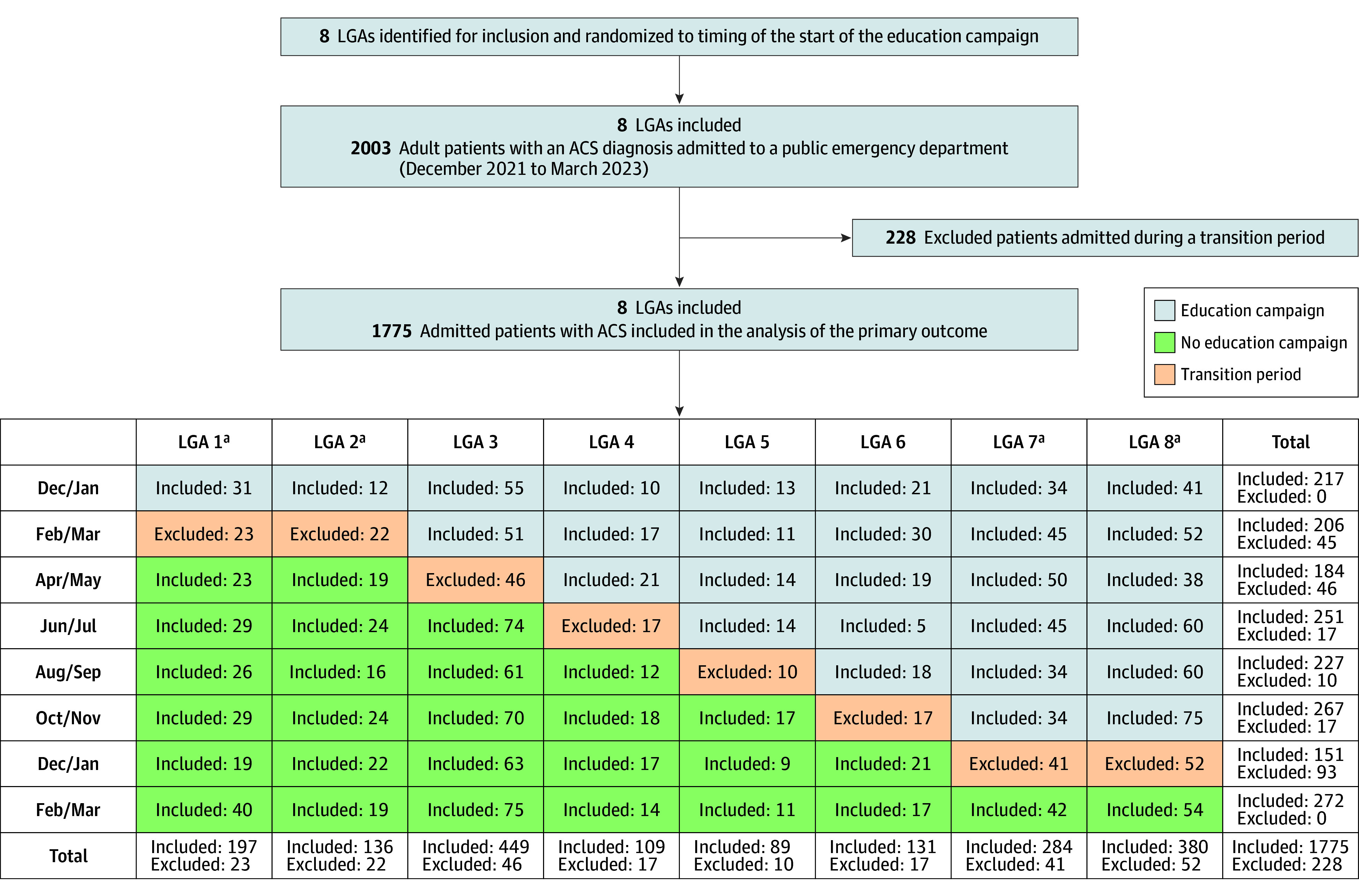
Consolidated Standards of Reporting Trials Diagram ACS indicates acute coronary syndrome; LGA, local government area. ^a^Randomized together due to proximity.

Characteristics of each LGA (Australian Bureau of Statistics) were described. Patient numbers by LGA and period are presented; demographics are summarized by intervention status. The primary analysis assumed implementation as per Figure 1, including all eligible Victorian Emergency Minimum Dataset patients except those admitted during transition periods. Outcomes were analyzed at the patient level using mixed-effects regression models with an identity link function, cluster random intercept and fixed effects for period and intervention, reporting risk differences (RDs) and 95% CIs. The Kenward-Roger correction addressed the small number of clusters.^29^ Odds ratios (ORs) were estimated using logistic regression generalized estimating models with Kauermann-Carroll standard errors. Prespecified analyses assessed alternative correlation structures, adjustment for patient characteristics, subgroup effects (with interaction terms), and time-varying intervention effects.

Secondary binary outcomes were analyzed as per the primary outcome. Count outcomes used Poisson regression models with generalized estimating equations, including period and intervention terms LGA population offset. Continuous outcomes used mixed-effects quantile regression models with period and intervention terms and cluster random intercepts. Analyses were conducted in R version 4.2.1 (R Project for Statistical Computing) using packages^30,31,32,33,34,35^ and Stata version 17 (StataCorp). The secondary outcomes relating to changes in ACS knowledge in the online survey will be published elsewhere. Significance was defined as a 2-sided *P* < .05.

## Results

### Intervention Delivery

The delivery of the intervention (eTables 2-6 in Supplement 2) included 484 in-person sessions reaching approximately 10 088 residents; a mailout to 174 110 households; 17 local media spots; and, in the final months, national mass media (approximately 350 media stories in March 2023) and a geotargeted social media campaign (January to March 2023; estimated reach, approximately 350 000 residents). Online resident surveys at midstudy (818 residents) and end of study (850 residents) timepoints showed that approximately 25% of the respondents in intervention areas recalled seeing or hearing campaign messages in the preceding 3 months. Among those completing evaluation forms following the in-person sessions (6966 of approximately 10 088 residents [69.0%]), most were aged older than 65 years (5100 residents [73.0%]), identified as female gender (4540 residents [65%]), and were Australian-born (4950 residents [71.0%]), varying across LGAs (eTable 7 in Supplement 2). High proportions expressed confidence in key messages (ACS symptom recognition, EMS use, and risk factors) (eFigure 1 in Supplement 2). Coordinators reported that local knowledge and council involvement aided delivery, while summer seasonality, weather, COVID-19, and the severe flooding event reduced opportunities and attendance; community concerns about EMS demand, cost, and waiting times were raised.

### Primary Outcome

**Figure 2.**
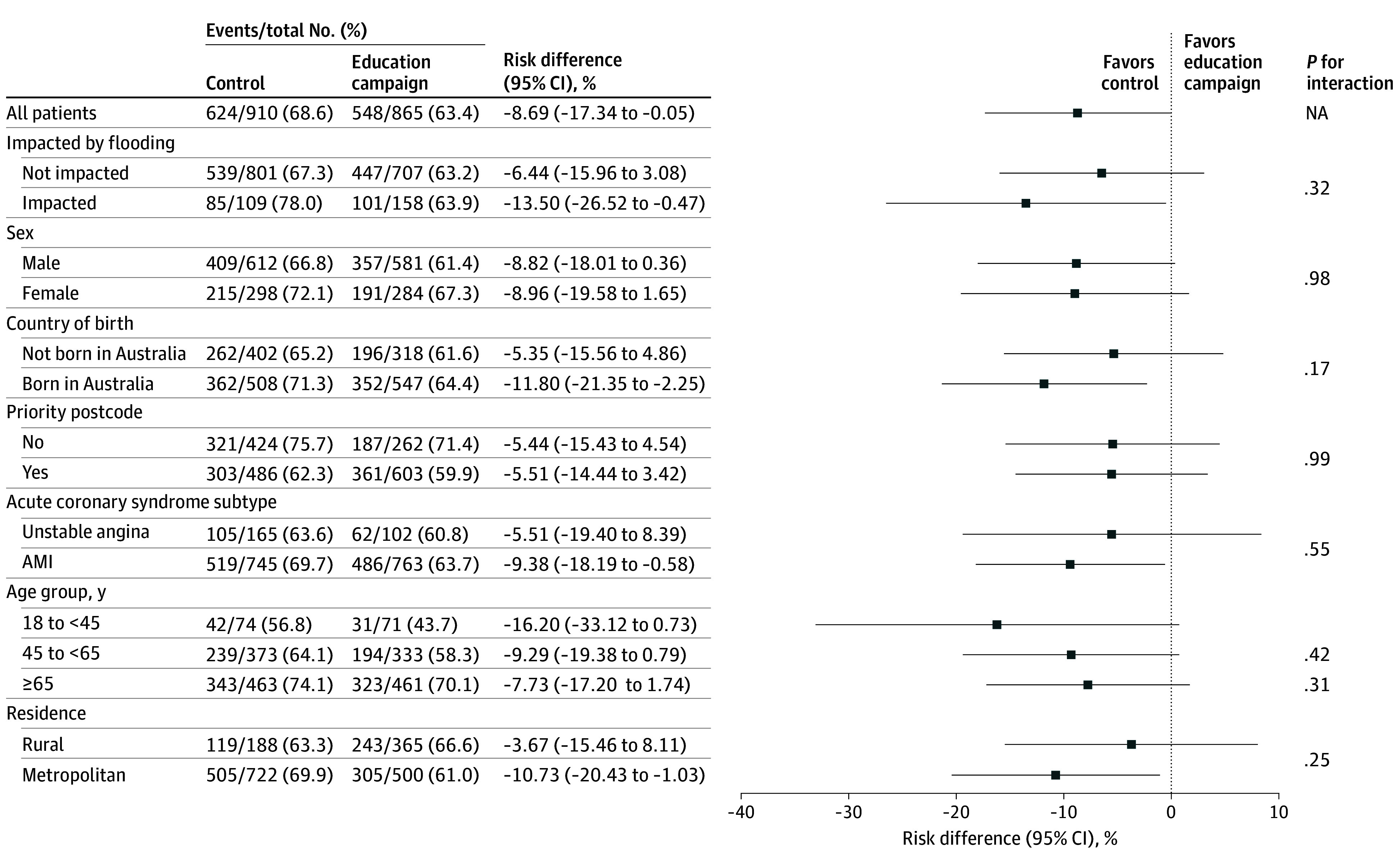
Descriptive Forest Plot of Risk Differences of Emergency Medical Services Use for Each Subgroup AMI indicates acute myocardial infarction; and NA, not applicable.

**Figure 3.**
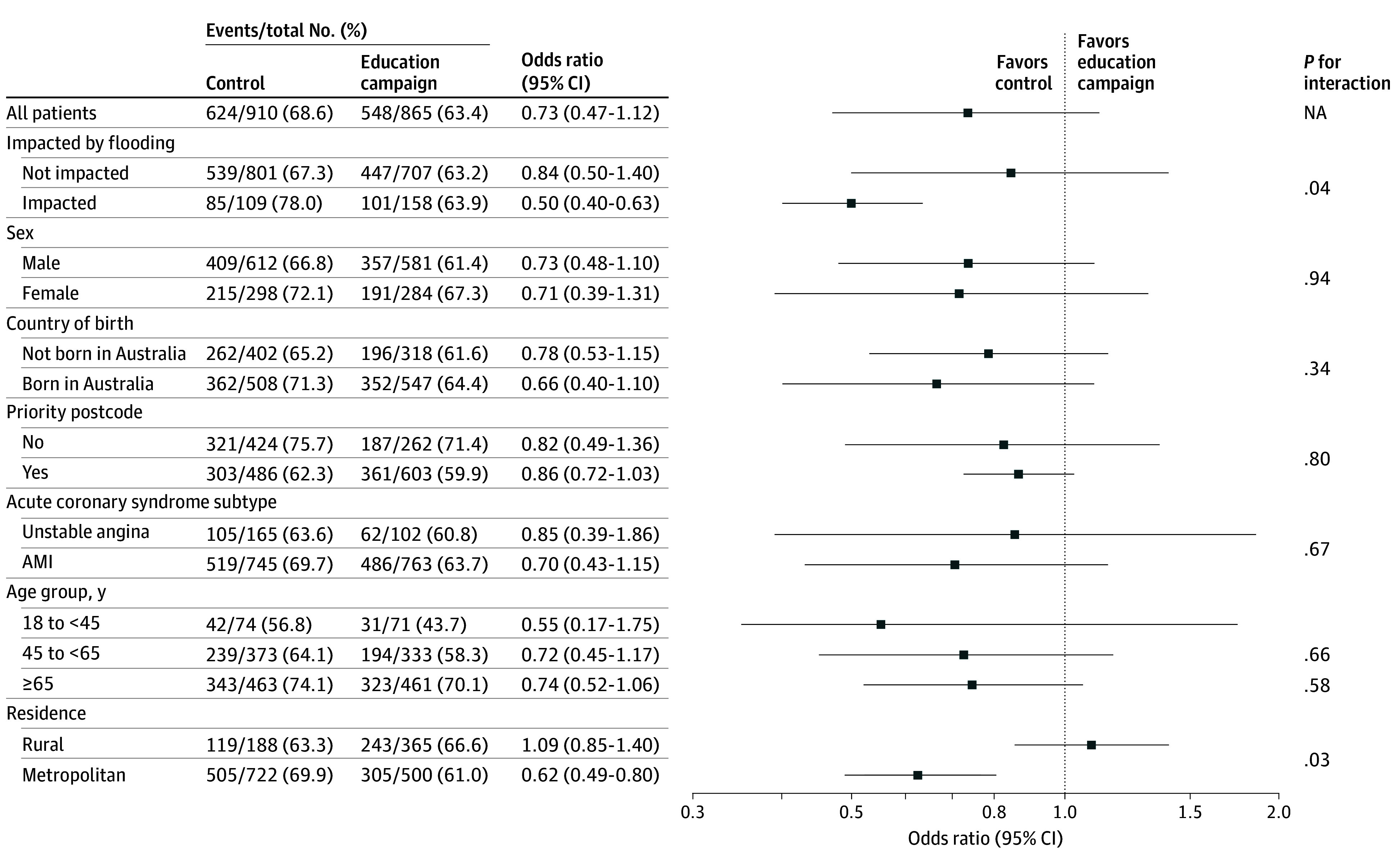
Descriptive Forest Plot of Odds Ratios of Emergency Medical Services Use for Each Subgroup AMI indicates acute myocardial infarction; and NA, not applicable.

Between December 2021 and March 2023, there were 2003 patients with ACS admitted to public emergency departments from the 8 clusters, and 1775 (924 [52.1%] aged ≥65 years; 1193 male sex [67.2%]) were included in the primary analysis, with 910 in the control period and 865 in the intervention period (Figure 1). Compared with the control period, patients admitted during the intervention were more often Australian-born, English-speaking, and admitted with acute myocardial infarction (eTable 8 in Supplement 2). EMS use was unusually high in early control months (December 2021 to January 2022, 158 of 217 individuals [72.8%]) during an Omicron COVID-19 wave, after which it decreased (February to March 2022, 126 of 206 individuals [61.2%]) (Table 1 and eTable 9 and eFigure 2 in Supplement 2). EMS transport to hospital occurred in 624 of 910 patients with ACS (68.6%) in the control period and 548 of 865 patients (63.4%) in the intervention period (adjusted RD, –8.98%; 95% CI, –17.50% to –0.46%; *P* = .04; adjusted OR, 0.67; 95% CI, 0.45 to 1.01; *P* = .05) (Table 2). In subgroups, reductions were greater in metropolitan areas (RD, –10.73%; 95% CI, –20.43% to –1.03%; *P* = .03) and flood-affected periods (RD, –13.50%; 95% CI, –26.52% to –0.47%; *P* = .04), although interactions were imprecise (Figure 2 and Figure 3). There was no evidence of a dose-response or cumulative duration effect of the intervention for EMS use (eTable 10 in Supplement 2), and rates of EMS use remained stable for the 12 months postintervention (eTable 11 in Supplement 2).

**Table 1.** Patients With Acute Coronary Syndrome Arriving at Emergency Departments via Emergency Medical Services by Region and Period

LGA	Patients, No./total No. (%)								
	December 2021 to January 2022	February to March 2022	April to May 2022	June to July 2022	August to September 2022	October to November 2022	December 2022 to January 2023	February to March 2023	Total, 2021 to 2023
1	28/31 (90.3)^a^	NA	13/23 (56.5)^b^	21/29 (72.4)^b^	19/26 (73.1)^b^	17/29 (58.6)^b^	16/19 (84.2)^b^	26/40 (65.0)^b^	140/197 (71.1)^b^
2	10/12 (83.3)^a^	NA	13/19 (68.4)^b^	21/24 (87.5)^b^	8/16 (50.0)^b^	17/24 (70.8)^b^	16/22 (72.7)^b^	12/19 (63.2)^b^	97/136 (71.3)^b^
3	39/55 (70.9)^a^	32/51 (62.7)^a^	NA	40/74 (54.1)^b^	38/61 (62.3)^b^	45/70 (64.3)^b^	44/63 (69.8)^b^	47/75 (62.7)^b^	285/449 (63.5)^b^
4	7/10 (70.0)^a^	8/17 (47.1)^a^	16/21 (76.2)^a^	NA	7/12 (58.3)^b^	11/18 (61.1)^b^	9/17 (52.9)^b^	7/14 (50.0)^b^	65/109 (59.6)^b^
5	9/13 (69.2)^a^	6/11 (54.5)^a^	7/14 (50.0)^a^	7/14 (50.0)^a^	NA	11/17 (64.7)^b^	4/9 (44.4)^b^	6/11 (54.5)^b^	50/89 (56.2)^b^
6	13/21 (61.9)^a^	12/30 (40.0)^a^	14/19 (73.7)^a^	3/5 (60.0)^a^	10/18 (55.6)^a^	NA	13/21 (61.9)^b^	10/17 (58.8)^b^	75/131 (57.3)^b^
7	24/34 (70.6)^a^	32/45 (71.1)^a^	34/50 (68.0)^a^	30/45 (66.7)^a^	23/34 (67.6)^a^	26/34 (76.5)^a^	NA	25/42 (59.5)^b^	194/284 (68.3)^b^
8	28/41 (68.3)^a^	36/52 (69.2)^a^	26/38 (68.4)^a^	39/60 (65.0)^a^	46/60 (76.7)^a^	59/75 (78.7)^a^	NA	32/54 (59.3)^b^	266/380 (70.0)^b^
Total	158/217 (72.8)	126/206 (61.2)	123/184 (66.8)	161/251 (64.1)	151/227 (66.5)	186/267 (69.7)	102/151 (67.5)	165/272 (60.7)	1172/1775 (66.0)

Abbreviations: LGA, local government area; NA, not applicable.

^a^ Control period.

^b^ Intervention period.

**Table 2.** Primary and Secondary Outcomes

Outcome	Dataset used	Participants, No./ total No. (%)		Estimate (95% CI)	*P* value	ICC	Odds ratio (95% CI)	*P* value
		Control	Education campaign					
Primary outcome								
Arrival at ED via EMS (unadjusted analysis)	VEMD	624/910 (68.6)	548/865 (63.4)	−8.69 (−17.34 to −0.05)^a^	.05	0.013	0.73 (0.47 to 1.12)	.80
Arrival at ED via EMS (adjusted analysis)^b^	VEMD	NA	NA	−8.98 (−17.50 to −0.46)^a^	.04	0.014	0.67 (0.45 to 1.01)	.05
Secondary outcomes								
ED presentations for ACS	VEMD	2.9/10 000	3.4/10 000	1.23 (0.62 to 2.45)^c^	.48	NA	NA	NA
ED presentations for unspecified chest pain	VEMD	43.2/10 000	41.5/10 000	1.06 (0.82 to 1.36)^c^	.62	NA	NA	NA
Referred by general practitioner	VEMD	40/910 (4.4)	31/865 (3.6)	0.37 (−3.16 to 3.90)^a^	.84	0.008	1.04 (0.24 to 4.53)	.95
STEMI patient delay time								
Mean (SD), min	VCOR	152.8 (338.8)	223.4 (441.9)	NA	NA	NA	NA	NA
Median (IQR), min	VCOR	52.0 (22.0 to 133.0)	89.0 (44.0 to 207.5)	45.12 (−41.74 to 131.97)^d^	.31	NA	NA	NA
Delay time ≤60 min	VCOR	144/247 (58.3)	77/212 (36.3)	−30.78 (−47.74 to −13.82)^a^	.001	0.008	0.29 (0.10 to 0.83)	.03
All ACS prehospital delay time								
Mean (SD), min	VCOR	400.0 (773.2)	759.3 (1311.5)	NA	NA	NA	NA	NA
Median (IQR), min	VCOR	135.5 (82.0 to 330.0)	229.0 (115.0 to 815.0)	96.22 (−116.03 to 308.47)^d^	.37	NA	NA	NA
Delay time ≤120 min	VCOR	206/444 (46.4)	116/409 (28.4)	−23.14 (−35.98 to −10.30)^a^	.005	0.129	0.31 (0.13 to 0.75)	.02
Suspected STEMI patient delay time								
Mean (SD), min	VASQI	206.7 (474.0)	174.6 (401.7)	NA	NA	NA	NA	NA
Median (IQR), min	VASQI	52.4 (21.3 to 164.4)	55.1 (18.1 to 200.6)	41.87 (−29.21 to 112.95)^d^	.25	NA	NA	NA
Delay time ≤60 min	VASQI	159/288 (55.2)	107/192 (55.7)	−8.64 (−26.16 to 8.88)^a^	.32	0.014	0.92 (0.64 to 1.32)	.58
Suspected STEMI prehospital delay time								
Mean (SD), min	VASQI	279.0 (474.3)	252.3 (402.9)	NA	NA	NA	NA	NA
Median (IQR), min	VASQI	123.1 (91.0 to 247.3)	138.7 (86.5 to 289.9)	51.72 (−24.05 to 127.50)^d^	.18	NA	NA	NA
Delay time ≤120 min	VASQI	130/271 (48.0)	78/181 (43.1)	−21.50 (−40.38 to −2.63)^a^	.03	0.118	0.61 (0.31 to 1.21)	.13
Heart Health Checks	Medicare	11.1/10 000	10.6/10 000	0.66 (0.41 to 1.08)^c^	.08	NA	NA	NA
Survived to discharge OHCA	VACAR	33/485 (6.8)	29/492 (5.9)	0.84 (−4.69 to 6.38)^a^	.75	0.001	1.19 (0.53 to 2.65)	.62
Incidence of OHCA	VACAR	1.5/10 000	1.9/10 000	1.39 (0.80 to 2.81)^c^	.20	NA	NA	NA
Calls to EMS for chest pain	CAD code 10	38.7/10 000	33.2/10 000	0.95 (0.69 to 1.33)^c^	.74	NA	NA	NA
Calls to EMS for non–chest pain	CAD code 10	130.1/10 000	131.9/10 000	1.15 (0.78 to 1.70)^c^	.42	NA	NA	NA

Abbreviations: ACS, acute coronary syndrome; CAD, Computer-Aided Dispatch; ED, emergency department; EMS, emergency medical services; ICC, intraclass correlation coefficient; NA, not applicable; OHCA, out-of-hospital cardiac arrest; STEMI, ST-elevation myocardial infarction; VACAR, Victorian Ambulance Cardiac Arrest Registry; VASQI, Victorian Ambulance STEM Quality Initiative; VCOR, Victorian Cardiac Outcomes Registry; VEMD, Victorian Emergency Minimum Dataset.

^a^ Risk difference.

^b^ Adjusted for age group, sex, country of birth, living arrangements, interpreter required, and ACS subtype.

^c^ Incidence rate ratio.

^d^ Median difference.

### Secondary Outcomes

Among patients with ST-elevation myocardial infarction (STEMI) undergoing revascularization, the intervention phase saw fewer patients seeking medical attention within 60 minutes of symptom onset (144 of 247 patients [58.3%] vs 77 of 212 patients [36.3%]; *P* = .001]) (Table 2); although this was not observed in suspected patients with STEMI transported by EMS (159 of 288 patients [55.2%] vs 107 of 192 patients [55.7%]). Among all patients with ACS undergoing revascularization, the intervention phase saw fewer patients presenting to hospital within 2 hours of symptom onset (206 of 444 patients [46.4%] vs 116 of 409 patients [28.4%]; *P* < .001]). Median times for these 2 delay periods were also longer for the intervention period, but 95% CIs were wide. Secondary outcomes, including non–chest pain EMS calls and emergency department presentations for unspecified chest pain, showed minimal between-group differences, with 95% CIs including null effects.

## Discussion

This stepped-wedge cluster randomized trial provides, to our knowledge, the first randomized evidence evaluating a community-based heart attack education campaign in high-risk regions. EMS use was unexpectedly high (72.8%) during the first 2 control months, coinciding with the Omicron COVID-19 wave and record demand for emergency services.^37^ Immediately after this period, EMS use in nonintervention areas returned to expected levels (61.2%). The intervention period was associated with lower EMS use among patients with ACS, and this effect was consistent across adjusted and subgroup analyses—particularly in metropolitan regions and during a significant flooding event in October 2022, which impacted most of Victoria. Fewer patients with ACS presented within recommended timeframes during the intervention period, although estimates were imprecise with wide confidence intervals.

These findings contrast with prior evidence. A 2020 systematic review^13^ identified 2 randomized community trials.^15,38^ Both trials conducted community education and reported increased EMS use but had very low baseline EMS use and reported no change in prehospital delay times. In contrast, a large, expensive mass media campaign in Australia produced short-term gains in public ACS symptom awareness,^19^ EMS activation,^17,27^ prehospital delay,^3^ and out-of-hospital cardiac arrest rates and survival.^18^ However, these benefits decreased within months of campaign cessation. By focusing on high-risk communities and using a more personalized, locally tailored approach, our intervention aimed to overcome these barriers and achieve a more durable and cost-effective impact.

Although the intervention was well received, particularly the in-person sessions attended by generally older residents, its overall impact was constrained by trial design, funding, and external factors. The stepped-wedge design, while ensuring equity, limited implementation. Social media campaigns were delayed until all clusters crossed over, limiting overall exposure; the 2 clusters randomized last, with the largest populations, also had the shortest intervention periods. Major flooding shifted community priorities, and in-person activities slowed substantially over the summer months as groups paused gatherings. Communities also expressed concerns about EMS costs, long EMS response times, and emergency system demand as reported in the mass media. Cost and proximity to hospital are barriers to EMS use,^8,12^ and session discussions about the need for rapid response, EMS delays, and costs may have inadvertently discouraged EMS use despite increased awareness of its benefits. It is also possible that individuals with high cardiovascular risk may not be engaged with community organizations or social media, which would have limited their exposure to the intervention. In retrospect, targeting entire LGAs was ambitious, particularly in regions with brief intervention windows. The campaign duration for a community intervention without concurrent mass media support was likely too brief; no dose-response effect was observed across increasing intervention periods, although this may have been influenced by unusually high baseline rates in the initial months.

The observed decrease in early treatment-seeking during the intervention is concerning. Post hoc analyses (data not shown) suggest this occurred both during and outside the flooding period. Because the pattern was not seen in the EMS-restricted data, it likely reflects patients who did not activate EMS. Patient and prehospital delay is multifactorial and can be influenced by a range of cognitive (eg, illness representations), emotional (eg, fear, denial, and embarrassment), social (eg, other commitments), and behavioral (eg, wait and see if symptoms get worse and visit local doctor) factors.^8^ The intervention may have inadvertently exacerbated some of these factors. Future interventions should consider this potential unintended effect.

### Strengths and Limitations

Strengths of this study include its large, cluster-randomized design conducted in partnership with ambulance services, the Victorian Government, and the Heart Foundation, with outcomes obtained from population-based registries and datasets. Data from multiple sources allowed comprehensive measurement of both behavior and clinical outcomes.

Limitations include restricting the primary outcome to public hospitals, although more than 90% of patients with ACS in Victoria are treated in the public sector.^39^ The cost of EMS in our region may have impacted the results, and the results may not be generalizable to regions with free EMS. We planned to interview patients with ACS about campaign exposure and its effect, but this was not possible because hospital-based research was suspended during the COVID-19 pandemic. Information from these interviews would have provided important information about the intervention’s reach and impact, as well as insights into persistent barriers. The dataset used to assess delay times in patients with ACS or STEMI was restricted to those undergoing revascularization and may not represent all patients with ACS. Additionally, concurrent local system pressures and environmental events may limit the generalizability of these findings.

However, these results have important implications for policy and practice, particularly as large-scale mass media campaigns may not shift long-term awareness^19^ and behavior.^40^ Our intervention, while delivered in high-risk regions, may not have been sufficiently targeted or sustained to overcome long-standing barriers. Our prior work identified populations with poorer awareness and treatment-seeking behavior,^17,19^ most consistently males younger than 60 years. However, our in-person session predominantly reached older females, suggesting that the delivery methods favored female participation and that alternative approaches may be needed to engage males.

## Conclusions

In this stepped-wedge cluster randomized clinical trial in high-risk communities, an ACS education campaign did not improve outcomes as intended, and EMS use and early treatment-seeking worsened during the intervention period. However, the trial was impacted by external events that may have outweighed intervention effects. Future strategies may need to consider alternate study designs, include other modes of education, and be more targeted and sustained to achieve meaningful change.

## Notes

Supplement 1. Protocol and Statistical Analysis Plan

Supplement 2. eTable 1. Profiles of the eight local government areas in 2021 eTable 2. Engagement strategy and reach overview eTable 3. Coordinators backgrounds eTable 4. Resources used and content eTable 5. In-person session locations, lengths and adaptations eTable 6. Social media campaign eTable 7. The demographics of in-person session attendees based on self-reported data in the evaluation form eFigure 1. Survey results from participants of the in-person sessions eTable 8. Baseline characteristics of ACS patients arriving at emergency departments by emergency medical services (primary outcome) eTable 9. Number of acute coronary syndrome patients arriving at emergency departments via EMS and total number of acute coronary syndrome patients arriving at emergency departments in Heart Matters and Non-Heart Matters LGAS by period eFigure 2. Predictive margins of EMS use proportions at each study period for non-HM and HM LGAs, with 95% confidence intervals eTable 10. Analysis of primary outcome using the outcomes collected during and after the trial, assuming different treatment effects for each number of periods on treatment eTable 11. Number of acute coronary syndrome patients arriving at emergency departments via EMS and total number of acute coronary syndrome patients arriving at emergency departments in Heart Matters LGAs after the trial had ended

Supplement 3. Nonauthor Collaborators

Supplement 4. Data Sharing Statement
