# Supplementary material for: Heart Attack Education and EMS Response in High-Risk, Low EMS Usage Areas: A Stepped-Wedge Cluster-Randomized Trial
Source: JAMA Netw Open. 2026 Apr 27;9(4):e268823. doi: 10.1001/jamanetworkopen.2026.8823 (PMC13122394; doi:10.1001/jamanetworkopen.2026.8823)
Supplement: Supplement 1. — Protocol and Statistical Analysis Plan [file jamanetwopen-e268823-s001.pdf]

## Protocol Changes and Statistical Plan Deviations

### Protocol

1. Protocol -added collection of prehospital delay times from the Victorian Cardiac Arrest Registry (originally planned to collect this data from hospitals and interviews with ACS patients, which was not possible due to the pandemic).
2. The preplanned incremental cost-effectiveness analysis was not undertaken given our results.

### Statistical Plan

1. Section 2.7 typo in Null hypothesis: “This is, the risk difference is equal to 0”.
2. Section 2.7 typo in Alternative hypothesis: “That is, the risk difference is not equal to 0”.

# Heart Matters:

# A cluster randomized controlled trial of heart health education targeting Victorian communities at high risk of Acute Coronary Syndrome

# PROTOCOL

| | |
|---|---|
| **Short title** | Heart Matters |
| **Trial Registration:** | *<Clinicaltrials.gov registration number>* |
| **Protocol Version** | V1.0 |
| **Date** | 03/11/2020 |
| **Stage** | FINAL |



| | |
|---|---|
| **Funding** | NHRMC Partnership Grant #1180282 |
| **Funding Partners** | Monash University<br>National Heart Foundation Australia (Heart Foundation)<br>Victorian Department of Health and Human Services (DHHS)<br>Ambulance Victoria (AV) |



| | |
|---|---|
| **Partner organisations** | Safer Care Victoria<br>Alfred Health: The Alfred Hospital<br>Bendigo Health<br>Northern Health: The Northern Hospital<br>Western Health: Footscray Hospital<br>Western Health: Sunshine Hospital |

**Protocol Amendments**

| Amendment No. | Date of Amendment | Date of Approval |
|---|---|---|
| | | |
| | | |
| | | |

## CONTACTS

| | |
|---|---|
| Sponsor | Monash University<br>School of Public Health and Preventive Medicine |
| Principal Investigator | Associate Professor Janet Bray<br>Monash University<br>School of Public Health and Preventive Medicine<br>Email: Janet.bray@monash.edu |
| Investigator Group | Prof Judith Finn, Curtin University<br>Prof Robyn Clark, Flinders University<br>Dr Ziad Nehme, Ambulance Victoria<br>A/Prof Dion Stub, The Alfred and Western Hospitals.<br>Prof Karen Smith, Ambulance Victoria<br>Prof Clara Chow, Westmead Applied Research Centre, University of Sydney<br>Prof Dominique Cadilhac, Monash University and the Florey Institute of Neuroscience and Mental Health<br>Dr Jessica Kasza, Monash University<br>Prof Peter Cameron, The Alfred Hospital and Monash University<br>A/Prof Arthur Nasis, Cardiac Clinical Network and Safer Care Victoria<br>Prof Ben Smith, University of Sydney<br>Ms Kellie-Ann Jolly, National Heart Foundation<br>A/Prof Nicholas Cox, Western Health<br>Dr Susie Cartledge, Monash University<br>Prof Tony Walker, Ambulance Victoria<br>Dr Voltaire Nadurata, Bendigo Hospital<br>Prof William VanGaal, Northern Health<br>Ms Kelly Donnelly, National Heart Foundation<br>Ms Roni Beauchamp, National Heart Foundation<br>Mr Adam Stormont, National Heart Foundation<br>Ms Lizzie Flemming, Consumer Advisor<br>Dr Alison Beauchamp, Monash University<br>Dr Joosup Kim, Monash University |
| Project Manager | Ms Natasha Dodge, Monash University |

# TABLE OF CONTENTS

LIST OF ABBREVIATIONS .......... 4
SYNOPSIS .......... 5
BACKGROUND AND SIGNIFICANCE .......... 7
AIM .......... 10
OBJECTIVES .......... 11
STUDY DESIGN .......... 11
1. Stepped-wedge cluster randomised trial .......... 12
*Study setting* .......... 12
*Intervention* .......... 12
SW-CRT Outcomes .......... 13
Data collection and data sources .......... 14
Sample size .......... 16
Recruitment .......... 16
Allocation and sequence generation .......... 17
Statistical methods of analyses for the primary and secondary clinical outcomes .......... 17
2. ProCESS Evaluation .......... 18
Process evaluation indicators: .......... 18
3. Cost-effectiveness Evaluation .......... 18
DATA MANAGEMENT .......... 20
Data entry .......... 20
Security and storage .......... 20
Retention and archiving .......... 20
ETHICS AND DISSEMINATION .......... 21
Ethical conduct of the study .......... 21
Protocol amendments .......... 21
Consent .......... 21
Confidentiality .......... 22
Dissemination and authorship .......... 22
COVID-19 Planning .......... 22
STUDY TIMELINES .......... 23
REFERENCES .......... 24
APPENDICES .......... 27

## LIST OF ABBREVIATIONS

| | |
|---|---|
| **ACS** | Acute Coronary Syndrome |
| **AI** | Associate Investigator |
| **AMI** | Acute Myocardial Infarction |
| **AV** | Ambulance Victoria |
| **CAD** | Computer-aided dispatch |
| **CI** | Chief Investigator |
| **DALY** | Disability-Adjusted Life Year |
| **DHHS** | Department of Health and Human Services |
| **ED** | Emergency Department |
| **EMS** | Emergency Medical Services |
| **GP** | General Practitioner |
| **HM** | Heart Matters |
| **HREC** | Human Research Ethics Committee |
| **LGA** | Local Government Area |
| **LHD** | Local Health District |
| **NHFA** | National Heart Foundation Australia |
| **NHMRC** | National Health and Medical Research Council |
| **OHCA** | Out of Hospital Cardiac Arrest |
| **REDCap** | Research Electronic Data Capture |
| **SFTP** | Secure File Transfer Protocol |
| **VACAR** | Victorian Cardiac Arrest Registry |
| **VEMD** | Victorian Emergency Minimum Dataset |
| **VAED** | Victorian Admitted Episodes Dataset |
| **VASQI** | Victorian Ambulance STEMI Quality Initiative |
| **SED** | Socio-economic Disadvantage |
| **SW-CRT** | Stepped-wedge cluster randomised trial |

## SYNOPSIS

| | |
|---|---|
| **Title** | *Heart Matters: A stepped-wedge cluster randomized controlled trial (SW-CRT) of heart health education targeting Victorian communities at high risk of acute coronary syndrome (ACS).* |
| **Aim** | *The study aims to evaluate whether providing a heart health education campaign targeted at communities at highest risk of heart attacks will improve symptom recognition and response.* |
| **Null Hypothesis** | *There will be no change in the proportion of ACS patients arriving via ambulance during the intervention phase.* |
| **Primary objectives** | *1. To increase ambulance use in ACS patients<br>2. To decrease patient and prehospital delay times in ACS patients<br>3. To increase awareness of personal cardiovascular risk and associated factors<br>4. To increase cardiovascular knowledge and confidence to act to heart attack warning signs in adult community members* |
| **Intervention** | *A community heart health education campaign that targets regions at high risk.* |
| **Study Design** | *A stepped-wedged cluster randomised trial of eight local government areas (LGAs) at highest of heart attacks in Victoria, Australia.* |
| **Outcomes** | ***Primary outcome***<br>- *The proportion of ACS patients that present to emergency department (ED) by ambulance*<br><br>***Secondary outcomes***<br>- *Median patient delay time for ACS patients*<br>- *Median prehospital delay times for ACS patients*<br>- *The proportion of ACS patients with patient delay times <60 minutes*<br>- *The proportion of ACS patients with prehospital delay times <120 minutes*<br>- *The proportion of adult members of the community aware of their own heart attack risk*<br>- *The proportion of adult members of the community aware of heart disease as leading cause of death* |

| | |
|---|---|
| | - *The proportion of adult members of the community aware of cardiovascular risk factors*<br>- *The proportion of adult members of the community aware of heart attack symptoms*<br>- *The number of correctly named ACS symptoms.*<br>- *The proportion of adult members of the community confident to respond to heart attack symptoms*<br>- *The proportion of adult members of the community who would use an ambulance in heart attack scenarios*<br>- *Medicare rates of General Practitioner (GP) Heart Health Checks*<br>- *Rates of ED presentations for ACS and unspecified chest pain*<br>- *Incidence of OHCA*<br>- *The proportion of OHCA and ACS survival to hospital discharge*<br>- *The proportion of OHCA survival to hospital discharge*<br>- *The proportion of ACS survival to hospital discharge*<br>- *Rates of calls to ambulance for chest pain*<br>- *Rates of calls to ambulance for non-chest pain*<br>- *The cost effectiveness of the intervention relative to the control periods*<br><br>***Process outcomes***<br>- *Acceptability of education by participants attending sessions*<br>- *Participation rates in education sessions*<br>- *The proportion of adult members of the community exposed to the Heart Matters intervention*<br>- *The characteristics of those exposed to the Heart Matters intervention*<br>- *The proportion of ACS patients reporting that education was beneficial for treatment seeking*<br>- *Lessons learnt by Heart Matters coordinators* |
| **Sample Size** | *2240 ACS patients (primary outcome)* |
| **Population** | *Eight LGA's in Victoria, Australia selected from 16 LGA's with highest ranking for age-standardised rates of heart attacks.* |
| **Study Duration** | *48 months* |

## BACKGROUND AND SIGNIFICANCE

### Acute Coronary Syndrome (ACS) is a leading cause of death in Australia

ACS, of which the most severe form is a heart attack, is usually caused by coronary heart disease –the leading cause of death in Australia.[1] An estimated 105,000 Australians are hospitalised with ACS every year.[2] *However, ACS is preventable and treatable –if detected and responded to early.*

### Benefits of community cardiovascular risk assessments

ACS is associated with modifiable risk factors that are now common in the Australian community (e.g. high blood pressure, high blood cholesterol, smoking, diabetes, overweight/obesity, and physical inactivity).[3] Community risk screening interventions typically involve assessing members with no known ACS and referring patients identified "at potential risk" to their general practitioners (GPs) for further screening and management. Published studies of public screenings have found high rates of new identified risk factors (in as many as 66% of people screened), and is associated with improved outcomes if subsequently treated.[4, 5] Public screening and referral to GPs are now supported within the Australian health care system, with the Federal Government's recent implementation of a Medicare item for GPs to conduct Heart Health Checks in adults aged over 45 years (35 years for Indigenous Australians).

### Benefits of early treatment and ambulance use for ACS

International guidelines strongly recommend emergency treatment for ACS, which include medication (aspirin and thrombolysis) and procedures (revascularisation) to reopen the blocked blood vessels in the heart (collectively referred to as 'reperfusion therapies').[6, 7]

The benefits of reperfusion therapy are maximised when they are administered *within the first 90 minutes following symptom onset (Figure 1)*[8] –these include less heart damage, fewer complications and better short and long-term outcomes.[9-11]

*Figure 1. The relationship between time and ACS outcomes.*[8]

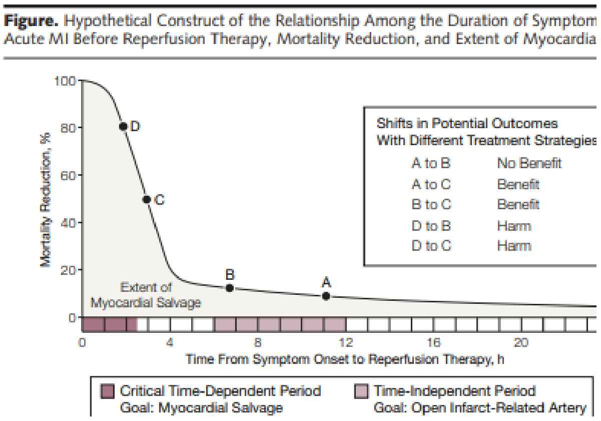


Reperfusion therapies are not available at every hospital, which is one of the reasons why ambulance use for ACS is recommended internationally.[7]

Paramedics are trained to detect and provide emergency treatment for ACS, and for the potentially life-threatening complications associated with this condition (e.g. cardiac arrest).[12] Transport by ambulance also ensures suspected ACS patients present to hospitals with revascularisation capabilities, and some ambulance services even initiate thrombolysis during transport.[12] Ambulance use is associated with higher rates of reperfusion therapy, the timeliness of its administration, and improved patient outcomes.[13-15]

**Known factors associated with patient delays and barriers to accessing ambulance care**
To receive optimal outcomes, a person suffering ACS symptoms needs to quickly recognise their symptoms and immediately call an ambulance to receive timely emergency care and ensure transport to a hospital offering revascularisation.[16] *Unfortunately, few ACS patients present to hospital rapidly or by ambulance. Research conducted by CIs/AIs show only 48% of ACS patients recognise their symptoms as heart related, 45% immediately call an ambulance, and only 34% arrive at hospital with two hours of symptom onset.*[16] This work also found that *62% of ACS patients stated their experience was different to what they expected a heart attack to be like.*[16] In addition, the majority of ACS deaths occur in the community, usually before the activation of the emergency system and after hours of typical ACS warning symptoms.[17]

Research driven by illness behaviour theory[18] has uncovered many patient factors that are associated with longer ACS patient delay and failure to use an ambulance.[16, 19, 20] This research suggests major barriers are poor recognition of symptoms as being heart-related, seeking alternate health advice from family members and GPs, and not considering their symptoms serious enough to warrant ambulance care.[3, 20] <u>All factors which are amendable to education.</u>

**Australian campaigns**
The Heart Foundation has previously used public mass media advertisements to increase the Australian public's awareness of ACS symptoms and the need for an urgent response. Evaluations of such campaigns (e.g. the Warning Signs campaign), led by CI/AIs, have shown significant success with associated improvements seen in the public's symptom knowledge, response to symptoms (timeliness and ambulance use) and in prehospital deaths.[16, 21-23]

However, mass media campaigns are expensive, not sustainable and the long-term gains are unknown.[24, 25] For example, the Warning Signs campaign cost an estimated $16 million, ran intermittently between 2009 and 2013, and, as funding was reliant on donations and local government contributions, had variable exposure across the country. Just over half (55%) of Australians[23] and two-thirds (64%) of ACS patients[16] recalled seeing the advertising during the campaign period. The long-term effectiveness of this campaign is unknown, but available data from other campaigns suggest regular and sustained advertising is necessary.[26] There are also concerns about the potential for increased use of emergency services by patients without ACS (i.e. the "worried well") as a result of such campaigns.[25] Data from the Warning Signs campaign suggest this number may be significant.[22] This is an important consideration as emergency services are finite resources, and attending to high numbers of non-ACS patients may well divert resources away from the people with medical emergencies.

**Previous research**
The largest targeted education study to date, the REACT trial, ran an 18-month community and patient education intervention (mass and small media, community and patient group education) in 20 USA communities.[27] While REACT showed a significant increase in ACS symptom knowledge and ambulance use, there was no impact on prehospital delay times.[27]

However, the authors acknowledged that prehospital delay was already low at baseline in their targeted communities.

There have been numerous reviews of ACS education campaigns and interventions which have shown varied impact on patient delay and ambulance use.[24, 28] There is significant variation seen across this body of evidence, including different: health care settings;[16, 21, 22, 28] interventions and exposure; baseline values of outcomes; and the timing and methods of data collection.[24, 28] *Importantly,* most of these studies have used total *prehospital delay* (e.g. time from symptom onset to hospital arrival) as the primary outcome. However, illness behaviour theory suggest a more accurate measure of patient response is *patient delay* – which is the time from symptoms onset to the decision to seek medical attention (e.g. calling an ambulance). It has also been suggested by leading investigators of large community trials that interventions are best carried out in *"populations where risk and rates are high or highest, and where the health promotion message has not penetrated, that is, where knowledge, sophistication, and rapid change in health behaviours have not arrived."*[27, 29]

Education targeting regions at highest risk of ACS may be a more efficient and cost-effective approach, but is understudied internationally. A study conducted in the early 80's targeted a small high-risk ACS rural region without "a clear pattern of improvement".[30] However, this study was conducted without the wealth of knowledge on ACS patient delay [31] and illness behaviour theory;[18] which enables the development of an evidence-based, effective intervention.[19, 24] The identification of regions at highest risk of ACS in Australia is now available as a result of the Heart Foundation's Heart Maps.[32]

**Heart Maps**

The Heart Maps[32] were developed to identify the variation in heart disease across Australia and to highlight areas in greatest need of interventions. In Victoria, these maps include data collected by the DHHS and Ambulance Victoria, and map rates of ACS incidence, mortality, cardiac arrest and risk factors by local government areas (LGAs). Figure 2 shows the Victorian LGAs at lowest (pale yellow) and highest (brown) risk of heart attacks.

*Figure 2. Victorian Heart Map*

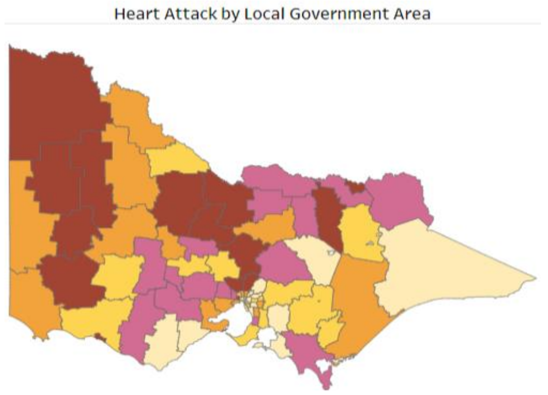


**Formative and Preliminary Heart Matters research**

In developing our proposal, the HM Partners examined some of the underlying characteristics of Victorian high- and low-risk populations. Using available data sources, we compared the 16 LGAs at highest risk of heart attacks to the 16 LGA's at lowest risk. There was no difference in the sex or age distributions of the respective populations; however, residents of high-risk LGAs were more likely to be born in Australia (particularly rural LGAs) and have lower levels of education and socio-economic status (data from ABS). Compared to low-risk regions, high-risk LGAs also had:

- Higher rates for heart failure, out-of-hospital cardiac arrest (OHCA), and cardiovascular mortality (Heart Foundation Heart Maps).

- Less cardiovascular knowledge: risk factors (e.g. poor diet: 50% vs 56%; smoking: 19% vs. 23%; difference between a heart attack and a cardiac arrest: 23% vs. 43%, Heart Foundation Heart Watch).
- Higher self-reported ACS risk factors: hypertension and obesity, lower fruit and vegetable intake, and poorer overall health (DHHS Health survey).
- Lower rates of ambulance use for ACS: Odds Ratio 0.86 (95% CI: 0.83-0.91, p<0.001) after adjusting for differences in baseline characteristics known to predict ambulance use (DHHS).

*Targeting these high-risk regions with a* ***community-based heart health education*** *seems a more logical and efficient use of funding and resources than large mass media campaigns.*

## AIM

The aim of the study is to evaluate whether providing a targeted heart health education campaign to 8 LGAs identified at high risk of heart attacks will improve ACS patient's symptom recognition and response in these regions. An overview of the Heart Matters Partnership is given in Figure 3.

Figure 3: Overview of the Heart Matters Partnership.

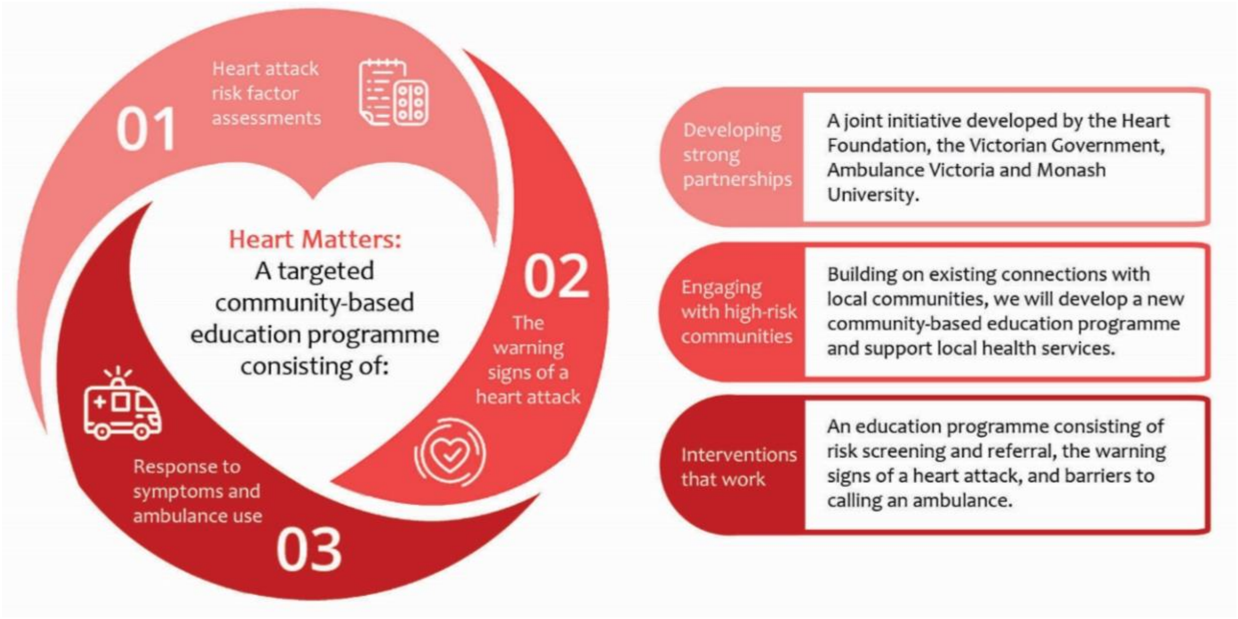

# OBJECTIVES

### Primary objectives

To determine if targeted heart health education will:
1. Increase ambulance use in ACS patients;
2. Decrease patient and prehospital delay times in ACS patients;
3. Increase awareness of personal cardiovascular risk and associated factors in adult community members; and
4. Increase cardiovascular knowledge and confidence to act to heart attack warning signs in adult community members.

### Secondary objectives

1. To determine if targeted heart health education:
    - Reduces the incidence of out-of-hospital cardiac arrest;
    - Improves survival in OHCA patients;
    - Improves survival in ACS patients;
    - Improves survival and ACS patients;
    - Increases presentations to ED for ACS and unspecified chest pain; and
    - Increases the rates of calls to ambulance for chest pain and non-chest pain.
    - Increases the rate of Heart Health Checks.

2. To evaluate the implementation of the intervention by determining:
    - Participation rates in education and screening;
    - Reach of the intervention to community members;
    - Barriers and enablers for heart health educators/coordinators;
    - Barriers and enablers for community members;
    - Levels of exposure to the Heart Matters intervention;
    - Demographics of those who engaged with the Heart Matters intervention;
    - Reported use of the Heart Matters education messages in relation to pre-hospital delay and behaviour; and

3. To determine the cost effectiveness of the targeted intervention by completing an economic evaluation

# STUDY DESIGN

The main study will use a stepped-wedge cluster randomised trial (SW-CRT) to evaluate the effectiveness of the intervention. Concurrently we will also conduct a process and economic evaluation.

## 1. STEPPED-WEDGE CLUSTER RANDOMISED TRIAL

The stepped-wedge design is a uni-directional cross-over design – where the randomisation element is when the cluster crosses-over to the intervention following a control period.

*Study setting*

The study will be undertaken in eight LGAs in Victoria (3 in greater Melbourne and 5 in regional Victoria.

These LGAs were selected from the list of the 16 highest-risk Victorian LGAs (highest ranking for age-standardised rates of heart attacks) generated from the Victorian Heart Maps.[32] These LGAs were selected to meet the study's sample size requirements based on admission rates for ACS from the Victorian Emergency Medicine Dataset (VEMD). Estimates, from the 2013-2015 VEMD data, suggests 1,635 ACS events occur in these LGAs annually (annual range across LGAs=102 to 360).

*Table 1. Profiles of the 8 LGAs to be targeted (*SED= Index of Relative Socio-economic Disadvantage).*

| LGA name | Area km2 | Residents | Index SED* | Median age | Males (%) | Born overseas (%) |
|---|---|---|---|---|---|---|
| LGA1 | 489 | 207058 | 991 | 34 | 50 | 36% |
| LGA2 | 503 | 207041 | 947 | 33 | 50 | 36% |
| LGA3 | 31 | 86942 | 995 | 34 | 51 | 40% |
| LGA4 | 2999 | 112267 | 981 | 38 | 49 | 8% |
| LGA5 | 22082 | 54658 | 935 | 40 | 50 | 11% |
| LGA6 | 432 | 40100 | 977 | 36 | 50 | 10% |
| LGA7 | 4518 | 37595 | 967 | 45 | 50 | 7% |
| LGA8 | 120.7 | 33655 | 986 | 40 | 50 | 8% |

*Intervention*

To meet the objectives of the study, the Heart Foundation will employ HM coordinators for each of the eight high-risk LGAs to organise and deliver our HM education program using HM materials and Partner resources.

*Heart Matters Coordinators*

HM coordinators will be appropriately qualified (e.g. nursing, health promotion) and based in each LGA at a local ambulance branch. They will undergo HM training by investigators to provide and coordinate risk screening and education sessions. They will complete daily activity reports online and be supervised by CIA Bray, the HM Project Manager and Heart Foundation appointed staff. Coordinators will review activities weekly with the HM Project Manager to ensure adherence to protocol.

*Education materials and resources*

Once an LGA crosses over into the intervention phase, a launch event will be held and the HM coordinator will start delivering community education (Table 2). This education will address each of the study's objectives using varying modes of delivery (e.g. in person* and digital). Partner resources will be used (e.g. Heart Foundation's Heart Attack Action Plan – available in multiple languages and magnetic to keep on fridge). HM resources used by HM Coordinators (e.g. videos and presentations) will be developed from existing resources and tested with Partners and collaborators.

* All education will be online (e.g. Zoom webinars) if Government restrictions apply due to the COVID pandemic.

*Table 2. Proposed methods of HM education.*

| |
|---|
| HM coordinator education:<br>• Face-to-face/digital "Know your risk" screening and information sessions.<br>• Face-to-face/digital heart health education sessions with community groups (e.g. Seniors Clubs, Church groups, Lions Rotary, Country Women's Associations, Men's Sheds, Neighbourhood/Community Houses).<br>• Face-to-face/digital targeted education (e.g. face-to-face/digital education seminars for ACS patients and families).<br>• Webinar education sessions after-hours for public and patients.<br>• Nurse Cora -Avatar teaching App[3]: an 8 minute evidence based educational app designed for low health literacy and when English as a second language. The Avatar can also be electronically programmed to speak 144 languages. Features quizzes to engage learners to test knowledge and response to ACS symptoms. |
| Regional events:<br>• Launch event –involving all Partners, local health care professionals, local heart attack survivors and media.<br>• Local media advertisements of sessions and heart health education (e.g. local magazines).<br>• Website – resources, events, programs and frequently asked Q&A.<br>• Local promotion of existing programs –e.g. Walking Program.<br>• Geo-targeted social media advertising with Heart Foundation's Heart Age Calculator and Warning Signs.<br>• Web banner advertising on local organisations/business sites. |

HM coordinators will use local and Partner resources (e.g. speakers) as needed:

- Heart Foundation: Regional Coordinators; Health Professional Volunteers Register; Heart Foundation funded Scholars and Fellows; heart attack survivors; printed and electronic resources; and existing programs.
- Ambulance Victoria: regional Paramedic Community Support Coordinators; and paramedics on light-duties.
- Local: Cardiology/emergency health professionals; medical/nursing/paramedic students.

## SW-CRT Outcomes

The primary and secondary clinical outcomes as listed in Table 3 will be provided to the investigators by the data sources for all Victorian LGAs over the 16-month study period and for one-year after the intervention period to examine the longer-term impact. Data for the whole of Victoria is required to enable a secondary analysis of non-study LGAs. The main primary outcome is the proportion of ACS patients that present to ED by ambulance.

Data collection and data sources

<u>All outcome data are already currently being collected by the data sources </u>listed in Table 3 and described below. Data are required for the entire 16-month study period (main study) and for 12-months after (sustained effect). <u>All data will be provided to Monash University in a de-identified format</u>. Data will be sent to Monash University electronically via a secure file transfer. Data will be provided under a waiver of consent (see ethics below).

*Table 3. Study Outcomes*

| Primary Outcome | Data Sources/Agency |
|---|---|
| The proportion of ACS patients that present to ED by ambulance. | Victorian Emergency Minimum Dataset (VEMD)/ Department of Health and Human Services (DHHS) |
| Secondary outcomes | |
| The median ACS patient delay time (time from symptom onset to decision to seek medical attention). | The Victorian Ambulance STEMI Quality Initiative (VASQI)/ Ambulance Victoria |
| The proportion of ACS patients with patient delay times <60 minutes | VASQI/Ambulance Victoria |
| The median ACS patient prehospital delay time (time from symptom onset to arrival at hospital). | VASQI/Ambulance Victoria |
| The proportion of ACS patients with prehospital delay times <120 minutes | VASQI/Ambulance Victoria |
| The proportion of adult members of the community who are aware of their own risk of heart attack. | Heart Watch Public Survey/Heart Foundation |
| The proportion of adult members of the community who identify heart disease a leading cause of death | Heart Watch Survey/Heart Foundation |
| The proportion of adult members of the community who identify cardiovascular risk factors | Heart Watch Survey/Heart Foundation |
| The number of correctly named cardiovascular risk factors by adult members of the community | Heart Watch Public Survey/Heart Foundation |
| The proportion of adult members of the community aware of heart attack symptoms | Heart Watch Public Survey/Heart Foundation |
| The number of correctly named ACS symptoms by adult members of the community | Heart Watch Public Survey/Heart Foundation |
| The proportion adult members of the community who are confident about what they would do if experiencing a heart attack | Heart Watch Survey/Heart Foundation |
| The proportion adult members of the community who correctly state they would call an ambulance for two heart attack scenarios. | Heart Watch Survey/Heart Foundation |
| Rates of Medicare claims for General Practitioner (GP) Heart Health Checks | Medicare data/Medicare |
| Proportion of ED presentations that are ACS | VEMD/DHHS |
| Proportion of ED presentations that are unspecified chest pain | VEMD/DHHS |
| Proportion of ACS ED presentations via GPs | VEMD/DHHS |

| | |
|---|---|
| Rates of ACS survival | Victorian Admitted Episodes Dataset (VAED)/DHHS |
| incidence of out-of-hospital cardiac arrest | Victorian Cardiac Arrest registry (VACAR)/Ambulance Victoria |
| Rates out-of-hospital cardiac arrest survival | VACAR/Ambulance Victoria |
| Proportion of chest pain (event type 10) calls to ambulance | Electronic computer-aided dispatch (CAD) records/ Ambulance Victoria |
| Proportion of non-chest pain emergency calls to ambulance | Electronic computer-aided dispatch (CAD) records/Ambulance Victoria |

**The Victorian Emergency Minimum Dataset (VEMD)** is a state-wide emergency dataset collected for all presentations to public emergency departments and maintained by the Victorian Government's Department of Health and Human Services. Currently, VEMD data, which is collected by ED staff (including clerical and health professionals), includes demographic (e.g. age, sex, usual place of residence), administrative (e.g. triage code, destination from ED) and clinical data (e.g. primary diagnosis). This dataset is used for epidemiological purposes, health services planning and coordination; policy assessment and formulation; and clinical research and quality improvement.[33]

**The Victorian Admitted Episodes Dataset (VAED)** comprises demographic, clinical and administrative details for admitted episodes of care occurring in Victorian hospitals, rehabilitation centres, extended care facilities and day procedure centres. VAED data is used to provide equitable funding to public hospitals under the casemix system, support health services planning, policy formulation and epidemiological research, and meet national data reporting requirements.[34]

**The Victorian Cardiac Arrest Registry (VACAR)** records details of all OHCA events where EMSs are in attendance. In-field treatment data are captured electronically using computer tablets operated by paramedics. A highly sensitive search filter is used to identify potential cardiac arrest cases from a database of electronic patient care records. Potentially eligible cases undergo manual review by registry personnel, and cases meeting eligibility criteria are entered into the registry extracting over 150 data points including the Utstein-style descriptors. Complete case ascertainment is assured through the review of computer-aided dispatch records, emergency call logs, and paper-based treatment records. Paramedics are also required to report cardiac arrest cases and submit electrocardiogram recordings.[21]

**The Victorian Ambulance STEMI Quality Initiative (VASQI)** is a clinical quality registry that aims to facilitate quality improvement by collecting and monitoring data regarding the treatment and outcome of paramedic suspected STEMI cases. The VASQI incorporates both pre-hospital clinical and operational data, and hospital follow-up data. Paramedic suspected STEMI cases are housed in the VASQI with data being drawn from Ambulance Victoria's Communication centre dispatch records, Emergency Medical Services (EMS) patient care records, and from Team Manager review of STEMI cases. Hospital diagnosis, treatment and

outcome data for paramedic suspected STEMI cases identified in VASQI are linked from the Victorian Cardiac Outcomes Regsitry (VCOR) at Monash University and by collaboration with Victorian public hospitals with the capability of performing percutaneous coronary intervention (PCI).

**Ambulance Victoria keep Electronic Computer Aided Dispatch (CAD)** records for all emergency calls to ambulance. This dataset includes date and chief complaint (e.g. chest pain).

**Medicare** provides access to medical and hospital services for all Australian residents and certain categories of visitors to Australia. The Medicare Benefits Schedule (MBS) list services that are subsidised by the Australian Government under Medicare. Reports can be collated providing anonymised demographic variables on Items in the MBS Schedule. Medicare Items 699 and 177, will support patients to access a heart health assessment through a doctor in a general practice. Reports will be collated on use of these item numbers across local government areas during the intervention period.

**The Heart Foundation's "Heart Watch" Survey** is a quarterly survey conducted using a purposive, non-probability sampling method with quotas for age, gender and area of residence in order to reflect the characteristics of the wider Australian population. Respondents of the survey belong to an online survey panel.[20]

## Sample size

The stepped wedge design in the schematic (a six-sequence stepped wedge design, with an implementation period and two clusters assigned to the first and last sequences) will give 80% power to detect a change in the proportion of ACS patients in the eight LGAs arriving via ambulance by 11%, from 61% to 72%, with a two-sided significance level of 5%. This detectable difference depends on the number of patients expected to be observed in each cluster (average of 40 patients per LGA in each two-month period; a total of 2240 patients) and on the intra-cluster correlation, estimated to be 0.09 using data from the VEMD (2011-2015). Examination of these aforementioned data indicated that more complex correlation structures, with decaying correlations over time, were not necessary.[35] We used the Stepped Wedge Stata Program for the sample size calculation.[36] No adjustment was made for LGA or participant attrition due to the outcomes being those routinely collected by health services.

## Recruitment

*Engagement strategies with communities*

Successfully used methods, by the Heart Foundation, will be employed to engage with local communities, including forming local HM Coalitions. Prior to commencement of the study, the CIA, the Project Manager, Partner Investigators and local collaborators (AIs) will set up coalitions in each LGA to engage local stakeholders. We will approach Local Government, community leaders and health service providers (Hospitals, Cardiac Rehabilitation Centres, General Practice Networks, Pharmacies) in each LGA. We will identify possible community

groups, local events, promotion opportunities and other opportunities to assist with the intervention delivery. We will advertise HM activities locally (e.g. in newspapers) and on a dedicated HM website.

### Allocation and sequence generation

Over the 16-month study period, the eight LGAs will move into the intervention phase at two month intervals. As four LGAs are in close proximity (Whittlesea-Hume; Greater Bendigo-Campaspe), these LGAs will switch from control to intervention periods at the same time to avoid possible contamination. For planning purposes, allocation and sequence generation has already been performed. Allocation of randomisation was performed blinded, by the CIA, study statistician and overseen by an independent investigator. This was performed by CIA allocating each LGA a study number from 1 to 8, which was then sent to the independent investigator. Following this the study statistician generating the randomisation sequence with the numbers 1 to 8, this information was then linked back to the LGA allocation numbers (Table 4).

*Table 4. Allocation and sequence generation.*

| | Time Period | | | | | | | |
|---|---|---|---|---|---|---|---|---|
| LGAs | T1 | T2 | T3 | T4 | T5 | T6 | T7 | T8 |
| Bendigo | | X | | | | | | |
| Campaspe | | X | | | | | | |
| Maribyrnong | | | X | | | | | |
| Wodonga | | | | X | | | | |
| Warrnambool | | | | | X | | | |
| Mildura | | | | | | X | | |
| Whittlesea | | | | | | | X | |
| Hume | | | | | | | X | |

*X = Launch Event*

*T = time period, two months in duration*

*Proposed timeline for intervention. Light grey cells – control period; medium-grey cells- transition period; dark grey cells – intervention period.*

### Statistical methods of analyses for the primary and secondary clinical outcomes

Results from the trial will be reported according to the CONSORT extension for stepped wedge cluster randomised designs.[37] LGA and patient demographics will be described by LGA and period. All available primary and secondary outcomes will be analysed at the patient level using mixed-effects logistic regression models with a random intercept for cluster and fixed effects for each period and for the intervention. The Kenward-Roger correction will be applied to adjust for the small number of LGAs.[38] Since there may be imbalance between the LGAs with respect to various baseline characteristics, in a secondary

analysis we will adjust for potential confounders known to influence ambulance use that are available in the VEMD (e.g. age, sex, Australian born). Estimated effects will be expressed as odds ratios and as risk differences, with 95% confidence intervals. A secondary analysis will include an interaction between time and treatment to investigate a cumulative effect of the intervention over time. It has been shown that misspecification of the within-cluster correlation structure can lead to confidence intervals of incorrect widths,[39] so sensitivity analyses allowing for more complex within-cluster correlation structures[35] will be considered. Analyses will be conducted in Stata and/or SAS as appropriate.

We will also perform subgroup analyses (e.g. age, sex, country of birth) across the primary and secondary outcomes.

## 2. PROCESS EVALUATION

The process evaluation aims to determine whether program activities have been implemented as intended and to provide details of the intervention of allow their replication. This will use mixed methods including online reports, qualitative interviews, and evaluation surveys and will be reported according the TiDier Checklist.[40]

Process evaluation indicators:

- Participation rates in education and screening: Participation rates in education and screening: HM coordinators will complete an online report for every session – recording number of participants, duration of event, mode of delivery, location, resources used, and content. Heart Foundation will collect website hits and use of online resources (e.g. Heart Age Calculator).
- Lessons learnt: HM coordinators will keep a diary to document barriers and facilitators and we will conduct qualitative interviews with HM coordinators at the end of the study to elucidate key themes.
- Acceptability to individuals: we will conduct brief de-identified session evaluation surveys at the end of every session.
- Reach: ACS patients and Heart Watch participants for the intervention LGAs will be asked if they had participated in any Heart Matter education events, seen and made use of intervention materials.
- Adoption and sustainability by organisations: we will survey partner organisation one year after intervention concludes to see if they have adopted any methods used in this study.

## 3. COST-EFFECTIVENESS EVALUATION

The economic evaluation is conducted as a sub-study to the step wedge cluster randomised controlled trial. The perspective of the economic evaluation is the health care sector and the reference year for costs will be 2022.

A cost consequences analysis and a simulated cost-effectiveness analysis will be undertaken for the economic evaluation of the Heart Matters campaign.

The economic evaluation will be written up based on the consolidated health economics reporting standards[41] and any relevant checklists for reporting economic evaluations of trials using a randomised cluster stepped wedge design. For example, Lung and colleagues have recommended that researchers account for the following in their statistical analyses: correlation between costs and outcomes (individually and within clusters) and in the estimation of incremental cost-effectiveness, the need to make appropriate assumptions about the distribution of outcomes and costs, and adjusting for time-effects (including interactions with intervention effectiveness).[42] These steps are necessary since accounting for secular and within-cluster trends is an additional complexity that needs to be properly accounted for in a stepped-wedged design.[42]

***Costs:***

The costs of providing the program (HM coordinators, education packages) will be described. Health sector costs estimated will include those related to the use of ambulances, hospital emergency presentations and admissions as well as the Medicare rebates for Heart Health Checks. Cost-offsets are the costs of disability, deaths or future events avoided and will be estimated based on published cost-of-illness studies or best available evidence that is relevant to the Victorian context.

Costs will be adjusted to the same reference year because of the 16-month study period.

***Cost consequences analysis:***

Cost consequences analysis will be used to describe the expected costs and benefits from the Heart Matters campaign, including community cardiovascular risk assessments, survival, out-of-hospital cardiac arrest incidence, and cost offsets.

The steering committee for the Heart Matters campaign will be asked to define the most relevant clinical, process and economic outcomes to be reported in the cost consequences analysis.

A cost consequences analysis is preferred given the nature of the intervention and the type of data being used to determine the primary and secondary clinical and process outcomes. In this type of economic analysis all the relevant health and non-health effects across different sectors can be reported.[43] This is useful when different outcomes cannot be incorporated into an index measure and there are several important outcomes of relevance to decision-makers enabling these to be assessed transparently against the costs of an intervention.

***Simulated cost effectiveness analysis:***

The cost-effectiveness of the Heart Matters campaign will be assessed using a simulation model. Health sector costs and disability adjusted life years (DALYs) avoided before and after the delivery of the intervention will be estimated. If appropriate, the incremental cost per DALY avoided from the Heart Matters campaign compared to usual care will be estimated.

Where estimates of resource use are derived from the study, these estimates will be obtained by using mixed-effects logistic regression analysis consistent with that conducted for the primary and secondary outcomes of this study.

Since the primary health benefit is expected to be from the treatment of ACS, disability adjusted life years (DALYs) avoided will be estimated based on improvements in numbers of patients treated after ACS and improvements in the numbers of patients treated within the first 90 minutes of symptom onset. This outcome will be estimated using information from the published literature and project data.

One-way and multivariable probabilistic sensitivity analyses will be conducted whereby model inputs are varied in order to test robustness of cost-effectiveness estimates. Where relevant, we will also draw on advice set out for economic modelling in the Australian government Technical Guidelines for preparing assessment reports for the Medical Services Advisory Committee[44] and guidelines for undertaking health economic evaluation alongside stepped wedge trials.[42]

The steering committee will be involved in the decisions made for modelling assumptions and the best sources of evidence for this sub-study where these are unable to be obtained from the study data.

## DATA MANAGEMENT

### Data entry

Research Electronic Data Capture (REDCap) will be used for capturing Heart Matters session (e.g. anonymous surveys and session information) data. REDCap is a secure web application that is widely used for building and managing surveys and online databases. It has an intuitive interface for validated data entry, audit trails for tracking data manipulation and export procedures and allows for seamless downloads of collected data to common statistical packages.

### Security and storage

All data collected externally will be electronically send to Monash University investigators fully de-identified via the Secure File Transfer Protocol (SFTP). All data will be kept on a secure password protected server at Monash University maintaining confidentiality in accordance with local legislation. Access to the data will be restricted to CIA, the study coordinator and statistician.

Monash University implements a defence in depth approach to information security and employs a multitude of controls to protect its infrastructure and data. These controls are regularly audited to ensure they meet global best practices and are aligned with ISO 27001 security practices. Data collected will be stored on University managed secure and resilient infrastructure located in Australia that complies with all applicable data protection and privacy obligations.

### Retention and archiving

The study team will retain all study records in a safe and secure location for a minimum of 7 years after the completion of the study in accordance with Australian and Victorian

legislation. (*The Australian Code, which asks for a minimum of 5 years from publication date and the Victorian Health Records Act of health information for 7 years).*

## ETHICS AND DISSEMINATION

### Ethical conduct of the study

The study will be conducted in accordance will all relevant ethical and regulatory approvals and guidelines set by the National Health and Medical Research Council of Australia, the Declaration of Helsinki, its subsequent amendments, and the ICH-GCP guidelines on the ethical conduct of research. A Human Research Ethics Committee registered with the NHMRC will review the protocol prior to commencement of the study.

### Protocol amendments

The steering committee will review and approve all protocol amendments. The lead HREC will review any protocol amendments prior to implementing any protocol changes.

### Consent

*Waiver of consent*

A waiver of informed consent is requested from the overseeing HREC to deliver the heart health education campaign and use de-identified data sets from DHHS (VEMD and VAED), Ambulance Victoria (VACAR and calls to ambulance), Medicare (Heart Health check numbers by LGA) and the Heart Foundation (Heart Watch survey data).

The Heart matters study is a cluster-randomised trial where the intervention is delivered at the cluster level (i.e each Local Government Area). With nearly 800,000 residents across the eight LGA's it is not feasible to obtain informed consent for every individual member of the cluster exposed to the intervention.

The use of de-identified data sets meets the requirements set out in chapter 2.3 of the National statement for a waiver of consent. The use of data sets from DHHS (VEMD and VAED), Ambulance Victoria (VACAR and calls to ambulance), Medicare (Heart Health check numbers by LGA and the Heart Foundation (Heart Watch survey data) is routinely collected and de-identified therefore posing a low risk and it is impractical to obtain consent for data that has already been collected. There is sufficient protection of participant privacy as all data sets will be de-identified and investigators will not attempt to re-identify cases.

*Implied consent*

The anonymised data collected from education evaluation surveys will be collected under implied consent. Completion of the survey will be considered as implied consent.

## Confidentiality

The study team will take every precaution to ensure privacy and confidentiality in accordance with local legislation on privacy and the use of health data. As such, only researchers directly involved in the study will have access to patient data. De-identified data will be entered into the database and only aggregate data will be reported and published.

## Dissemination and authorship

Study protocol, economic and statistical analysis plans will be published in a peer-review journal and prior to trial commencement. The trial will be registered on clinical trials.org.

Trial results will be communicated by publication (e.g. medical journal articles, media releases, website and social media) and presentations (e.g. scientific conferences, stakeholder presentations).

*Authorship eligibility guidelines*

Authorship with be granted to all chief and associate investigators who meet the ICMJE guidelines and maintain a role in the study throughout its duration.

The ICJME recommends that authorship be based on the following four criteria:

1. Substantial contributions to the conception or design of the work; or the acquisition, analysis, or interpretation of the data for the work; AND
2. Drafting the work or revising it critically for important intellectual content; AND
3. Final approval of the version to be published; AND
4. Agreement to be accountable for all aspects of the work in ensuring that questions related to the accuracy or integrity of any part of the work are appropriately investigated and resolved.

Those who contribute to the study but do not meet all four criteria for authorship will be listed in an acknowledgements section within the publication.

## COVID-19 Planning

In March 2020, Victoria was declared a state of emergency due to COVID-19 and in July 2020 a state of disaster was declared. During this time, Victoria has been under stage 3 and stage 4 restrictions where there are only four reasons to leave home; shopping for essentials, medical treatment, work and education (if permitted) and exercise (two person limit). With such restrictions in place, planned Heart Matters interventions such as the launch events and face-to-face sessions with community groups cannot take place. For this reason, the intervention phase has been postponed to April 2021 in the hope that restrictions will have eased by that time. If restrictions are still in place, the intervention will be delivered in a digital format. Where restrictions have eased but social distancing can be applied, the intervention will be adapted to fit these requirement, i.e. small groups outdoors.

## STUDY TIMELINES

| Time period | Actions |
|---|---|
| **Planning Phase**<br>April 2020 –February 2021 | Protocol Development<br>Ethics application<br>Intervention refinement<br>Evaluation refinement<br>Employ staff<br>Gather and develop education materials<br>Gather and develop evaluation materials<br>Publications: background and protocol<br>Publication plan |
| **Intervention Phase**<br>April 2021- July 2022 | Intervention<br>Data collection (hospital and evaluation)<br>Publications: statistical plan and economic evaluation plan |
| **Evaluation Phase**<br>July 2022- December 2023 | Data collection (major datasets) and analysis<br>Publications: main study, sub-studies.<br>Reporting |

## REFERENCES


1. AIHW. Cardiovascular disease snapshot. 2018;Cat. no: CVD 83.
2. AIHW. Australian's Health 2016. 2016;Cat. no: AUS 199. .
3. AIHW. Risk factors to health. 2018.
4. Rohla M, Haberfeld H, Sinzinger H, Kritz H, Tscharre M, Freynhofer MK, et al. Systematic screening for cardiovascular risk at pharmacies. Open Heart. 2016;3(2):e000497.
5. Peterson GM, Fitzmaurice KD, Kruup H, Jackson SL, Rasiah RL. Cardiovascular risk screening program in Australian community pharmacies. Pharm World Sci. 2010;32(3):373-80.
6. Chew DP, Scott IA, Cullen L, French JK, Briffa TG, Tideman PA, et al. National Heart Foundation of Australia and Cardiac Society of Australia and New Zealand: Australian clinical guidelines for the management of acute coronary syndromes 2016. Med J Aust. 2016;205(3):128-33.
7. O'Connor RE, Ali ASA, Brady WJ, Ghaemmaghami CA, Menon V, Welsford M, et al. Part 9: Acute Coronary Syndromes. Circulation. 2015;132(18_suppl_2):S483-S500.
8. Gersh BJ, Stone GW, White HD, Holmes DR, Jr. Pharmacological facilitation of primary percutaneous coronary intervention for acute myocardial infarction: is the slope of the curve the shape of the future? JAMA. 2005;293(8):979-86.
9. Prasad A, Gersh BJ, Mehran R, Brodie BR, Brener SJ, Dizon JM, et al. Effect of Ischemia Duration and Door-to-Balloon Time on Myocardial Perfusion in ST-Segment Elevation Myocardial Infarction: An Analysis From HORIZONS-AMI Trial (Harmonizing Outcomes with Revascularization and Stents in Acute Myocardial Infarction). JACC Cardiovasc Interv. 2015;8(15):1966-74.
10. Guerchicoff A, Brener SJ, Maehara A, Witzenbichler B, Fahy M, Xu K, et al. Impact of delay to reperfusion on reperfusion success, infarct size, and clinical outcomes in patients with ST-segment elevation myocardial infarction: the INFUSE-AMI Trial (INFUSE-Anterior Myocardial Infarction). JACC Cardiovasc Interv. 2014;7(7):733-40.
11. De Luca G, Suryapranata H, Ottervanger JP, Antman EM. Time delay to treatment and mortality in primary angioplasty for acute myocardial infarction: every minute of delay counts. Circulation. 2004;109(10):1223-5.
12. National Heart Foundation of Australia. Australian acute coronary syndromes capability framework. https://wwwheartfoundationorgau/images/uploads/publications/ACS_frameworkpdf. 2015.
13. Bagai A, Jollis JG, Dauerman HL, Peng SA, Rokos IC, Bates ER, et al. Emergency department bypass for ST-Segment-elevation myocardial infarction patients identified with a prehospital electrocardiogram: a report from the American Heart Association Mission: Lifeline program. Circulation. 2013;128(4):352-9.
14. Terkelsen CJ, Sorensen JT, Maeng M, Jensen LO, Tilsted HH, Trautner S, et al. System delay and mortality among patients with STEMI treated with primary percutaneous coronary intervention. JAMA. 2010;304(7):763-71.
15. Morrison LJ, Verbeek PR, McDonald AC, Sawadsky BV, Cook DJ. Mortality and prehospital thrombolysis for acute myocardial infarction: A meta-analysis. JAMA. 2000;283(20):2686-92.

16. Bray JE, Stub D, Ngu P, Cartledge S, Straney L, Stewart M, et al. Mass Media Campaigns' Influence on Prehospital Behavior for Acute Coronary Syndromes: An Evaluation of the Australian Heart Foundation's Warning Signs Campaign. J Am Heart Assoc. 2015;4(7).
17. Müller D, Agrawal R, Arntz H-R. How Sudden Is Sudden Cardiac Death? Circulation. 2006;114(11):1146-50.
18. Leventhal H, Phillips LA, Burns E. The Common-Sense Model of Self-Regulation (CSM): a dynamic framework for understanding illness self-management. J Behav Med. 2016;39(6):935-46.
19. Moser DK, Kimble LP, Alberts MJ, Alonzo A, Croft JB, Dracup K, et al. Reducing delay in seeking treatment by patients with acute coronary syndrome and stroke: a scientific statement from the American Heart Association Council on cardiovascular nursing and stroke council. Circulation. 2006;114(2):168-82.
20. Cartledge S, Finn J, Straney L, Ngu P, Stub D, Patsamanis H, et al. The barriers associated with emergency medical service use for acute coronary syndrome: the awareness and influence of an Australian public mass media campaign. Emerg Med J. 2017;34(7):466-71.
21. Nehme Z, Andrew E, Bernard S, Patsamanis H, Cameron P, Bray JE, et al. Impact of a public awareness campaign on out-of-hospital cardiac arrest incidence and mortality rates. Eur Heart J. 2017;38(21):1666-73.
22. Nehme Z, Cameron PA, Akram M, Patsamanis H, Bray JE, Meredith IT, et al. Effect of a mass media campaign on ambulance use for chest pain. Med J Aust. 2017;206(1):30-5.
23. Bray JE, Straney L, Patsamanis H, Stavreski B, J F. Abstract 20858: Australian Awareness of Heart Attack Symptoms and Action Improves With a Mass Media Warning Signs Campaign. Circulation. 2016;134(suppl_1):A20858.
24. Finn JC, Bett JH, Shilton TR, Cunningham C, Thompson PL, National Heart Foundation of Australia Chest Pain Every Minute Counts Working G. Patient delay in responding to symptoms of possible heart attack: can we reduce time to care? Med J Aust. 2007;187(5):293-8.
25. Thompson PL, Shilton T. Encouraging early treatment of suspected heart attack: it's OK to call 000. Med J Aust. 2017;206(1):19.
26. Bray JE, Finn J, Cameron P, Smith K, Straney L, Cartledge S, et al. Temporal Trends in Emergency Medical Services and General Practitioner Use for Acute Stroke After Australian Public Education Campaigns. Stroke. 2018;49(12):3078-80.
27. Luepker RV, Raczynski JM, Osganian S, Goldberg RJ, Finnegan JR, Jr., Hedges JR, et al. Effect of a community intervention on patient delay and emergency medical service use in acute coronary heart disease: The Rapid Early Action for Coronary Treatment (REACT) Trial. JAMA. 2000;284(1):60-7.
28. Mooney M, McKee G, Fealy G, O'Brien F, O'Donnell S, Moser D. A review of interventions aimed at reducing pre-hospital delay time in acute coronary syndrome: what has worked and why? European journal of cardiovascular nursing : journal of the Working Group on Cardiovascular Nursing of the European Society of Cardiology. 2012;11(4):445-53.
29. Luepker RV, Murray DM, Jacobs DR, Jr., Mittelmark MB, Bracht N, Carlaw R, et al. Community education for cardiovascular disease prevention: risk factor changes in the Minnesota Heart Health Program. Am J Public Health. 1994;84(9):1383-93.

30. Higginbotham N, Heading G, McElduff P, Dobson A, Heller R. Reducing coronary heart disease in the Australian Coalfields: evaluation of a 10-year community intervention. Soc Sci Med. 1999;48(5):683-92.
31. Dracup K, Bryan-Brown CW. Reducing patient delay in seeking treatment. American journal of critical care : an official publication, American Association of Critical-Care Nurses. 1997;6(6):415-7.
32. National Heart Foundation of Australia. Heart Maps. 2018;https://www.heartfoundation.org.au/for-professionals/heart-maps/australian-heart-maps; accessed 02/2019.
33. The Department of Health and Human Services. Victorian Emergency Minimum Dataset [Available from: https://www2.health.vic.gov.au/hospitals-and-health-services/data-reporting/health-data-standards-systems/data-collections/vemd.
34. The Department of Health and Human Services. Victorian Admitted Episodes Dataset [Available from: https://www2.health.vic.gov.au/hospitals-and-health-services/data-reporting/health-data-standards-systems/data-collections/vaed.
35. Kasza J, Hemming K, Hooper R, Matthews J, Forbes AB, Outcomes ACf, et al. Impact of non-uniform correlation structure on sample size and power in multiple-period cluster randomised trials. Stat Methods Med Res. 2019:962280217734981.
36. Hemming K, Girling A. A menu-driven facility for power and detectable-difference calculations in stepped-wedge cluster-randomized trials. . Stata Journal. 2014;14(2):363-80.
37. Hemming K, Taljaard M, McKenzie JE, Hooper R, Copas A, Thompson JA, et al. Reporting of stepped wedge cluster randomised trials: extension of the CONSORT 2010 statement with explanation and elaboration. BMJ. 2018;363:k1614.
38. Kenward MG, Roger JH. Small sample inference for fixed effects from restricted maximum likelihood. Biometrics. 1997;53(3):983-97.
39. Kasza J, Forbes AB. Inference for the treatment effect in multiple-period cluster randomised trials when random effect correlation structure is misspecified. Stat Methods Med Res. 2018:962280218797151.
40. Hoffmann TC, Glasziou PP, Boutron I, Milne R, Perera R, Moher D, et al. Better reporting of interventions: template for intervention description and replication (TIDieR) checklist and guide. BMJ. 2014;348:g1687.
41. Husereau D, Drummond M, Petrou S, Carswell C, Moher D, Greenberg D, et al. Consolidated Health Economic Evaluation Reporting Standards (CHEERS) statement. BMJ : British Medical Journal. 2013;346:f1049.
42. Lung T, Si L, Hooper R, Di Tanna GL. Health Economic Evaluation Alongside Stepped Wedge Trials: A Methodological Systematic Review. PharmacoEconomics. 2020.
43. Cost-Consequence AnalysisCost-consequence analysis. In: Kirch W, editor. Encyclopedia of Public Health. Dordrecht: Springer Netherlands; 2008. p. 168-.
44. Medical Services Advisory Committee. Technical Guidelines for preparing assessment reports for the Medical Services Advisory Committee - Service Type Investigative. In: Department of Health, editor. Canberra ACT2017.

# APPENDICES

Daily Activity reports HM coordinators

- Date
- Location of presentation
- Number of attendees at presentation
- Materials used
- Presentation type
- Any key barriers to this session?
- Any key facilitators to this session?

HEART MATTERS

A joint project, saving lives through community education

Date ______________________

Group/Business ______________________

# Heart Matters Evaluation Form

Completing this form tells us who we reached and how well we shared the Heart Matters message with you. This is a two-sided form.

## About you

**Your postcode:** ..................................... **I live in the Maribyrnong region?** Yes | No

**How do you describe your gender?** Woman | Man | Non-binary | Prefer not to say

**Please circle your age range**

Under 18 | 18-24 | 25-34 | 35-39 | 40-44 | 45-49 | 50-54 | 55-59 | 60-64 | 65-74 | 75+

**In what country were you born?** Australia | Other (please specify) ..............................

**Do you identify as Aboriginal and/or Torres Strait Islander?** Circle *all* that apply

No | Aboriginal | Torres Strait Islander | Prefer not to say

**Have you (or someone close to you) had a heart attack?** Yes | No | I'm not sure

**Have you had a Heart Health Check or something like it?** Yes | No | I'm not sure

---

## Following today's session

| **How confident are you in the following?** | Not at all | A little | Not sure | Somewhat | Yes, definitely |
|---|---|---|---|---|---|
| I could name the heart attack warning signs | 👎👎 | 👎 | 👉 | 👍 | 👍👍 |
| I know what I would do if I thought I was having a heart attack | 👎👎 | 👎 | 👉 | 👍 | 👍👍 |
| I would call 000 if I (or someone else) had heart attack warning signs | 👎👎 | 👎 | 👉 | 👍 | 👍👍 |
| I know what can increase my chances of having heart disease (risk factors). | 👎👎 | 👎 | 👉 | 👍 | 👍👍 |
| I plan to speak with my doctor about a Heart Health Check | 👎👎 | 👎 | 👉 | 👍 | 👍👍 |
| I plan to share what I've learned today with others | 👎👎 | 👎 | 👉 | 👍 | 👍👍 |

Heart Foundation | Ambulance Victoria | MONASH University | VICTORIA State Government

**What have you learned today that has been the most helpful to you?**

**Do you have anything else to share with us about the session?**

## Further information (*optional*)

Please email me links to the information we learned today? ☐

I'm happy for the Heart Matters team to contact me to hear more about my experience of this session. ☐

**Please print clearly**

Your name ____________________

Email address ____________________

Phone number ____________________

Best time to call ____________________

*Thanks for your feedback*

# Heart Matters:

# A cluster randomized controlled trial of heart health education targeting Victorian communities at high risk of Acute Coronary Syndrome

# PROTOCOL

| | |
|---|---|
| **Short title** | Heart Matters |
| **Trial Registration:** | *291276657* |
| **Protocol Version** | V1.1 |
| **Date** | 22/08/2023 |
| **Stage** | FINAL |


| | |
|---|---|
| **Funding** | NHRMC Partnership Grant #1180282 |
| **Funding Partners** | Monash University<br>National Heart Foundation Australia (Heart Foundation)<br>Victorian Department of Health and Human Services (DHHS)<br>Ambulance Victoria (AV) |
| **Partner organisations** | Safer Care Victoria<br>Alfred Health: The Alfred Hospital<br>Bendigo Health<br>Northern Health: The Northern Hospital<br>Western Health: Footscray Hospital<br>Western Health: Sunshine Hospital |


**Protocol Amendments**

| Amendment No. | Date of Amendment | Date of Approval |
|---|---|---|
| 1 | 22/08/2023 | |
| | | |
| | | |

**Protocol Amendments:** 22/8/2023 Personnel, datasets and study timeframe updated. **CONTACTS**

| | |
|---|---|
| Sponsor | Monash University<br>School of Public Health and Preventive Medicine |
| Principal Investigator | Professor Janet Bray<br>Monash University<br>School of Public Health and Preventive Medicine<br>Email: Janet.bray@monash.edu |
| Investigator Group | Prof Judith Finn, Curtin University<br>Prof Robyn Clark, Flinders University<br>Dr Ziad Nehme, Ambulance Victoria<br>Prof Dion Stub, The Alfred and Western Hospitals.<br>Prof Karen Smith, Ambulance Victoria<br>Prof Clara Chow, Westmead Applied Research Centre, University of Sydney<br>Prof Dominique Cadilhac, Monash University and the Florey Institute of Neuroscience and Mental Health<br>A/Prof Jessica Kasza, Monash University<br>Prof Peter Cameron, The Alfred Hospital and Monash University<br>A/Prof Arthur Nasis, Cardiac Clinical Network and Safer Care Victoria<br>Prof Ben Smith, University of Sydney<br>A/Prof Nicholas Cox, Western Health<br>Dr Susie Cartledge, Monash University<br>Prof Tony Walker, Ambulance Victoria<br>Dr Voltaire Nadurata, Bendigo Hospital<br>Prof William Van Gaal, Northern Health<br>Ms Lizzie Flemming, Consumer Advisor<br>Dr Alison Beauchamp, Monash University<br>Dr Joosup Kim, Monash University |
| Project Managers | Ms Natasha Dodge and Ms Janelle Woods, Monash University |

# TABLE OF CONTENTS

LIST OF ABBREVIATIONS ..... 4
SYNOPSIS ..... 5
BACKGROUND AND SIGNIFICANCE ..... 7
AIM ..... 10
OBJECTIVES ..... 11
STUDY DESIGN ..... 11
1. Stepped-wedge cluster randomised trial ..... 12
*Study setting* ..... 12
*Intervention* ..... 12
SW-CRT Outcomes ..... 13
Data collection and data sources ..... 14
Sample size ..... 16
Recruitment ..... 17
Allocation and sequence generation ..... 17
Statistical methods of analyses for the primary and secondary clinical outcomes ..... 18
2. ProCESS Evaluation ..... 18
Process evaluation indicators: ..... 18
3. Cost-effectiveness Evaluation ..... 19
DATA MANAGEMENT ..... 20
Data entry ..... 20
Security and storage ..... 20
Retention and archiving ..... 21
ETHICS AND DISSEMINATION ..... 21
Ethical conduct of the study ..... 21
Protocol amendments ..... 21
Consent ..... 21
Confidentiality ..... 22
Dissemination and authorship ..... 22
COVID-19 Planning ..... 22
STUDY TIMELINES ..... 24
REFERENCES ..... 25
APPENDICES ..... 28

## LIST OF ABBREVIATIONS

| | |
|---|---|
| **ACS** | Acute Coronary Syndrome |
| **AI** | Associate Investigator |
| **AMI** | Acute Myocardial Infarction |
| **AV** | Ambulance Victoria |
| **CAD** | Computer-aided dispatch |
| **CI** | Chief Investigator |
| **DALY** | Disability-Adjusted Life Year |
| **DHHS** | Department of Health and Human Services |
| **ED** | Emergency Department |
| **EMS** | Emergency Medical Services |
| **GP** | General Practitioner |
| **HM** | Heart Matters |
| **HREC** | Human Research Ethics Committee |
| **LGA** | Local Government Area |
| **LHD** | Local Health District |
| **NHFA** | National Heart Foundation Australia |
| **NHMRC** | National Health and Medical Research Council |
| **OHCA** | Out of Hospital Cardiac Arrest |
| **REDCap** | Research Electronic Data Capture |
| **SFTP** | Secure File Transfer Protocol |
| **VACAR** | Victorian Cardiac Arrest Registry |
| **VCOR** | Victorian Cardiac Outcomes Registry |
| **VEMD** | Victorian Emergency Minimum Dataset |
| **VAED** | Victorian Admitted Episodes Dataset |
| **VASQI** | Victorian Ambulance STEMI Quality Initiative |
| **SED** | Socio-economic Disadvantage |
| **SW-CRT** | Stepped-wedge cluster randomised trial |

## SYNOPSIS

| | |
|---|---|
| **Title** | *Heart Matters: A stepped-wedge cluster randomized controlled trial (SW-CRT) of heart health education targeting Victorian communities at high risk of acute coronary syndrome (ACS).* |
| **Aim** | *The study aims to evaluate whether providing a heart health education campaign targeted at communities at highest risk of heart attacks will improve symptom recognition and response.* |
| **Null Hypothesis** | *There will be no change in the proportion of ACS patients arriving via ambulance during the intervention phase.* |
| **Primary objectives** | 1. *Increase ambulance use in ACS patients;*<br>2. *Decrease patient and prehospital delay times in ACS patients;*<br>3. *Increase awareness of personal cardiovascular risk and associated factors in adult community members; and*<br>4. *Increase cardiovascular knowledge and confidence to act to heart attack warning signs in adult community members.* |
| **Intervention** | *A community heart health education campaign that targets regions at high risk of heart attack.* |
| **Study Design** | *A stepped-wedged cluster randomised trial of eight local government areas (LGAs) at highest of heart attacks in Victoria, Australia.* |
| **Outcomes** | ***Primary outcome***<br>- *The proportion of ACS patients that present to emergency department (ED) by ambulance*<br><br>***Secondary outcomes***<br>- *Median patient delay time for ACS/STEMI patients*<br>- *Median prehospital delay times for ACS/STEMI patients*<br>- *The proportion of STEMI patients with patient delay times <60 minutes*<br>- *The proportion of STEMI patients with prehospital delay times <120 minutes*<br>- *The proportion of adult members of the community aware of their own heart attack risk*<br>- *The proportion of adult members of the community aware of heart disease as leading cause of death* |

| | |
|---|---|
| | - *The proportion of adult members of the community aware of cardiovascular risk factors*<br>- *The proportion of adult members of the community aware of heart attack symptoms*<br>- *The number of correctly named ACS symptoms.*<br>- *The proportion of adult members of the community confident to respond to heart attack symptoms*<br>- *The proportion of adult members of the community who would use an ambulance in heart attack scenarios*<br>- *Medicare rates of General Practitioner (GP) Heart Health Checks*<br>- *Rates of ED presentations for ACS and unspecified chest pain*<br>- *Incidence of OHCA*<br>- *The proportion of OHCA and ACS survival to hospital discharge*<br>- *The proportion of OHCA survival to hospital discharge*<br>- *The proportion of ACS survival to hospital discharge*<br>- *Rates of calls to ambulance for chest pain*<br>- *Rates of calls to ambulance for non-chest pain*<br>- *The cost effectiveness of the intervention relative to the control periods*<br><br>***Process outcomes***<br>- *Acceptability of education by participants attending sessions*<br>- *Participation rates in education sessions*<br>- *The proportion of adult members of the community exposed to the Heart Matters intervention*<br>- *The characteristics of those exposed to the Heart Matters intervention*<br>- *The proportion of ACS patients reporting that education was beneficial for treatment seeking*<br>- *Lessons learnt by Heart Matters coordinators* |
| **Sample Size** | *2240 ACS patients (primary outcome)* |
| **Population** | *Eight LGA's in Victoria, Australia selected from 16 LGA's with highest ranking for age-standardised rates of heart attacks.* |
| **Study Duration** | *48 months* |

# BACKGROUND AND SIGNIFICANCE

## Acute Coronary Syndrome (ACS) is a leading cause of death in Australia

ACS, of which the most severe form is a heart attack, is usually caused by coronary heart disease –the leading cause of death in Australia.[1] An estimated 105,000 Australians are hospitalised with ACS every year.[2] *However, ACS is preventable and treatable –if detected and responded to early.*

## Benefits of community cardiovascular risk assessments

ACS is associated with modifiable risk factors that are now common in the Australian community (e.g. high blood pressure, high blood cholesterol, smoking, diabetes, overweight/obesity, and physical inactivity).[3] Community risk screening interventions typically involve assessing members with no known ACS and referring patients identified “at potential risk” to their general practitioners (GPs) for further screening and management. Published studies of public screenings have found high rates of new identified risk factors (in as many as 66% of people screened), and is associated with improved outcomes if subsequently treated.[4, 5] Public screening and referral to GPs are now supported within the Australian health care system, with the Federal Government’s recent implementation of a Medicare item for GPs to conduct Heart Health Checks in adults aged over 45 years (35 years for Indigenous Australians).

## Benefits of early treatment and ambulance use for ACS

International guidelines strongly recommend emergency treatment for ACS, which include medication (aspirin and thrombolysis) and procedures (revascularisation) to reopen the blocked blood vessels in the heart (collectively referred to as ‘reperfusion therapies’).[6, 7]

*Figure 1. The relationship between time and ACS outcomes.*[8]

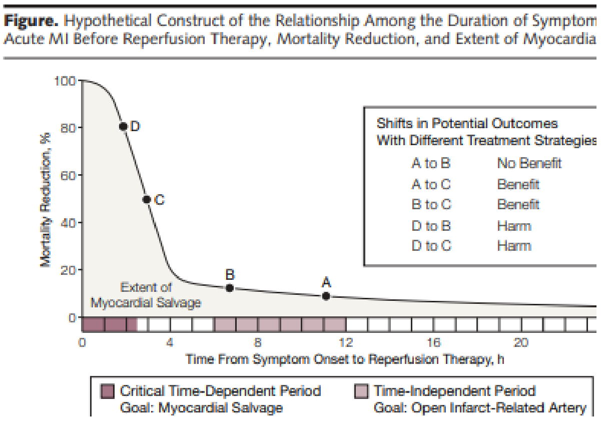


The benefits of reperfusion therapy are maximised when they are administered *within the first 90 minutes following symptom onset (Figure 1)*[8] –these include less heart damage, fewer complications and better short and long-term outcomes.[9-11] Reperfusion therapies are not available at every hospital, which is one of the reasons why ambulance use for ACS is recommended internationally.[7]

Paramedics are trained to detect and provide emergency treatment for ACS, and for the potentially life-threatening complications associated with this condition (e.g. cardiac arrest).[12] Transport by ambulance also ensures suspected ACS patients present to hospitals with revascularisation capabilities, and some ambulance services even initiate thrombolysis during transport.[12] Ambulance use is associated with higher rates of reperfusion therapy, the timeliness of its administration, and improved patient outcomes.[13-15]

**Known factors associated with patient delays and barriers to accessing ambulance care**
To receive optimal outcomes, a person suffering ACS symptoms needs to quickly recognise their symptoms and immediately call an ambulance to receive timely emergency care and ensure transport to a hospital offering revascularisation.[16] *Unfortunately, few ACS patients present to hospital rapidly or by ambulance. Research conducted by CIs/AIs show only 48% of ACS patients recognise their symptoms as heart related, 45% immediately call an ambulance, and only 34% arrive at hospital with two hours of symptom onset.*[16] This work also found that *62% of ACS patients stated their experience was different to what they expected a heart attack to be like.*[16] In addition, the majority of ACS deaths occur in the community, usually before the activation of the emergency system and after hours of typical ACS warning symptoms.[17]

Research driven by illness behaviour theory[18] has uncovered many patient factors that are associated with longer ACS patient delay and failure to use an ambulance.[16, 19, 20] This research suggests major barriers are poor recognition of symptoms as being heart-related, seeking alternate health advice from family members and GPs, and not considering their symptoms serious enough to warrant ambulance care.[3, 20] All factors which are amendable to education.

**Australian campaigns**
The Heart Foundation has previously used public mass media advertisements to increase the Australian public's awareness of ACS symptoms and the need for an urgent response. Evaluations of such campaigns (e.g. the Warning Signs campaign), led by CI/AIs, have shown significant success with associated improvements seen in the public's symptom knowledge, response to symptoms (timeliness and ambulance use) and in prehospital deaths.[16, 21-23]

However, mass media campaigns are expensive, not sustainable and the long-term gains are unknown.[24, 25] For example, the Warning Signs campaign cost an estimated $16 million, ran intermittently between 2009 and 2013, and, as funding was reliant on donations and local government contributions, had variable exposure across the country. Just over half (55%) of Australians[23] and two-thirds (64%) of ACS patients[16] recalled seeing the advertising during the campaign period. The long-term effectiveness of this campaign is unknown, but available data from other campaigns suggest regular and sustained advertising is necessary.[26] There are also concerns about the potential for increased use of emergency services by patients without ACS (i.e. the "worried well") as a result of such campaigns.[25] Data from the Warning Signs campaign suggest this number may be significant.[22] This is an important consideration as emergency services are finite resources, and attending to high numbers of non-ACS patients may well divert resources away from the people with medical emergencies.

**Previous research**
The largest targeted education study to date, the REACT trial, ran an 18-month community and patient education intervention (mass and small media, community and patient group education) in 20 USA communities.[27] While REACT showed a significant increase in ACS symptom knowledge and ambulance use, there was no impact on prehospital delay times.[27]

However, the authors acknowledged that prehospital delay was already low at baseline in their targeted communities.

There have been numerous reviews of ACS education campaigns and interventions which have shown varied impact on patient delay and ambulance use.[24, 28] There is significant variation seen across this body of evidence, including different: health care settings;[16, 21, 22, 28] interventions and exposure; baseline values of outcomes; and the timing and methods of data collection.[24, 28] *Importantly,* most of these studies have used total *prehospital delay* (e.g. time from symptom onset to hospital arrival) as the primary outcome. However, illness behaviour theory suggest a more accurate measure of patient response is *patient delay* – which is the time from symptoms onset to the decision to seek medical attention (e.g. calling an ambulance). It has also been suggested by leading investigators of large community trials that interventions are best carried out in *"populations where risk and rates are high or highest, and where the health promotion message has not penetrated, that is, where knowledge, sophistication, and rapid change in health behaviours have not arrived."*[27, 29]

Education targeting regions at highest risk of ACS may be a more efficient and cost-effective approach, but is understudied internationally. A study conducted in the early 80's targeted a small high-risk ACS rural region without "a clear pattern of improvement".[30] However, this study was conducted without the wealth of knowledge on ACS patient delay [31] and illness behaviour theory;[18] which enables the development of an evidence-based, effective intervention.[19, 24] The identification of regions at highest risk of ACS in Australia is now available as a result of the Heart Foundation's Heart Maps.[32]

**Heart Maps**

The Heart Maps[32] were developed to identify the variation in heart disease across Australia and to highlight areas in greatest need of interventions. In Victoria, these maps include data collected by the DHHS and Ambulance Victoria, and map rates of ACS incidence, mortality, cardiac arrest and risk factors by local government areas (LGAs). Figure 2 shows the Victorian LGAs at lowest (pale yellow) and highest (brown) risk of heart attacks.

*Figure 2. Victorian Heart Map*

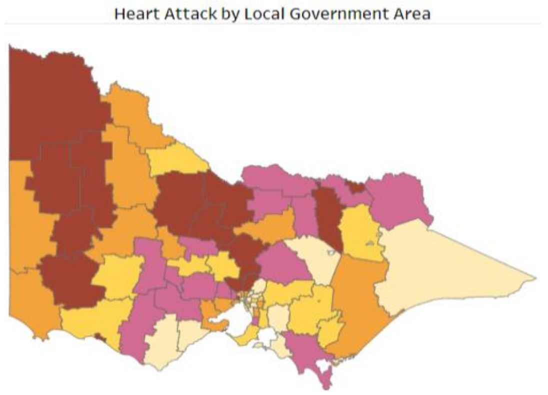


**Formative and Preliminary Heart Matters research**

In developing our proposal, the HM Partners examined some of the underlying characteristics of Victorian high- and low-risk populations. Using available data sources, we compared the 16 LGAs at highest risk of heart attacks to the 16 LGA's at lowest risk. There was no difference in the sex or age distributions of the respective populations; however, residents of high-risk LGAs were more likely to be born in Australia (particularly rural LGAs) and have lower levels of education and socio-economic status (data from ABS). Compared to low-risk regions, high-risk LGAs also had:

- Higher rates for heart failure, out-of-hospital cardiac arrest (OHCA), and cardiovascular mortality (Heart Foundation Heart Maps).

- Less cardiovascular knowledge: risk factors (e.g. poor diet: 50% vs 56%; smoking: 19% vs. 23%; difference between a heart attack and a cardiac arrest: 23% vs. 43%, Heart Foundation Heart Watch).
- Higher self-reported ACS risk factors: hypertension and obesity, lower fruit and vegetable intake, and poorer overall health (DHHS Health survey).
- Lower rates of ambulance use for ACS: Odds Ratio 0.86 (95% CI: 0.83-0.91, $p<0.001$) after adjusting for differences in baseline characteristics known to predict ambulance use (DHHS).

*Targeting these high-risk regions with a **community-based heart health education** seems a more logical and efficient use of funding and resources than large mass media campaigns.*

## AIM

The aim of the study is to evaluate whether providing a targeted heart health education campaign to 8 LGAs identified at high risk of heart attacks will improve ACS patient's symptom recognition and response in these regions. An overview of the Heart Matters Partnership is given in Figure 3.

Figure 3: Overview of the Heart Matters Partnership.

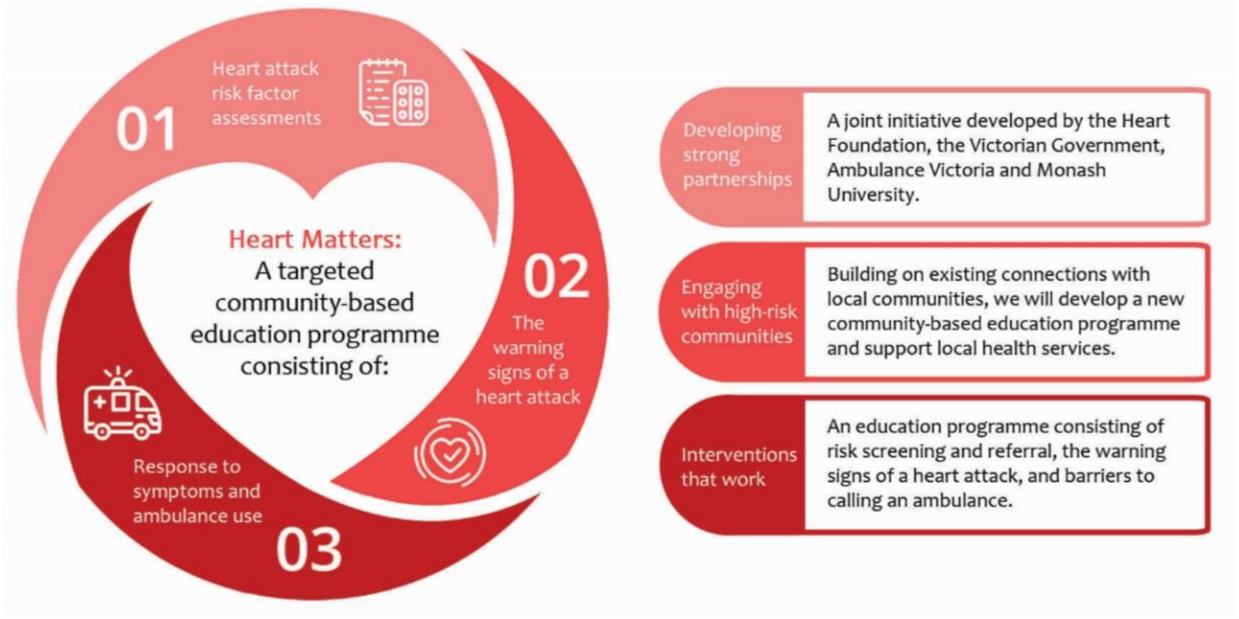

## OBJECTIVES

### Primary objectives

To determine if targeted heart health education will:

1. Increase ambulance use in ACS patients;
2. Decrease patient and prehospital delay times in ACS patients;
3. Increase awareness of personal cardiovascular risk and associated factors in adult community members; and
4. Increase cardiovascular knowledge and confidence to act to heart attack warning signs in adult community members.

### Secondary objectives

1. To determine if targeted heart health education:
    - Reduces the incidence of out-of-hospital cardiac arrest;
    - Improves survival in OHCA patients;
    - Improves survival in ACS patients;
    - Improves survival and ACS patients;
    - Increases presentations to ED for ACS and unspecified chest pain; and
    - Increases the rates of calls to ambulance for chest pain and non-chest pain.
    - Increases the rate of Heart Health Checks.

2. To evaluate the implementation of the intervention by determining:
    - Participation rates in education and screening;
    - Reach of the intervention to community members;
    - Barriers and enablers for heart health educators/coordinators;
    - Barriers and enablers for community members;
    - Levels of exposure to the Heart Matters intervention;
    - Demographics of those who engaged with the Heart Matters intervention;
    - Reported use of the Heart Matters education messages in relation to pre-hospital delay and behaviour; and

3. To determine the cost effectiveness of the targeted intervention by completing an economic evaluation

## STUDY DESIGN

The main study will use a stepped-wedge cluster randomised trial (SW-CRT) to evaluate the effectiveness of the intervention. Concurrently we will also conduct a process and economic evaluation.

## 1. STEPPED-WEDGE CLUSTER RANDOMISED TRIAL

The stepped-wedge design is a uni-directional cross-over design – where the randomisation element is when the cluster crosses-over to the intervention following a control period.

*Study setting*

The study will be undertaken in eight LGAs in Victoria (3 in greater Melbourne and 5 in regional Victoria.

These LGAs were selected from the list of the 16 highest-risk Victorian LGAs (highest ranking for age-standardised rates of heart attacks) generated from the Victorian Heart Maps.[32] These LGAs were selected to meet the study's sample size requirements based on admission rates for ACS from the Victorian Emergency Medicine Dataset (VEMD). Estimates, from the 2013-2015 VEMD data, suggests 1,635 ACS events occur in these LGAs annually (annual range across LGAs=102 to 360).

*Table 1. Profiles of the 8 LGAs to be targeted (*SED= Index of Relative Socio-economic Disadvantage).*

| LGA name | Area km2 | Residents | Index SED* | Median age | Males (%) | Born overseas (%) |
|---|---|---|---|---|---|---|
| LGA1 | 489 | 207058 | 991 | 34 | 50 | 36% |
| LGA2 | 503 | 207041 | 947 | 33 | 50 | 36% |
| LGA3 | 31 | 86942 | 995 | 34 | 51 | 40% |
| LGA4 | 2999 | 112267 | 981 | 38 | 49 | 8% |
| LGA5 | 22082 | 54658 | 935 | 40 | 50 | 11% |
| LGA6 | 432 | 40100 | 977 | 36 | 50 | 10% |
| LGA7 | 4518 | 37595 | 967 | 45 | 50 | 7% |
| LGA8 | 120.7 | 33655 | 986 | 40 | 50 | 8% |

*Intervention*

To meet the objectives of the study, the Heart Foundation will employ HM coordinators for each of the eight high-risk LGAs to organise and deliver our HM education program using HM materials and Partner resources.

*Heart Matters Coordinators*

HM coordinators will be appropriately qualified (e.g. nursing, health promotion) and based in each LGA at a local ambulance branch. They will undergo HM training by investigators to provide and coordinate risk screening and education sessions. They will complete daily activity reports online and be supervised by CIA Bray, the HM Project Manager and Heart Foundation appointed staff. Coordinators will review activities weekly with the HM Project Manager to ensure adherence to protocol.

*Education materials and resources*

Once an LGA crosses over into the intervention phase, a launch event will be held and the HM coordinator will start delivering community education (Table 2). This education will address each of the study's objectives using varying modes of delivery (e.g. in person* and digital). Partner resources will be used (e.g. Heart Foundation's Heart Attack Action Plan – available in multiple languages and magnetic to keep on fridge). HM resources used by HM Coordinators (e.g. videos and presentations) will be developed from existing resources and tested with Partners and collaborators.

* All education will be online (e.g. Zoom webinars) if Government restrictions apply due to the COVID pandemic.

*Table 2. Proposed methods of HM education.*

| |
|---|
| HM coordinator education:<br>• Face-to-face/digital "Know your risk" screening and information sessions.<br>• Face-to-face/digital heart health education sessions with community groups (e.g. Seniors Clubs, Church groups, Lions Rotary, Country Women's Associations, Men's Sheds, Neighbourhood/Community Houses).<br>• Face-to-face/digital targeted education (e.g. face-to-face/digital education seminars for ACS patients and families).<br>• Webinar education sessions after-hours for public and patients.<br>• Nurse Cora -Avatar teaching App[3]: an 8 minute evidence based educational app designed for low health literacy and when English as a second language. The Avatar can also be electronically programmed to speak 144 languages. Features quizzes to engage learners to test knowledge and response to ACS symptoms. |
| Regional events:<br>• Launch event –involving all Partners, local health care professionals, local heart attack survivors and media.<br>• Local media advertisements of sessions and heart health education (e.g. local magazines).<br>• Website – resources, events, programs and frequently asked Q&A.<br>• Local promotion of existing programs –e.g. Walking Program.<br>• Geo-targeted social media advertising with Heart Foundation's Heart Age Calculator and Warning Signs.<br>• Web banner advertising on local organisations/business sites. |

HM coordinators will use local and Partner resources (e.g. speakers) as needed:

- Heart Foundation: Regional Coordinators; Health Professional Volunteers Register; Heart Foundation funded Scholars and Fellows; heart attack survivors; printed and electronic resources; and existing programs.
- Ambulance Victoria: regional Paramedic Community Support Coordinators; and paramedics on light-duties.
- Local: Cardiology/emergency health professionals; medical/nursing/paramedic students.

## SW-CRT Outcomes

The primary and secondary clinical outcomes as listed in Table 3 will be provided to the investigators by the data sources for all Victorian LGAs over the 16-month study period and for one-year after the intervention period to examine the longer-term impact. Data for the whole of Victoria is required to enable a secondary analysis of non-study LGAs. The main primary outcome is the proportion of ACS patients that present to ED by ambulance.

Data collection and data sources

All outcome data are already currently being collected by the data sources listed in Table 3 and described below. Data are required for the entire 16-month study period (main study) and for 12-months after (sustained effect). All data will be provided to Monash University in a de-identified format. Data will be sent to Monash University electronically via a secure file transfer. Data will be provided under a waiver of consent (see ethics below).

*Table 3. Study Outcomes*

| Primary Outcome | Data Sources/Agency |
|---|---|
| The proportion of ACS patients that present to ED by ambulance. | Victorian Emergency Minimum Dataset (VEMD)/ Department of Health and Human Services (DHHS) |
| Secondary outcomes | |
| The median ACS (STEMI) patient delay time (time from symptom onset to decision to seek medical attention). | The Victorian Ambulance STEMI Quality Initiative (VASQI)/ Ambulance Victoria<br>The Victorian Cardiac Outcomes Registry (VCOR)/Monash University |
| The proportion of ACS (STEMI) patients with patient delay times <60 minutes | VASQI/Ambulance Victoria<br>VCOR/Monash University |
| | |
| The median ACS/STEMI patient prehospital delay time (time from symptom onset to arrival at hospital). | VASQI/Ambulance Victoria<br>VCOR/Monash University |
| The proportion of ACS/STEMI patients with prehospital delay times <120 minutes | VASQI/Ambulance Victoria<br>VCOR/Monash University |
| The proportion of adult members of the community who are aware of their own risk of heart attack. | Heart Watch Public Survey/Heart Foundation |
| The proportion of adult members of the community who identify heart disease a leading cause of death | Heart Watch Survey/Heart Foundation |
| The proportion of adult members of the community who identify cardiovascular risk factors | Heart Watch Survey/Heart Foundation |
| The number of correctly named cardiovascular risk factors by adult members of the community | Heart Watch Public Survey/Heart Foundation |
| The proportion of adult members of the community aware of any heart attack symptoms | Heart Watch Public Survey/Heart Foundation |
| The number of correctly named ACS symptoms by adult members of the community | Heart Watch Public Survey/Heart Foundation |
| The proportion adult members of the community who are confident about what they would do if experiencing a heart attack | Heart Watch Survey/Heart Foundation |
| The proportion adult members of the community who correctly state they would call an ambulance for two heart attack scenarios. | Heart Watch Survey/Heart Foundation |
| Rates of Medicare claims for General Practitioner (GP) Heart Health Checks | Medicare data/Medicare |
| Rates of ED presentations that are ACS | VEMD/DHHS |

| | |
|---|---|
| Rates of ED presentations that are unspecified chest pain | VEMD/DHHS |
| Proportion of ACS ED presentations via General Practitioners | VEMD/DHHS |
| Rates of ACS survival | Victorian Admitted Episodes Dataset (VAED)/DHHS |
| Rates and incidence of out-of-hospital cardiac arrest | Victorian Cardiac Arrest registry (VACAR)/Ambulance Victoria |
| Rates out-of-hospital cardiac arrest survival | VACAR/Ambulance Victoria |
| Proportion of chest pain (event type 10) calls to ambulance | Electronic computer-aided dispatch (CAD) records/ Ambulance Victoria |
| Proportion of non-chest pain emergency calls to ambulance | Electronic computer-aided dispatch (CAD) records/Ambulance Victoria |

**The Victorian Emergency Minimum Dataset (VEMD)** is a state-wide emergency dataset collected for all presentations to public emergency departments and maintained by the Victorian Government's Department of Health and Human Services. Currently, VEMD data, which is collected by ED staff (including clerical and health professionals), includes demographic (e.g. age, sex, usual place of residence), administrative (e.g. triage code, destination from ED) and clinical data (e.g. primary diagnosis). This dataset is used for epidemiological purposes, health services planning and coordination; policy assessment and formulation; and clinical research and quality improvement.[33]

**The Victorian Admitted Episodes Dataset (VAED)** comprises demographic, clinical and administrative details for admitted episodes of care occurring in Victorian hospitals, rehabilitation centres, extended care facilities and day procedure centres. VAED data is used to provide equitable funding to public hospitals under the casemix system, support health services planning, policy formulation and epidemiological research, and meet national data reporting requirements.[34]

**The Victorian Cardiac Arrest Registry (VACAR)** records details of all OHCA events where EMSs are in attendance. In-field treatment data are captured electronically using computer tablets operated by paramedics. A highly sensitive search filter is used to identify potential cardiac arrest cases from a database of electronic patient care records. Potentially eligible cases undergo manual review by registry personnel, and cases meeting eligibility criteria are entered into the registry extracting over 150 data points including the Utstein-style descriptors. Complete case ascertainment is assured through the review of computer-aided dispatch records, emergency call logs, and paper-based treatment records. Paramedics are also required to report cardiac arrest cases and submit electrocardiogram recordings.[21]

**The Victorian Ambulance STEMI Quality Initiative (VASQI)** is a clinical quality registry that aims to facilitate quality improvement by collecting and monitoring data regarding the treatment and outcome of paramedic suspected STEMI cases. The VASQI incorporates both

pre-hospital clinical and operational data, and hospital follow-up data. Paramedic suspected STEMI cases are housed in the VASQI with data being drawn from Ambulance Victoria's Communication centre dispatch records, Emergency Medical Services (EMS) patient care records, and from Team Manager review of STEMI cases. Hospital diagnosis, treatment and outcome data for paramedic suspected STEMI cases identified in VASQI are linked from the Victorian Cardiac Outcomes Regsitry (VCOR) at Monash University and by collaboration with Victorian public hospitals with the capability of performing percutaneous coronary intervention (PCI).

**The Victorian Cardiac Outcomes Registry (VCOR)** is a clinical quality registry that aims to improve the quality of care provided to patients with cardiovascular disease. VCOR is coordinated by Monash University and collects data for all Victorian patients undergoing relevant cardiac procedures. The VCOR collects prehospital, hospital and outcome data.

**Ambulance Victoria keep Electronic Computer Aided Dispatch (CAD)** records for all emergency calls to ambulance. This dataset includes date and chief complaint (e.g. chest pain).

**Medicare** provides access to medical and hospital services for all Australian residents and certain categories of visitors to Australia. The Medicare Benefits Schedule (MBS) list services that are subsidised by the Australian Government under Medicare. Reports can be collated providing anonymised demographic variables on Items in the MBS Schedule. Medicare Items 699 and 177, will support patients to access a heart health assessment through a doctor in a general practice. Reports will be collated on use of these item numbers across local government areas during the intervention period.

**The Heart Foundation's "Heart Watch" Survey** is a quarterly survey conducted using a purposive, non-probability sampling method with quotas for age, gender and area of residence in order to reflect the characteristics of the wider Australian population. Respondents of the survey belong to an online survey panel.[20]

## Sample size

The stepped wedge design in the schematic (a six-sequence stepped wedge design, with an implementation period and two clusters assigned to the first and last sequences) will give 80% power to detect a change in the proportion of ACS patients in the eight LGAs arriving via ambulance by 11%, from 61% to 72%, with a two-sided significance level of 5%. This detectable difference depends on the number of patients expected to be observed in each cluster (average of 40 patients per LGA in each two-month period; a total of 2240 patients) and on the intra-cluster correlation, estimated to be 0.09 using data from the VEMD (2011-2015). Examination of these aforementioned data indicated that more complex correlation structures, with decaying correlations over time, were not necessary.[35] We used the Stepped Wedge Stata Program for the sample size calculation.[36] No adjustment was made for LGA or participant attrition due to the outcomes being those routinely collected by health services.

## Recruitment

*Engagement strategies with communities*

Successfully used methods, by the Heart Foundation, will be employed to engage with local communities, including forming local HM Coalitions. Prior to commencement of the study, the CIA, the Project Manager, Partner Investigators and local collaborators (AIs) will set up coalitions in each LGA to engage local stakeholders. We will approach Local Government, community leaders and health service providers (Hospitals, Cardiac Rehabilitation Centres, General Practice Networks, Pharmacies) in each LGA. We will identify possible community groups, local events, promotion opportunities and other opportunities to assist with the intervention delivery. We will advertise HM activities locally (e.g. in newspapers) and on a dedicated HM website.

## Allocation and sequence generation

Over the 16-month study period, the eight LGAs will move into the intervention phase at two month intervals. As four LGAs are in close proximity (Whittlesea-Hume; Greater Bendigo-Campaspe), these LGAs will switch from control to intervention periods at the same time to avoid possible contamination. For planning purposes, allocation and sequence generation has already been performed. Allocation of randomisation was performed blinded, by the CIA, study statistician and overseen by an independent investigator. This was performed by CIA allocating each LGA a study number from 1 to 8, which was then sent to the independent investigator. Following this the study statistician generating the randomisation sequence with the numbers 1 to 8, this information was then linked back to the LGA allocation numbers (Table 4).

*Table 4. Allocation and sequence generation.*

| | Time Period | | | | | | | |
|---|---|---|---|---|---|---|---|---|
| LGAs | T1 | T2 | T3 | T4 | T5 | T6 | T7 | T8 |
| Bendigo | | X | | | | | | |
| Campaspe | | X | | | | | | |
| Maribyrnong | | | X | | | | | |
| Wodonga | | | | X | | | | |
| Warrnambool | | | | | X | | | |
| Mildura | | | | | | X | | |
| Whittlesea | | | | | | | X | |
| Hume | | | | | | | X | |

*X = Launch Event*

*T = time period, two months in duration*

*Proposed timeline for intervention. Light grey cells – control period; medium-grey cells- transition period; dark grey cells – intervention period.*

### Statistical methods of analyses for the primary and secondary clinical outcomes

Results from the trial will be reported according to the CONSORT extension for stepped wedge cluster randomised designs.[37] LGA and patient demographics will be described by LGA and period. All available primary and secondary outcomes will be analysed at the patient level using mixed-effects logistic regression models with a random intercept for cluster and fixed effects for each period and for the intervention. The Kenward-Roger correction will be applied to adjust for the small number of LGAs.[38] Since there may be imbalance between the LGAs with respect to various baseline characteristics, in a secondary analysis we will adjust for potential confounders known to influence ambulance use that are available in the VEMD (e.g. age, sex, Australian born). Estimated effects will be expressed as odds ratios and as risk differences, with 95% confidence intervals. A secondary analysis will include an interaction between time and treatment to investigate a cumulative effect of the intervention over time. It has been shown that misspecification of the within-cluster correlation structure can lead to confidence intervals of incorrect widths,[39] so sensitivity analyses allowing for more complex within-cluster correlation structures[35] will be considered. Analyses will be conducted in Stata and/or SAS as appropriate.

We will also perform subgroup analyses (e.g. age, sex, country of birth) across the primary and secondary outcomes.

## 2. PROCESS EVALUATION

The process evaluation aims to determine whether program activities have been implemented as intended and to provide details of the intervention of allow their replication. This will use mixed methods including online reports, qualitative interviews, and evaluation surveys and will be reported according the TiDier Checklist.[40]

### Process evaluation indicators:

- Participation rates in education and screening: Participation rates in education and screening: HM coordinators will complete an online report for every session – recording number of participants, duration of event, mode of delivery, location, resources used, and content. Heart Foundation will collect website hits and use of online resources (e.g. Heart Age Calculator).
- Lessons learnt: HM coordinators will keep a diary to document barriers and facilitators and we will conduct qualitative interviews with HM coordinators at the end of the study to elucidate key themes.
- Acceptability to individuals: we will conduct brief de-identified session evaluation surveys at the end of every session.
- Reach: ACS patients and Heart Watch participants for the intervention LGAs will be asked if they had participated in any Heart Matter education events, seen and made use of intervention materials.
- Adoption and sustainability by organisations: we will survey partner organisation one year after intervention concludes to see if they have adopted any methods used in this study.

## 3. COST-EFFECTIVENESS EVALUATION

The economic evaluation is conducted as a sub-study to the step wedge cluster randomised controlled trial. The perspective of the economic evaluation is the health care sector and the reference year for costs will be 2022.

A cost consequences analysis and a simulated cost-effectiveness analysis will be undertaken for the economic evaluation of the Heart Matters campaign.

The economic evaluation will be written up based on the consolidated health economics reporting standards[41] and any relevant checklists for reporting economic evaluations of trials using a randomised cluster stepped wedge design. For example, Lung and colleagues have recommended that researchers account for the following in their statistical analyses: correlation between costs and outcomes (individually and within clusters) and in the estimation of incremental cost-effectiveness, the need to make appropriate assumptions about the distribution of outcomes and costs, and adjusting for time-effects (including interactions with intervention effectiveness).[42] These steps are necessary since accounting for secular and within-cluster trends is an additional complexity that needs to be properly accounted for in a stepped-wedged design.[42]

***Costs:***

The costs of providing the program (HM coordinators, education packages) will be described. Health sector costs estimated will include those related to the use of ambulances, hospital emergency presentations and admissions as well as the Medicare rebates for Heart Health Checks. Cost-offsets are the costs of disability, deaths or future events avoided and will be estimated based on published cost-of-illness studies or best available evidence that is relevant to the Victorian context.

Costs will be adjusted to the same reference year because of the 16-month study period.

***Cost consequences analysis:***

Cost consequences analysis will be used to describe the expected costs and benefits from the Heart Matters campaign, including community cardiovascular risk assessments, survival, out-of-hospital cardiac arrest incidence, and cost offsets.

The steering committee for the Heart Matters campaign will be asked to define the most relevant clinical, process and economic outcomes to be reported in the cost consequences analysis.

A cost consequences analysis is preferred given the nature of the intervention and the type of data being used to determine the primary and secondary clinical and process outcomes. In this type of economic analysis all the relevant health and non-health effects across different sectors can be reported.[43] This is useful when different outcomes cannot be incorporated into an index measure and there are several important outcomes of relevance to decision-makers enabling these to be assessed transparently against the costs of an intervention.

***Simulated cost effectiveness analysis:***

The cost-effectiveness of the Heart Matters campaign will be assessed using a simulation model. Health sector costs and disability adjusted life years (DALYs) avoided before and after the delivery of the intervention will be estimated. If appropriate, the incremental cost per DALY avoided from the Heart Matters campaign compared to usual care will be estimated.

Where estimates of resource use are derived from the study, these estimates will be obtained by using mixed-effects logistic regression analysis consistent with that conducted for the primary and secondary outcomes of this study.

Since the primary health benefit is expected to be from the treatment of ACS, disability adjusted life years (DALYs) avoided will be estimated based on improvements in numbers of patients treated after ACS and improvements in the numbers of patients treated within the first 90 minutes of symptom onset. This outcome will be estimated using information from the published literature and project data.

One-way and multivariable probabilistic sensitivity analyses will be conducted whereby model inputs are varied in order to test robustness of cost-effectiveness estimates. Where relevant, we will also draw on advice set out for economic modelling in the Australian government Technical Guidelines for preparing assessment reports for the Medical Services Advisory Committee[44] and guidelines for undertaking health economic evaluation alongside stepped wedge trials.[42]

The steering committee will be involved in the decisions made for modelling assumptions and the best sources of evidence for this sub-study where these are unable to be obtained from the study data.

## DATA MANAGEMENT

### Data entry

Research Electronic Data Capture (REDCap) will be used for capturing Heart Matters session (e.g. anonymous surveys and session information) data. REDCap is a secure web application that is widely used for building and managing surveys and online databases. It has an intuitive interface for validated data entry, audit trails for tracking data manipulation and export procedures and allows for seamless downloads of collected data to common statistical packages.

### Security and storage

All data collected externally will be de-idenified by data custodians and electronically send to Monash University investigators fully de-identified via the Secure File Transfer Protocol (SFTP). VCOR data will be accessed by the statistician for analysis via the VCOR Secure eResearch Platform (SeRP). All data will be kept on a secure password protected server at Monash University maintaining confidentiality in accordance with local legislation. Access to the data will be restricted to CIA, the study coordinator and statistician.

Monash University implements a defence in depth approach to information security and employs a multitude of controls to protect its infrastructure and data. These controls are

regularly audited to ensure they meet global best practices and are aligned with ISO 27001 security practices. Data collected will be stored on University managed secure and resilient infrastructure located in Australia that complies with all applicable data protection and privacy obligations.

### Retention and archiving

The study team will retain all study records in a safe and secure location for a minimum of 7 years after the completion of the study in accordance with Australian and Victorian legislation. (*The Australian Code, which asks for a minimum of 5 years from publication date and the Victorian Health Records Act of health information for 7 years).*

## ETHICS AND DISSEMINATION

### Ethical conduct of the study

The study will be conducted in accordance will all relevant ethical and regulatory approvals and guidelines set by the National Health and Medical Research Council of Australia, the Declaration of Helsinki, its subsequent amendments, and the ICH-GCP guidelines on the ethical conduct of research. A Human Research Ethics Committee registered with the NHMRC will review the protocol prior to commencement of the study.

### Protocol amendments

The steering committee will review and approve all protocol amendments. The lead HREC will review any protocol amendments prior to implementing any protocol changes.

### Consent

*Waiver of consent*

A waiver of informed consent is requested from the overseeing HREC to deliver the heart health education campaign and use de-identified data sets from DHHS (VEMD and VAED), Ambulance Victoria (VACAR, VASQI and calls to ambulance), Monash University (VCOR), Medicare (Heart Health check numbers by LGA) and the Heart Foundation (Heart Watch survey data).

The Heart matters study is a cluster-randomised trial where the intervention is delivered at the cluster level (i.e each Local Government Area).  With nearly 1.8 million residents across the eight LGA's it is not feasible to obtain informed consent for every individual member of the cluster exposed to the intervention.

The use of de-identified data sets meets the requirements set out in chapter 2.3 of the National statement for a waiver of consent. The use of data sets from DHHS (VEMD and VAED), Ambulance Victoria (VACAR, VASQI and calls to ambulance), Monash University (VCOR), Medicare (Heart Health check numbers by LGA and the Heart Foundation (Heart Watch survey data) is routinely collected and de-identified therefore posing a low risk and it

is impractical to obtain consent for data that has already been collected. There is sufficient protection of participant privacy as all data sets will be de-identified and investigators will not attempt to re-identify cases.

*Implied consent*

The anonymised data collected from education evaluation surveys will be collected under implied consent. Completion of the survey will be considered as implied consent.

## Confidentiality

The study team will take every precaution to ensure privacy and confidentiality in accordance with local legislation on privacy and the use of health data. As such, only researchers directly involved in the study will have access to patient data. De-identified data will be entered into the database and only aggregate data will be reported and published.

## Dissemination and authorship

Study protocol, economic and statistical analysis plans will be published in a peer-review journal and prior to trial commencement. The trial will be registered on clinical trials.org.

Trial results will be communicated by publication (e.g. medical journal articles, media releases, website and social media) and presentations (e.g. scientific conferences, stakeholder presentations).

*Authorship eligibility guidelines*

Authorship with be granted to all chief and associate investigators who meet the ICMJE guidelines and maintain a role in the study throughout its duration.

The ICJME recommends that authorship be based on the following four criteria:

1. Substantial contributions to the conception or design of the work; or the acquisition, analysis, or interpretation of the data for the work; AND
2. Drafting the work or revising it critically for important intellectual content; AND
3. Final approval of the version to be published; AND
4. Agreement to be accountable for all aspects of the work in ensuring that questions related to the accuracy or integrity of any part of the work are appropriately investigated and resolved.

Those who contribute to the study but do not meet all four criteria for authorship will be listed in an acknowledgements section within the publication.

## COVID-19 Planning

In March 2020, Victoria was declared a state of emergency due to COVID-19 and in July 2020 a state of disaster was declared. During this time, Victoria has been under stage 3 and stage 4 restrictions where there are only four reasons to leave home; shopping for essentials, medical treatment, work and education (if permitted) and exercise (two person limit). With

such restrictions in place, planned Heart Matters interventions such as the launch events and face-to-face sessions with community groups cannot take place. For this reason, the intervention phase has been postponed to April 2021 in the hope that restrictions will have eased by that time. If restrictions are still in place, the intervention will be delivered in a digital format. Where restrictions have eased but social distancing can be applied, the intervention will be adapted to fit these requirement, i.e. small groups outdoors.

## STUDY TIMELINES

| Time period | Actions |
|---|---|
| **Planning Phase**<br>April 2020 –February 2021 | Protocol Development<br>Ethics application<br>Intervention refinement<br>Evaluation refinement<br>Employ staff<br>Gather and develop education materials<br>Gather and develop evaluation materials<br>Publications: background and protocol<br>Publication plan |
| **Intervention Phase**<br>April 2021- March 2023 | Intervention<br>Data collection (hospital and evaluation)<br>Publications: statistical plan and economic evaluation plan |
| **Evaluation Phase**<br>July 2022- December 2024 | Data collection (major datasets) and analysis<br>Publications: main study, sub-studies.<br>Reporting |

## REFERENCES

1. AIHW. Cardiovascular disease snapshot. 2018;Cat. no: CVD 83.
2. AIHW. Australian's Health 2016. 2016;Cat. no: AUS 199. .
3. AIHW. Risk factors to health. 2018.
4. Rohla M, Haberfeld H, Sinzinger H, Kritz H, Tscharre M, Freynhofer MK, et al. Systematic screening for cardiovascular risk at pharmacies. Open Heart. 2016;3(2):e000497.
5. Peterson GM, Fitzmaurice KD, Kruup H, Jackson SL, Rasiah RL. Cardiovascular risk screening program in Australian community pharmacies. Pharm World Sci. 2010;32(3):373-80.
6. Chew DP, Scott IA, Cullen L, French JK, Briffa TG, Tideman PA, et al. National Heart Foundation of Australia and Cardiac Society of Australia and New Zealand: Australian clinical guidelines for the management of acute coronary syndromes 2016. Med J Aust. 2016;205(3):128-33.
7. O'Connor RE, Ali ASA, Brady WJ, Ghaemmaghami CA, Menon V, Welsford M, et al. Part 9: Acute Coronary Syndromes. Circulation. 2015;132(18_suppl_2):S483-S500.
8. Gersh BJ, Stone GW, White HD, Holmes DR, Jr. Pharmacological facilitation of primary percutaneous coronary intervention for acute myocardial infarction: is the slope of the curve the shape of the future? JAMA. 2005;293(8):979-86.
9. Prasad A, Gersh BJ, Mehran R, Brodie BR, Brener SJ, Dizon JM, et al. Effect of Ischemia Duration and Door-to-Balloon Time on Myocardial Perfusion in ST-Segment Elevation Myocardial Infarction: An Analysis From HORIZONS-AMI Trial (Harmonizing Outcomes with Revascularization and Stents in Acute Myocardial Infarction). JACC Cardiovasc Interv. 2015;8(15):1966-74.
10. Guerchicoff A, Brener SJ, Maehara A, Witzenbichler B, Fahy M, Xu K, et al. Impact of delay to reperfusion on reperfusion success, infarct size, and clinical outcomes in patients with ST-segment elevation myocardial infarction: the INFUSE-AMI Trial (INFUSE-Anterior Myocardial Infarction). JACC Cardiovasc Interv. 2014;7(7):733-40.
11. De Luca G, Suryapranata H, Ottervanger JP, Antman EM. Time delay to treatment and mortality in primary angioplasty for acute myocardial infarction: every minute of delay counts. Circulation. 2004;109(10):1223-5.
12. National Heart Foundation of Australia. Australian acute coronary syndromes capability framework. https://wwwheartfoundationorgau/images/uploads/publications/ACS_frameworkpdf. 2015.
13. Bagai A, Jollis JG, Dauerman HL, Peng SA, Rokos IC, Bates ER, et al. Emergency department bypass for ST-Segment-elevation myocardial infarction patients identified with a prehospital electrocardiogram: a report from the American Heart Association Mission: Lifeline program. Circulation. 2013;128(4):352-9.
14. Terkelsen CJ, Sorensen JT, Maeng M, Jensen LO, Tilsted HH, Trautner S, et al. System delay and mortality among patients with STEMI treated with primary percutaneous coronary intervention. JAMA. 2010;304(7):763-71.
15. Morrison LJ, Verbeek PR, McDonald AC, Sawadsky BV, Cook DJ. Mortality and prehospital thrombolysis for acute myocardial infarction: A meta-analysis. JAMA. 2000;283(20):2686-92.

16. Bray JE, Stub D, Ngu P, Cartledge S, Straney L, Stewart M, et al. Mass Media Campaigns' Influence on Prehospital Behavior for Acute Coronary Syndromes: An Evaluation of the Australian Heart Foundation's Warning Signs Campaign. J Am Heart Assoc. 2015;4(7).
17. Müller D, Agrawal R, Arntz H-R. How Sudden Is Sudden Cardiac Death? Circulation. 2006;114(11):1146-50.
18. Leventhal H, Phillips LA, Burns E. The Common-Sense Model of Self-Regulation (CSM): a dynamic framework for understanding illness self-management. J Behav Med. 2016;39(6):935-46.
19. Moser DK, Kimble LP, Alberts MJ, Alonzo A, Croft JB, Dracup K, et al. Reducing delay in seeking treatment by patients with acute coronary syndrome and stroke: a scientific statement from the American Heart Association Council on cardiovascular nursing and stroke council. Circulation. 2006;114(2):168-82.
20. Cartledge S, Finn J, Straney L, Ngu P, Stub D, Patsamanis H, et al. The barriers associated with emergency medical service use for acute coronary syndrome: the awareness and influence of an Australian public mass media campaign. Emerg Med J. 2017;34(7):466-71.
21. Nehme Z, Andrew E, Bernard S, Patsamanis H, Cameron P, Bray JE, et al. Impact of a public awareness campaign on out-of-hospital cardiac arrest incidence and mortality rates. Eur Heart J. 2017;38(21):1666-73.
22. Nehme Z, Cameron PA, Akram M, Patsamanis H, Bray JE, Meredith IT, et al. Effect of a mass media campaign on ambulance use for chest pain. Med J Aust. 2017;206(1):30-5.
23. Bray JE, Straney L, Patsamanis H, Stavreski B, J F. Abstract 20858: Australian Awareness of Heart Attack Symptoms and Action Improves With a Mass Media Warning Signs Campaign. Circulation. 2016;134(suppl_1):A20858.
24. Finn JC, Bett JH, Shilton TR, Cunningham C, Thompson PL, National Heart Foundation of Australia Chest Pain Every Minute Counts Working G. Patient delay in responding to symptoms of possible heart attack: can we reduce time to care? Med J Aust. 2007;187(5):293-8.
25. Thompson PL, Shilton T. Encouraging early treatment of suspected heart attack: it's OK to call 000. Med J Aust. 2017;206(1):19.
26. Bray JE, Finn J, Cameron P, Smith K, Straney L, Cartledge S, et al. Temporal Trends in Emergency Medical Services and General Practitioner Use for Acute Stroke After Australian Public Education Campaigns. Stroke. 2018;49(12):3078-80.
27. Luepker RV, Raczynski JM, Osganian S, Goldberg RJ, Finnegan JR, Jr., Hedges JR, et al. Effect of a community intervention on patient delay and emergency medical service use in acute coronary heart disease: The Rapid Early Action for Coronary Treatment (REACT) Trial. JAMA. 2000;284(1):60-7.
28. Mooney M, McKee G, Fealy G, O'Brien F, O'Donnell S, Moser D. A review of interventions aimed at reducing pre-hospital delay time in acute coronary syndrome: what has worked and why? European journal of cardiovascular nursing : journal of the Working Group on Cardiovascular Nursing of the European Society of Cardiology. 2012;11(4):445-53.
29. Luepker RV, Murray DM, Jacobs DR, Jr., Mittelmark MB, Bracht N, Carlaw R, et al. Community education for cardiovascular disease prevention: risk factor changes in the Minnesota Heart Health Program. Am J Public Health. 1994;84(9):1383-93.

30. Higginbotham N, Heading G, McElduff P, Dobson A, Heller R. Reducing coronary heart disease in the Australian Coalfields: evaluation of a 10-year community intervention. Soc Sci Med. 1999;48(5):683-92.
31. Dracup K, Bryan-Brown CW. Reducing patient delay in seeking treatment. American journal of critical care : an official publication, American Association of Critical-Care Nurses. 1997;6(6):415-7.
32. National Heart Foundation of Australia. Heart Maps. 2018;https://www.heartfoundation.org.au/for-professionals/heart-maps/australian-heart-maps; accessed 02/2019.
33. The Department of Health and Human Services. Victorian Emergency Minimum Dataset [Available from: https://www2.health.vic.gov.au/hospitals-and-health-services/data-reporting/health-data-standards-systems/data-collections/vemd.
34. The Department of Health and Human Services. Victorian Admitted Episodes Dataset [Available from: https://www2.health.vic.gov.au/hospitals-and-health-services/data-reporting/health-data-standards-systems/data-collections/vaed.
35. Kasza J, Hemming K, Hooper R, Matthews J, Forbes AB, Outcomes ACf, et al. Impact of non-uniform correlation structure on sample size and power in multiple-period cluster randomised trials. Stat Methods Med Res. 2019:962280217734981.
36. Hemming K, Girling A. A menu-driven facility for power and detectable-difference calculations in stepped-wedge cluster-randomized trials. . Stata Journal. 2014;14(2):363-80.
37. Hemming K, Taljaard M, McKenzie JE, Hooper R, Copas A, Thompson JA, et al. Reporting of stepped wedge cluster randomised trials: extension of the CONSORT 2010 statement with explanation and elaboration. BMJ. 2018;363:k1614.
38. Kenward MG, Roger JH. Small sample inference for fixed effects from restricted maximum likelihood. Biometrics. 1997;53(3):983-97.
39. Kasza J, Forbes AB. Inference for the treatment effect in multiple-period cluster randomised trials when random effect correlation structure is misspecified. Stat Methods Med Res. 2018:962280218797151.
40. Hoffmann TC, Glasziou PP, Boutron I, Milne R, Perera R, Moher D, et al. Better reporting of interventions: template for intervention description and replication (TIDieR) checklist and guide. BMJ. 2014;348:g1687.
41. Husereau D, Drummond M, Petrou S, Carswell C, Moher D, Greenberg D, et al. Consolidated Health Economic Evaluation Reporting Standards (CHEERS) statement. BMJ : British Medical Journal. 2013;346:f1049.
42. Lung T, Si L, Hooper R, Di Tanna GL. Health Economic Evaluation Alongside Stepped Wedge Trials: A Methodological Systematic Review. PharmacoEconomics. 2020.
43. Cost-Consequence AnalysisCost-consequence analysis. In: Kirch W, editor. Encyclopedia of Public Health. Dordrecht: Springer Netherlands; 2008. p. 168-.
44. Medical Services Advisory Committee. Technical Guidelines for preparing assessment reports for the Medical Services Advisory Committee - Service Type Investigative. In: Department of Health, editor. Canberra ACT2017.

## APPENDICES

Daily Activity reports HM coordinators

- Date
- Location of presentation
- Number of attendees at presentation
- Materials used
- Presentation type
- Any key barriers to this session?
- Any key facilitators to this session?

HEART MATTERS

# Heart Matters: A stepped wedge cluster randomised trial to test the effectiveness of heart health education in regions at highest-risk

# Statistical Analysis Plan

Version: 1.1 (final)

Date: 22 November 2023


**Authors:**
Jessica Kasza, Rhys Bowden, Janet Bray, on behalf of the Heart Matters Investigators

**Corresponding author:**
Associate Professor Jessica Kasza
School of Public Health and Preventive Medicine
Monash University
Melbourne, Australia
jessica.kasza@monash.edu

# 1 Administrative information

## 1.1 Study identifiers

- Protocol Number: 1.0 (MUHREC: 2020-26296-52553)
- Published protocol reference: Bray J, Nehme Z, Finn J, Kasza J, Clark RA, Stub D, Cadilhac DA, Smith B, Smith K, Cartledge S, Beauchamp A, Dodge N, Walker T, Flemming-Judge E, Chow C, Stewart M, Cox N, van gaal W, Nadurata V, Cameron P. A protocol for the Heart Matters Stepped wedge cluster randomised trial: the effectiveness of heart attack education in regions at highest risk. Resus Plus 2023;15:100431
- ClinicalTrials.gov register Identifier: NCT04995900

## 1.2 Revision history

| Version | Date | Details |
|---|---|---|
| 0.1 (first draft) | 23 May 2023 | First full draft by Jessica Kasza (JK); sent to Janet Bray (JB) for input and addition of non-statistical components |
| 1.0 | December 2023 | Incorporation of comments from JB and JK; input from RB. |
| 1.1 | March 2024 | Incorporation of further comments from JB, JK, RB, Ziad Nehme and Peter Cameron |

## 1.3 Contributors to the statistical analysis plan

### *1.3.1 Roles and responsibilities*

| Name | Affiliation | Role on study | SAP contribution |
|---|---|---|---|
| Associate Professor Jessica Kasza | School of Public Health and Preventive Medicine, Monash University, Melbourne, Australia | Study statistician | Prepared initial draft and revisions |
| Professor Janet Bray | School of Public Health and Preventive Medicine, Monash University, Melbourne, Australia | Principle Investigator | Reviewed all versions; added all non-statistical components |
| Dr Rhys Bowden | School of Public Health and Preventive Medicine, Monash University, Melbourne, Australia | Study statistician | Contributed to finalisation of SAP |
| Dr Ziad Nehme | School of Public Health and Preventive Medicine, Monash University, Melbourne, Australia Ambulance Victoria, Melbourne, Australia | Chief Investigator | Reviewed Version 1.0 |
| Professor Peter Cameron | School of Public Health and Preventive Medicine, Monash University, Melbourne, Australia | Chief Investigator | Reviewed Version 1.0 |

### *1.3.2 Approvals*

The undersigned have reviewed this plan and approve it as final. They find it to be consistent with the requirements of the protocol as it applies to their respective areas. They also find it to be compliant with International Conference on Harmonisation (ICH-E9) principles and in particular, confirm that this analysis plan was developed in a completely blinded manner (i.e. without knowledge of the effect of the intervention[s] being assessed).

| | | | |
|---|---|---|---|
| Signature | Date | Signature | Date |
| **A/Prof J Kasza** | | **Prof J Bray** | |
| Signature | Date | Signature | Date |
| **Dr R Bowden** | | **Co-investigator** | |
| Signature | Date | Signature | Date |
| **Co-investigator** | | **Co-investigator** | |

## Contents

1 Administrative information ... 2

1.1 Study identifiers ... 2

1.2 Revision history ... 2

1.3 Contributors to the statistical analysis plan ... 2

1.3.1 Roles and responsibilities ... 2

1.3.2 Approvals ... 3

2 Introduction ... 7

2.1 Brief background and rationale ... 7

2.2 Study setting and LGA selection ... 7

2.3 Eligibility criteria ... 8

2.4 Interventions ... 8

2.4.1 Educational Intervention ... 8

2.4.2 Control condition ... 8

2.5 Outcomes ... 8

2.5.1 Primary outcome ... 8

2.5.2 Secondary outcomes ... 9

2.6 Randomisation and blinding ... 12

2.7 Research and statistical hypotheses ... 12

2.8 Sample size ... 13

3 Statistical analysis ... 13

3.1 Statistical principles ... 13

3.1.1 Reporting of results ... 13

3.1.2 Level of statistical significance ... 13

3.1.3 Statistical software ... 13

3.2 Analysis populations ... 14

3.3 Impact of COVID-19 ... 14

3.4 Impact of flooding in rural Victoria ... 14

3.5 Changes from the published version of the protocol ... 14

3.6 Baseline descriptions ... 15

3.6.1 Local government area characteristics ... 15

3.6.2 Patient characteristics ... 15

3.7 Analysis of the primary outcome ... 15

3.7.1 Main analysis ........ 16
3.7.2 Different within-cluster correlation structures ........ 16
3.7.3 Adjusted analyses ........ 16
3.7.4 Subgroup analyses ........ 16
3.7.5 Analysis of time-on-treatment ........ 17
3.7.6 Treatment of missing data ........ 17
3.8 Analysis of secondary outcomes ........ 17
3.8.1 Patient delay time (in STEMI and suspected STEMI patients) & prehospital delay time (in STEMI, ACS, and suspected STEMI patients) ........ 17
3.8.2 Binary outcomes: whether patient delay time ≤60mins (in STEMI and suspected STEMI patients); whether prehospital delay time ≤120mins (in STEMI, ACS, and suspected STEMI patients); survival to hospital discharge (in ACS patients); survival to hospital discharge following an out-of-hospital cardiac arrest; acute coronary syndrome emergency department presentations via General Practitioners; calls to EMS for chest pain ........ 17
3.8.3 Number of Heart Health Checks ........ 17
3.8.4 Rates of emergency department presentations for acute coronary syndrome; rates of emergency department presentations for unspecified chest pain; calls to EMS for non-chest pain; rates of out-of-hospital cardiac arrests. ........ 18
3.8.5 Analysis of survey outcomes ........ 18
4 References ........ 19
5 Proposed outputs ........ 21
5.1 Tables ........ 21
5.2 Figures ........ 30

# Tables

Table 1. Number of acute coronary syndrome patients arriving at emergency departments via EMS and total number of acute coronary syndrome patients arriving at emergency departments by LGA and period ......... 21

# Figures

Figure 1. CONSORT diagram ........................................................................................................................ 30

# 2 Introduction

## 2.1 Brief background and rationale

A detailed background is given in the protocol publication.[1] Briefly, reperfusion therapies in acute coronary syndrome (ACS) significantly improve survival rates, particularly when administered promptly.[2, 3] However, patient delay in recognising ACS symptoms and seeking timely medical attention remains a major obstacle to achieving optimal outcomes.[4] Public education campaigns, including mass media initiatives, have shown positive results in increasing ACS awareness and encouraging individuals to promptly contact emergency medical services (EMS) for early treatment.[5, 6] However, sustained exposure to these campaigns is necessary for their long-term effectiveness, and their high cost may not be sustainable.[7] Therefore, an alternative approach is suggested, which involves targeting regions with known high rates of ACS and low EMS use for ACS.

To address this gap in evidence, the Heart Matters trial was established. The trial aims to investigate the impact of community-level education, providing information on ACS risk factors and symptoms, while addressing the known barriers to calling EMS. This trial builds upon previous community-based trials, such as the REACT study,[8] by specifically focusing on regions with high rates and risk of ACS, as well as low EMS use for ACS.

## 2.2 Study setting and LGA selection

The National Heart Foundation of Australia's Victorian Heart Maps were used to identify areas with high rates of ACS. A comprehensive analysis of 32 local government areas (LGAs) in Victoria with high and low rates of acute myocardial infarction (AMI) revealed distinct characteristics in regions with the highest rates. These characteristics included lower education levels, socio-economic disadvantage, higher rates of cardiovascular risk factors, elevated prevalence of other cardiovascular conditions, lower cardiovascular knowledge, and a lower likelihood of using EMS for ACS.[9]

The Heart Matters intervention was implemented in eight of these high-risk LGAs in the Australian state of Victoria. The eight LGAs represent approximately 28% (n=1.8 million) of the state's population. Four LGAs are located in metropolitan Melbourne, including one in the inner city and three on the outskirts. The remaining four LGAs are in rural locations. The final selection of these eight LGAs considered the sample size requirements[10], estimated budget and geographical distance of LGAs to prevent contamination between intervention and control areas.

## 2.3 Eligibility criteria

The educational intervention was available to all adult community members in the selected LGAs. To enhance engagement and delivery, where possible, the Heart Matters Coordinators focused on specific sub-groups and postcodes (priority postcodes) within each LGA that had known low ACS symptom knowledge[7] or low EMS use for ACS.[10]

## 2.4 Interventions

### *2.4.1 Educational Intervention*

The intervention involved delivering the Heart Matters education program to the communities in each LGA. Dedicated HM Coordinators, qualified in relevant fields and familiar with the LGAs, were appointed and received comprehensive training. In the last months of the intervention, when all LGAs had transitioned to the intervention phase, a geotargeted social media campaign (e.g. brief videos on Facebook) was launched in the LGAs.

The educational materials used included Partner resources like the NHFA's Heart Attack Action Plan (available in multiple languages) and program-specific materials such as lived-experience videos and presentation slides. The materials were delivered through various modes (in person or digital, formal or informal) depending on the audience.

### *2.4.2 Control condition*

During the control period, no Heart Matters educational activities took place in the trial LGAs.

## 2.5 Outcomes

Outcomes will be examined for adult (aged 18 years or older) residents of the eight LGAs. All outcomes, except survey data, were collected continuously between December 1$^{st}$ 2022 to March 31$^{st}$ 2023 for the main trial and to March 31$^{st}$ 2024 for long-term follow-up. The survey data was collected at three-time points; December 2021-January 2022 (control), August 2022-September 2022 (half-way), April-May 2023 (completion). This Statistical Analysis Plan provides plans for the analysis of the main trial; the analysis of long-term follow-up data will be covered in a subsequent document.

### *2.5.1 Primary outcome*

- The primary outcome is EMS use for ACS (the proportion of ACS patients that present to a public emergency department [ED] by EMS[10]). The Victorian Department of Health prospectively collects these data in the Victorian Emergency Minimum Dataset (VEMD).

#### *2.5.2 Secondary outcomes*

The following outcomes will be obtained from the Victorian Cardiac Outcomes Registry (VCOR) for ACS patients or ST-elevation myocardial infarction (STEMI) subgroups undergoing reperfusion therapies:

- Patient delay time in STEMI patients (time from symptom onset to decision to seek medical attention; with median delay time to be compared between groups)
- Patient delay within 60 minutes in STEMI patients (the proportion of STEMI patients with patient delay time ≤ 60 minutes to be compared between groups)
- Prehospital delay time in ACS patients (symptom onset to arrival at hospital; with median delay time to be compared between groups)
- Prehospital delay time in STEMI patients (symptom onset to arrival at hospital; with median delay time to be compared between groups)
- Prehospital delay within 120 minutes in ACS patients (the proportion of patients with symptom onset to arrival at hospital ≤120 minutes to be compared between groups)
- Prehospital delay within 120 minutes in STEMI patients (the proportion of patients with symptom onset to arrival at hospital ≤120 minutes to be compared between groups)

The following outcomes will be obtained from the Victorian EMS STEMI Quality Initiative (VASQI) for suspected STEMI patients transported by EMS:

- Patient delay time in suspected STEMI patients (time from symptom onset to decision to seek medical attention [call ambulance]; with median delay time to be compared between groups)
- Patient delay within 60 minutes in suspected STEMI patients (the proportion of suspected STEMI patients with patient delay time ≤ 60 minutes to be compared between groups)
- Prehospital delay time in suspected STEMI patients (symptom onset to arrival at hospital; with median delay time to be compared between groups)
- Prehospital delay within 120 minutes in suspected STEMI patients (the proportion of patients with symptom onset to arrival at hospital ≤120 minutes to be compared between groups)

The following outcomes will be obtained from online survey data collected in residents of the LGAs August 2021-September 2021, August 2022, and April-May 2023

- Awareness of own risk of heart attack (the proportion who self-report having had a heart risk assessment in last two years to be compared between groups)

- Awareness of heart attack/heart disease as the leading cause of death (the proportion who identify heart attack/disease as a leading cause of death in men/women to be compared between groups)
- Awareness of heart disease (cardiovascular) risk factors (the proportion who correctly identify any cardiovascular risk factor as increasing their risk of heart disease to be compared between groups)
- The number of correctly named heart disease (cardiovascular) risk factors (the mean number of correctly named cardiovascular risk factors to be compared between groups)
- Awareness of heart attack signs and symptoms (the proportion aware of any heart attack symptom[7] to be compared between groups)
- The number of correctly named heart attack signs and symptoms (the mean number of correctly named heart attack symptoms[7] to be compared between groups)
- Confidence in knowing how to act (the proportion very confident/confident about what they would do if experiencing a heart attack to be compared between groups)
- EMS use in severe chest pain scenario (the proportion who state they would call EMS to be compared between groups)
- EMS use in chest discomfort scenario (the proportion who state they would call EMS to be compared between groups)

The following will be obtained from Medicare:

- The number of Heart Health Checks (numbers of Medicare claims for Heart Health Checks, items 699/177) by LGA and month. The number of adults eligible for Medicare in each LGA will allow analysis of this as a rate for each LGA in each month.

The following will be obtained from the VEMD:

- Number of ACS emergency department presentations (numbers of ED presentations that have a final ED diagnosis of ACS[10]), by LGA and month. Adult population sizes of LGAs will be obtained from the Australian Bureau of Statistics to allow analysis of this as a rate for each LGA in each month.
- Number of emergency department presentations that are for unspecified chest pain (the number of ED presentations that have a final ED diagnosis of unspecified chest pain[10]), by LGA and month. Adult population sizes of LGAs will be obtained from the Australian Bureau of Statistics to convert this to a rate for each LGA in each month.
- ACS ED presentations via General Practitioners (the proportion of ACS ED presentations referred to ED by General Practitioners to be compared between groups)

The following will be obtained from the Victorian Department of Health's Victorian Admitted Episodes Dataset (VAED):

- Rates of ACS survival (the proportion of ACS patients surviving to hospital discharge to be compared between groups)

The following will be obtained from the Victorian Cardiac Arrest Registry (VACAR):

- Numbers of patients with an out-of-hospital cardiac arrest by LGA and month. Adult population sizes of LGAs will be obtained from the Australian Bureau of Statistics to convert this to a rate for each LGA in each month.[11]
- Survival to hospital discharge following an out-of-hospital cardiac arrest (proportion of patients with out-of-hospital cardiac arrest surviving to hospital discharge out of all patients with out-of-hospital cardiac arrest to be compared between groups[11])

The following will be collected from the Ambulance Victoria computer-aided dispatch (CAD) records:

- Calls to EMS for chest pain (the proportions of chest pain [event type 10] calls to EMS out of all calls to EMS to be compared between groups [12])
- Calls to EMS for non-chest pain (the rate of non-chest pain [non-event type 10] calls to EMS to be compared between groups; adult population sizes of LGAs will be obtained from the Australian Bureau of Statistics to convert this to a rate for each LGA in each month).

**Stepped wedge trial schematic**

The schematic for the Heart Matters stepped wedge trial is displayed below. It has 6 sequences, where 2 local government areas are assigned to each of the first and last sequences of the trial. Periods are each two months long, with transition periods between control and active implementation periods incorporated in each sequence.

Figure 1. Stepped wedge cluster trial design including baseline, transition, and active implementation periods.

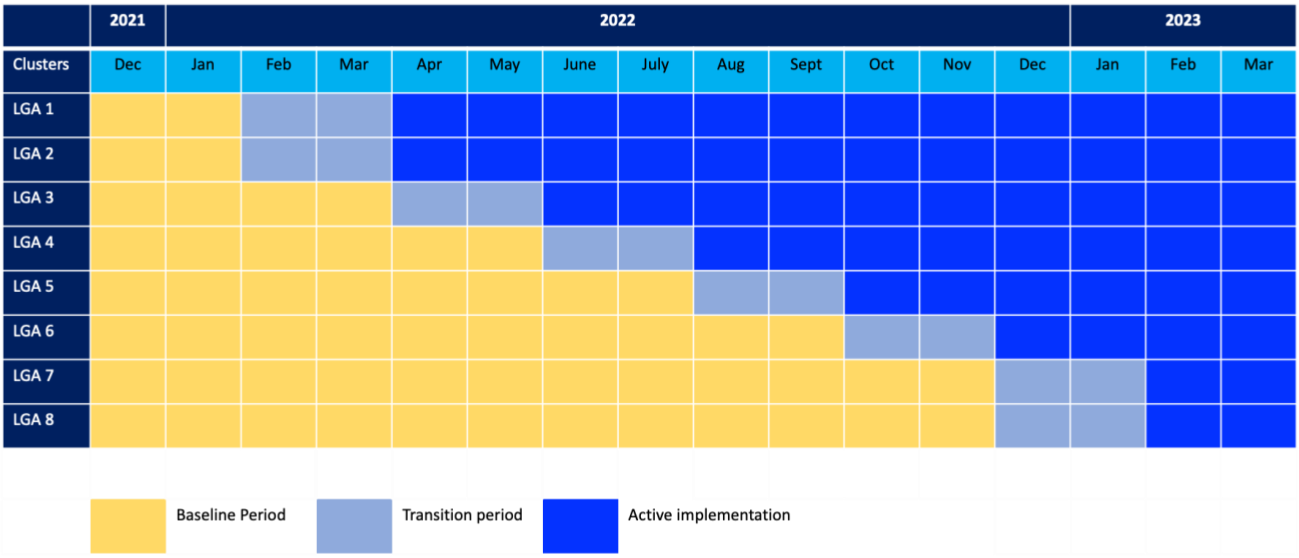


LGA: Local Government Area

## 2.6 Randomisation and blinding

LGAs were randomised to the sequences in the trial schematic shown in Figure 1 with 2 pairs of LGAs in close geographical proximity treated as ``super-clusters’’ and randomised to the same sequence, to better prevent contamination. Over the 16-month study period, it was planned that the eight LGAs would move into the intervention phase at two-month intervals (except for the afore-mentioned pairs, which move to the intervention phase at the same time). For planning purposes, the sequence generation was performed before the study commenced. Randomisation was performed blinded by the Principal Investigator (JB), study statistician (JK) and overseen by an independent investigator. The Principal Investigator randomly allocated each LGA a study number from 1 to 8, which was then sent to the independent investigator. Following this, the study statistician generated the randomisation sequence in Stata using the numbers 1 to 8. This was then linked back to the LGA allocation numbers.

## 2.7 Research and statistical hypotheses

The primary research hypothesis is that the introduction of the Heart Matters education intervention will lead to an increase in the proportion of ACS patients arriving at the hospital via EMS.

The primary statistical hypotheses are as follows:

- **Null hypothesis**: no difference in the proportion of ACS patients that arrive at the emergency department via EMS will be the same before and after the introduction of the Heart Matters education intervention. That is, the risk difference is equal to 1.

- **Alternative hypothesis (2-sided)**: The proportion of ACS patients arriving at the emergency department via EMS will not be the same before and after the introduction of the Heart Matters education intervention. That is, the risk difference is not equal to 1.

### 2.8 Sample size

The stepped wedge design in the schematic (a six-sequence stepped wedge design, with an implementation period and two clusters assigned to the first and last sequences) will give 80% power to detect a change in the proportion of ACS patients in the eight LGAs arriving via EMS by 11%[10, 12] from a baseline of 61% to 72%, with a two-sided significance level of 5%. This detectable difference depends on an average of 40 ACS patients admitted per LGA in each two-month period (i.e. a total of 2240 ACS patients) and on the intra-cluster correlation, estimated to be 0.09 using data from the Victorian Emergency Minimum Dataset (VEMD) (2011-2015). This data indicated that more complex correlation structures, with decaying correlations over time, were unnecessary. The Stepped Wedge Stata Program was used for the sample size calculation,[13] verified using the Shiny CRT app.[14] No adjustment was made for LGA or participant attrition, as health services routinely collect the outcomes.

A sample size calculation was also conducted for two online surveys to determine a change in the level of knowledge of ACS symptoms. An online survey in 2020,[7] showed 25% of adults in the intervention LGAs (20% state-wide) could not name any heart attack symptoms. To detect a change from 25% to 10%, with 80% power and 2-sided significance level, requires a survey of 72 adults in each LGA in months 1-2 and again in months 9-10, assuming an intracluster correlation of 0.1, and a cluster autocorrelation of 0.9.

# 3 Statistical analysis

## 3.1 Statistical principles

#### *3.1.1 Reporting of results*

Results will be reported in accordance with the CONSORT extension for stepped wedge trials.[15]

#### *3.1.2 Level of statistical significance*

All applicable statistical tests will be 2-sided and will be performed using a 5% significance level. No adjustments for multiplicity will be made. Instead, 95% confidence intervals and p-values will be reported.

#### *3.1.3 Statistical software*

Stata v17 or later (StataCorp. Stata Statistical Software: Release 17. College Station, TX: StataCorp LLC), and R version 4.2.2 or later (The R Foundation for Statistical Computing) will be used.

## 3.2 Analysis populations

The primary analyses of primary and secondary outcomes will be conducted under the principle of intention-to-treat. When analysing patient-level outcome data or the survey data, patients will be assumed to have been exposed to the education intervention from the point in time at which their local government area was assigned to commence active implementation of that intervention with data from the transition periods excluded from analysis, regardless of whether the education campaign had commenced, or whether the patient was aware of the education campaign. When analysing data collected at the local government area level the local government area will be assumed to be in the education intervention from the point in time at which that local government area was assigned to actively implement the intervention, with data from the transition periods excluded from analysis.

## 3.3 Impact of COVID-19

The original start day of the Heart Matters intervention was delayed due to the COVID-19 pandemic and associated lockdown measures. However, there were no active Government COVID-19 restrictions in the included LGAs during the trial itself; hence, no analyses are planned to account for the impacts of lockdowns.

## 3.4 Impact of flooding in rural Victoria

Between October 12 and November 30 2022, parts of Victoria were impacted by severe flooding which may have impact on the Heart Matters outcomes. Whether the effect of the Heart Matters intervention on the primary outcome was impacted by flooding will be investigated. All of the trial LGAs were impacted (https://www.disasterassist.gov.au) to varying degrees during this period. A model for the primary outcome will be fit as specified below, with the addition of an LGA-period level term for "impacted by flooding" and an interaction between this term and the intervention term. Estimates and 95% confidence intervals for the intervention effects for the settings of "impacted by flooding" and "not impacted by flooding", together with the p-value of the interaction term, will be reported. The “impacted by flooding” term will be 1 in all LGAs over the October and November period of the study and zero otherwise, and thus will be collinear with the corresponding term for period, which will be dropped from the model.

## 3.5 Changes from the published version of the protocol

The published protocol[1] did not include details on the analysis to assess the impact of flooding. These additions were made prior to any data being made available for analysis. Updates to the planned analysis of outcomes have been made. We have clarified that rates of non-chest pain calls to EMS during control and intervention conditions will be compared.

## 3.6 Baseline descriptions

#### *3.6.1 Local government area characteristics*

A description of the characteristics using data from the Australian Bureau of Statistics will be presented for each LGA. These characteristics include: land size, population numbers, resident demographics (median age, proportion of males, Index of Relative Socio-economic Disadvantage and proportion born overseas).

#### *3.6.2 Patient characteristics*

The number of patients admitted to EDs for ACS will be presented by LGA and study period. The characteristics of patients admitted to EDs for ACS will be summarised as counts and percentages (for discrete characteristics), means and standard deviations (for characteristics with approximately symmetric distributions), or medians and interquartile ranges (for characteristics with skewed distributions). Baseline characteristics will be summarised by intervention group (control vs education intervention) and by LGA and study period. Clustering will not be adjusted for in the presentation of patient characteristics. The following patient characteristics will be summarized and presented:

- Age group
- Sex at birth
- Indigenous status
- Living arrangements
- Country of birth (Australian, other English-speaking, non-English-speaking)
- Preferred language English
- Interpreter required
- ACS sub-type

The characteristics of participants responding to the Heart Watch public survey will be summarised by intervention group, data collection round and local government area. Clustering will not be adjusted for, and the following characteristics will be summarized and presented (in the manner described for patient characteristics above):

- Age group
- Sex
- Indigenous status
- Speaks another language other than English at home
- Highest level of education
- Living arrangements
- Working status
- EMS insurance
- Self-reported medical history

## 3.7 Analysis of the primary outcome

The primary outcome of arrival at hospital via EMS is a binary variable.

### *3.7.1 Main analysis*

Both a relative risk and an absolute risk difference will be estimated.

The absolute risk difference in arrival at hospital via EMS will be estimated by fitting a mixed-effects regression model with a binomial distribution and an identity link function, and random intercepts for local government areas and fixed effects for each period and for the intervention. The Kenward-Roger correction will be applied to adjust for the small number of local government areas.[16] Results will be presented as a risk difference and 95% confidence interval. In addition, an odds ratio will be estimated by fitting a marginal logistic regression model (including terms for period and the intervention) via generalized estimating equations with an independent working correlation structure with the Kauermann and Carroll correction applied to the standard errors, as recommended by Thompson et al.[17]

In the case of non-convergence of the model with an identity link, estimates will be based on the marginal logistic regression model only.

### *3.7.2 Different within-cluster correlation structures*

To assess the impact of the assumption of different within-cluster correlation structures on the estimation of the effect of the education campaign on the primary outcome, the analysis estimating a risk difference in Section 3.7.1 will be repeated assuming a block-exchangeable within-cluster correlation structure, including random effects for cluster and cluster-period.

### *3.7.3 Adjusted analyses*

Since there may be imbalances between the LGAs with respect to various patient-level baseline characteristics, the analyses described in Section 3.7.1 will be repeated including patient characteristics that are known to influence EMS use that are available in the VEMD: age, sex, Australian-born, interpreter required, living arrangements, and ACS sub-type.[10] In the case of non-convergence or collinearity, covariates may be examined for collinearity and sequentially removed from the model until convergence is achieved.

### *3.7.4 Subgroup analyses*

Subgroup analyses to account for the impact of flooding will be conducted as described above.

Subgroup analyses will be conducted separately for each of the following variables: LGA location (metropolitan vs rural), age group (18 to < 45, 45 to <65, 65+), sex, country of birth (Australia vs Other), priority postcodes (priority vs non-priority postcodes), and ACS subtype (acute myocardial infarction vs unstable angina). Terms for these variables will be included in each of the primary analysis models, together with an interaction with

the intervention indicator. Estimates and 95% confidence intervals for the intervention effects for each subgroup, together with the p-value of the interaction term, will be reported.

#### 3.7.5 *Analysis of time-on-treatment*

To investigate whether the effect of the education campaign changed over time, the analysis in Section 3.7.1 will be repeated including separate terms for each duration of time that the intervention was implemented. Separate estimates of the effect of the intervention for each of these durations will be obtained.

#### 3.7.6 *Treatment of missing data*

Given that data are collected via the VEMD, we expect rates of data missingness to be low, and thus complete-case analyses are planned.

### 3.8 Analysis of secondary outcomes

#### 3.8.1 *Patient delay time (in STEMI and suspected STEMI patients) & prehospital delay time (in STEMI, ACS, and suspected STEMI patients)*

Patient delay time and prehospital delay time will be analysed using mixed effects quantile regression models including random intercepts for LGAs and fixed effects for each period and for the intervention. The effect of the intervention will be presented as the difference in median delay times between intervention and baseline periods and associated 95% CIs, where 95% CIs will be based on bootstrapped samples as described in Geraci[18].

#### 3.8.2 *Binary outcomes: whether patient delay time ≤60mins (in STEMI and suspected STEMI patients); whether prehospital delay time ≤120mins (in STEMI, ACS, and suspected STEMI patients); survival to hospital discharge (in ACS patients); survival to hospital discharge following an out-of-hospital cardiac arrest; acute coronary syndrome emergency department presentations via General Practitioners; calls to EMS for chest pain.*

These binary outcomes will be analysed according to the approach outlined in Section on 3.7.1. Analysis of the survival to hospital discharge following an out-of-hospital cardiac arrest outcome will only include participants who had an out-of-hospital cardiac arrest.

#### 3.8.3 *Number of Heart Health Checks*

The difference in rates of heart health checks between groups will be estimated by fitting a marginal Poisson regression model via generalized estimating equations with an offset term for the number of adults eligible for Medicare in each LGA, with an independent working correlation structure and Kauermann and Carroll

standard errors. In addition to the term for intervention condition, terms for study period will also be included in the model.

#### *3.8.4 Rates of emergency department presentations for acute coronary syndrome; rates of emergency department presentations for unspecified chest pain; calls to EMS for non-chest pain; rates of out-of-hospital cardiac arrests.*

The difference in rates of emergency department presentations for ACS and unspecified chest pain, rates of out-of-hospital cardiac arrests and counts of calls between groups will be estimated by fitting a marginal Poisson regression model via generalized estimating equations with an offset term for the population size of each LGA, with an independent working correlation structure and Kauermann and Carroll standard errors. In addition to the term for intervention condition, terms for study period will also be included in the model; such terms will incorporate adjustment for seasonality.

#### *3.8.5 Analysis of survey outcomes*

Awareness of own risk of heart attack, awareness of heart attack/heart disease as the leading cause of death, awareness of heart disease (cardiovascular) risk factors, awareness of heart attack signs and symptoms, confidence in knowing how to act, EMS use in severe chest pain scenario and EMS use in chest discomfort scenario are all binary outcomes and will be analysed in the same manner as the other binary outcomes obtained from other sources. The average number of correctly named heart disease (cardiovascular) risk factors and the number of correctly named heart attack symptoms will be compared between groups by fitting linear mixed models with random intercepts for local government areas and fixed effects for each period and for the intervention. The Kenward-Roger correction will be applied to adjust for the small number of local government areas.

# 4 References

1. Bray JE, Nehme Z, Finn JC, et al. A protocol for the Heart Matters stepped wedge cluster randomised trial: The effectiveness of heart attack education in regions at highest-risk. Resusc Plus. 2023;15:100431.
2. De Luca G, Suryapranata H, Ottervanger JP, Antman EM. Time delay to treatment and mortality in primary angioplasty for acute myocardial infarction: every minute of delay counts. Circulation. 2004;109(10):1223-5.
3. Nadlacki B, Horton D, Hossain S, et al. Long term survival after acute myocardial infarction in Australia and New Zealand, 2009-2015: a population cohort study. Med J Aust. 2021;214(11):519-25.
4. Moser DK, Kimble LP, Alberts MJ, et al. Reducing delay in seeking treatment by patients with acute coronary syndrome and stroke: a scientific statement from the American Heart Association Council on cardiovascular nursing and stroke council. Circulation. 2006;114(2):168-82.
5. Hoschar S, Albarqouni L, Ladwig KH. A systematic review of educational interventions aiming to reduce prehospital delay in patients with acute coronary syndrome. Open Heart. 2020;7(1):e001175.
6. Mooney M, McKee G, Fealy G, et al. A review of interventions aimed at reducing pre-hospital delay time in acute coronary syndrome: what has worked and why? European Journal of Cardiovascular Nursing. 2012;11(4):445-53.
7. Bray JH, S.; Nehme, Z.; Buttery, A.; Stub, D.; Cartledge, S.; Finn, J. Declining public awareness of heart attack warning symptoms in the years following an Australian public awareness campaign: a cross-sectional study. Heart, Lung and Circulation. 2023;inpress. .
8. Luepker RV, Raczynski JM, Osganian S, et al. Effect of a community intervention on patient delay and emergency medical service use in acute coronary heart disease: The Rapid Early Action for Coronary Treatment (REACT) Trial. JAMA. 2000;284(1):60-7.
9. Bray J, Beauchamp R, Clark R, et al. A Comparison of Victorian Regions at Different Risk Levels of Acute Myocardial Infarction - How are They Different? Heart, Lung and Circulation. 2019;28:S357-S8.
10. Eastwood K, Howell S, Nehme Z, et al. Impact of a mass media campaign on presentations and ambulance use for acute coronary syndrome. Open heart. 2021;8(2):e001792.
11. Nehme Z, Andrew E, Bernard S, et al. Impact of a public awareness campaign on out-of-hospital cardiac arrest incidence and mortality rates. Eur Heart J. 2017;38(21):1666-73.
12. Nehme Z, Cameron PA, Akram M, et al. Effect of a mass media campaign on ambulance use for chest pain. Med J Aust. 2017;206(1):30-5.
13. Hemming K, Girling A. A menu-driven facility for power and detectable-difference calculations in stepped-wedge cluster-randomized trials. Stata Journal. 2014;14(2):363-80.
14. Hemming K, Kasza J, Hooper R, et al. A tutorial on sample size calculation for multiple-period cluster randomized parallel, cross-over and stepped-wedge trials using the Shiny CRT Calculator. Int J Epidemiol. 2020;49(3):979-95.
15. Hemming K, Taljaard M, McKenzie JE, et al. Reporting of stepped wedge cluster randomised trials: extension of the CONSORT 2010 statement with explanation and elaboration. BMJ. 2018;363:k1614.
16. Kenward MG, Roger JH. Small sample inference for fixed effects from restricted maximum likelihood. Biometrics. 1997;53(3):983-97.

17. Thompson JA, Hemming K, Forbes A, et al. Comparison of small-sample standard-error corrections for generalised estimating equations in stepped wedge cluster randomised trials with a binary outcome: A simulation study. Stat Methods Med Res. 2021;30(2):425-39.
18. Geraci M. Linear Quantile Mixed Models: The lqmm Package for Laplace Quantile Regression. Journal of Statistical Software. 2014;57(13):1 - 29.

# 5 Proposed outputs

## 5.1 Tables

**Table 1. Number of acute coronary syndrome patients arriving at emergency departments via EMS and total number of acute coronary syndrome patients arriving at emergency departments by LGA and period**

| | Dec/Jan | Feb/Mar | Apr/May | Jun/Jul | Aug/Sep | Oct/Nov | Dec/Jan | Feb/Mar | Total |
|---|---|---|---|---|---|---|---|---|---|
| LGA 1 | n/N (%) | | n/N (%) | n/N (%) | n/N (%) | n/N (%) | n/N (%) | n/N (%) | n/N (%) |
| LGA 2 | n/N (%) | | n/N (%) | n/N (%) | n/N (%) | n/N (%) | n/N (%) | n/N (%) | n/N (%) |
| LGA 3 | n/N (%) | n/N (%) | | n/N (%) | n/N (%) | n/N (%) | n/N (%) | n/N (%) | n/N (%) |
| LGA 4 | n/N (%) | n/N (%) | n/N (%) | | n/N (%) | n/N (%) | n/N (%) | n/N (%) | n/N (%) |
| LGA 5 | n/N (%) | n/N (%) | n/N (%) | n/N (%) | | n/N (%) | n/N (%) | n/N (%) | n/N (%) |
| LGA 6 | n/N (%) | n/N (%) | n/N (%) | n/N (%) | n/N (%) | | n/N (%) | n/N (%) | n/N (%) |
| LGA 7 | n/N (%) | n/N (%) | n/N (%) | n/N (%) | n/N (%) | n/N (%) | | n/N (%) | n/N (%) |
| LGA 8 | n/N (%) | n/N (%) | n/N (%) | n/N (%) | n/N (%) | n/N (%) | | n/N (%) | n/N (%) |
| Total | n/N (%) | n/N (%) | n/N (%) | n/N (%) | n/N (%) | n/N (%) | n/N (%) | n/N (%) | n/N (%) |

n=number of acute coronary syndrome patients arriving at emergency departments via EMS emergency medical services in that LGA in that period; N= total number of acute coronary syndrome patients arriving at emergency departments via emergency medical services EMS in that LGA in that period. Yellow cells = control periods; blue cells = Heart Matters intervention periods; black cells = transition periods. Data from transition periods will not be analysed.

**Table 2. Local government area characteristics**

| LGA name | Area km2 | Residents | Index SED* | Median age | Males (%) | Born overseas (%) |
|---|---|---|---|---|---|---|
| Whittlesea | | | | | | |
| Hume | | | | | | |
| Maribyrnong | | | | | | |
| Greater Bendigo | | | | | | |
| Mildura | | | | | | |
| Wodonga | | | | | | |
| Campaspe | | | | | | |
| Warrnambool | | | | | | |

**Table 3. Baseline characteristics of participants**

| | Control (N = ) | HeartMatters education campaign (N = ) | Total (N = ) |
|---|---|---|---|
| **Age group (years)** | | | |
| 18-<45 | n/N (%) | n/N (%) | n/N (%) |
| 45-<65 | n/N (%) | n/N (%) | n/N (%) |
| 65+ | n/N (%) | n/N (%) | n/N (%) |
| | | | |
| **Sex** | | | |
| Male | n/N (%) | n/N (%) | n/N (%) |
| Female | n/N (%) | n/N (%) | n/N (%) |
| | | | |
| **Indigenous status** | | | |
| Indigenous | n/N (%) | n/N (%) | n/N (%) |
| Non-indigenous | n/N (%) | n/N (%) | n/N (%) |
| | | | |
| **Country of birth** | | | |
| Australia | n/N (%) | n/N (%) | n/N (%) |
| Other English-speaking | n/N (%) | n/N (%) | n/N (%) |
| Non-English speaking | n/N (%) | n/N (%) | n/N (%) |
| | | | |
| **Usual accommodation** | | | |
| Lives with others | n/N (%) | n/N (%) | n/N (%) |
| Lives alone | n/N (%) | n/N (%) | n/N (%) |
| | | | |
| **Preferred language** | | | |
| English | n/N (%) | n/N (%) | n/N (%) |
| Other | n/N (%) | n/N (%) | n/N (%) |
| | | | |
| **Interpreter required** | n/N (%) | n/N (%) | n/N (%) |
| | | | |
| **Acute coronary syndrome sub-type** | | | |
| Acute myocardial infarction | n/N (%) | n/N (%) | n/N (%) |
| Unstable angina | n/N (%) | n/N (%) | n/N (%) |

**Table 4.Characteristics of Heart Watch survey respondents**

| | Control (N = ) | HeartMatters education campaign (N = ) | Total (N = ) |
|---|---|---|---|
| **Age group (years)** | | | |
| 18-<45 | n/N (%) | n/N (%) | n/N (%) |
| 45-<65 | n/N (%) | n/N (%) | n/N (%) |
| 65+ | n/N (%) | n/N (%) | n/N (%) |
| | | | |
| **Sex** | | | |
| Male | n/N (%) | n/N (%) | n/N (%) |
| Female | n/N (%) | n/N (%) | n/N (%) |
| Prefer not to say/other | n/N (%) | n/N (%) | n/N (%) |
| | | | |
| **Aboriginal and/or Torres Strait Islander** | | | |
| **Yes** | n/N (%) | n/N (%) | n/N (%) |
| No | n/N (%) | n/N (%) | n/N (%) |
| Prefer not to say | n/N (%) | n/N (%) | n/N (%) |
| | | | |
| **Self-reported Medical history** | | | |
| Hypertension | n/N (%) | n/N (%) | n/N (%) |
| Hypercholesterolemia | n/N (%) | n/N (%) | n/N (%) |
| Diabetes | n/N (%) | n/N (%) | n/N (%) |
| Heart disease | n/N (%) | n/N (%) | n/N (%) |
| Heart attack | | | |
| | | | |
| **Lives alone** | n/N (%) | n/N (%) | n/N (%) |
| | | | |
| **Highest level of education** | | | |
| <12 year | n/N (%) | n/N (%) | n/N (%) |
| Vocational/TAFE | n/N (%) | n/N (%) | n/N (%) |
| University | n/N (%) | n/N (%) | n/N (%) |
| | | | |
| **Language other than English at home** | | | |
| Yes | n/N (%) | n/N (%) | n/N (%) |
| No | n/N (%) | n/N (%) | n/N (%) |
| | | | |
| **EMS insurance** | n/N (%) | n/N (%) | n/N (%) |

### Table 5. Primary and secondary outcomes

| Outcome | Control n/N (%) | Education campaign n/N(%) | RD/OR/IRR/Difference in Medians (95% CI) | P-value | ICC |
|---|---|---|---|---|---|
| **Primary outcome** | | | | | |
| **Arrival at ED via EMS** | n/N (%) | n/N (%) | | | |
| Risk difference | | | xx.xx (xx.xx to xx.xx) | *0.xxx* | 0.xx |
| Odds ratio | | | *xx.xx (xx.xx to xx.xx)* | | |
| **Secondary outcomes** | | | | | |
| **Patient delay time STEMI** | | | | | |
| As a continuous outcome | Mean (SD); Median [IQR] | Mean (SD); Median [IQR] | *xx.xx (xx.xx to xx.xx)* | *0.xxx* | |
| Binary (<60 mins), Risk difference | n/N (%) | n/N (%) | *xx.xx (xx.xx to xx.xx)* | *0.xxx* | 0.xx |
| Binary (<60 mins), Odds ratio | | | *xx.xx (xx.xx to xx.xx)* | *0.xxx* | |
| **Patient delay time suspected STEMI** | | | | | |
| As a continuous outcome | Mean (SD); Median [IQR] | Mean (SD); Median [IQR] | *xx.xx (xx.xx to xx.xx)* | *0.xxx* | |
| Binary (<60 mins), Risk difference | n/N (%) | n/N (%) | *xx.xx (xx.xx to xx.xx)* | *0.xxx* | 0.xx |
| Binary (<60 mins), Odds ratio | | | *xx.xx (xx.xx to xx.xx)* | *0.xxx* | |
| **Prehospital delay time ACS** | | | | | |
| As a continuous outcome | Mean (SD); Median [IQR] | Mean (SD); Median [IQR] | *xx.xx (xx.xx to xx.xx)* | *0.xxx* | |
| Binary (<120 mins), Risk difference | n/N (%) | n/N (%) | *xx.xx (xx.xx to xx.xx)* | *0.xxx* | 0.xx |
| Binary (<120 mins), Odds ratio | | | *xx.xx (xx.xx to xx.xx)* | *0.xxx* | |
| **Prehospital delay time suspected STEMI** | | | | | |
| As a continuous outcome | Mean (SD); Median [IQR] | Mean (SD); Median [IQR] | *xx.xx (xx.xx to xx.xx)* | *0.xxx* | |
| Binary (<120 mins), Risk difference | n/N (%) | n/N (%) | *xx.xx (xx.xx to xx.xx)* | *0.xxx* | 0.xx |
| Binary (<120 mins), Odds ratio | | | *xx.xx (xx.xx to xx.xx)* | *0.xxx* | |
| **Heart health checks** | n/1000 | n/1000 | *xx.xx (xx.xx to xx.xx)* | *0.xxx* | |

| | | | | | |
|---|---|---|---|---|---|
| **ED presentations for acute coronary syndrome** | n/1000 | n/1000 | *xx.xx (xx.xx to xx.xx)* | *0.xxx* | |
| **ED presentations for unspecified chest pain** | n/1000 | n/1000 | *xx.xx (xx.xx to xx.xx)* | *0.xxx* | |
| **ACS ED presentations via GP** | n/N (%) | n/N (%) | | | |
| Risk difference | | | xx.xx (xx.xx to xx.xx) | *0.xxx* | 0.xx |
| Odds ratio | | | *xx.xx (xx.xx to xx.xx)* | | |
| **Survival to hospital discharge ACS** | n/N (%) | n/N (%) | | | |
| Risk difference | | | *xx.xx (xx.xx to xx.xx)* | *0.xxx* | 0.xx |
| Odds ratio | | | *xx.xx (xx.xx to xx.xx)* | *0.xxx* | |
| **Incidence of OHCA** | n/1000 | n/1000 | *xx.xx (xx.xx to xx.xx)* | *0.xxx* | |
| | | | | | |
| **Survival to hospital discharge following an OHCA** | n/N (%) | n/N (%) | | | |
| Risk difference | | | *xx.xx (xx.xx to xx.xx)* | *0.xxx* | 0.xx |
| Odds ratio | | | *xx.xx (xx.xx to xx.xx)* | *0.xxx* | |
| | | | | | |
| **Calls to EMS for chest pain** | n/N (%) | n/N (%) | | | |
| Risk difference | | | *xx.xx (xx.xx to xx.xx)* | *0.xxx* | 0.xx |
| Odds ratio | | | *xx.xx (xx.xx to xx.xx)* | *0.xxx* | |
| **Calls to EMS for non-chest pain** | n/100 | n/100 | *xx.xx (xx.xx to xx.xx)* | *0.xxx* | |

## Table 6. Survey outcomes

| Outcome | Control n/N (%) | Education campaign n/N(%) | RD/OR/Difference in means (95% CI) | P-value | ICC |
|---|---|---|---|---|---|
| **Awareness of heart attack risk** | n/N (%) | n/N (%) | | | |
| Risk difference | | | xx.xx (xx.xx to xx.xx) | *0.xxx* | 0.xx |
| Odds ratio | | | *xx.xx (xx.xx to xx.xx)* | *0.xxx* | |
| **Awareness of heart attack/disease as the leading cause of death** | n/N (%) | n/N (%) | | | |
| Risk difference | | | *xx.xx (xx.xx to xx.xx)* | *0.xxx* | 0.xx |
| Odds ratio | | | *xx.xx (xx.xx to xx.xx)* | *0.xxx* | |
| **Awareness of heart disease (cardiovascular) risk factors** | n/N (%) | n/N (%) | | | |
| Risk difference | | | *xx.xx (xx.xx to xx.xx)* | *0.xxx* | 0.xx |
| Odds ratio | | | *xx.xx (xx.xx to xx.xx)* | *0.xxx* | |
| **Number of correctly named heart disease (cardiovascular) risk factors** | Mean (SD); Median [IQR] | Mean (SD); Median [IQR] | *xx.xx (xx.xx to xx.xx)* | *0.xxx* | 0.xx |
| **Awareness of heart attack signs and symptoms** | n/N (%) | n/N (%) | | | |
| Risk difference | | | *xx.xx (xx.xx to xx.xx)* | *0.xxx* | 0.xx |
| Odds ratio | | | *xx.xx (xx.xx to xx.xx)* | *0.xxx* | |
| **Number of correctly named heart attack signs and symptoms** | Mean (SD); Median [IQR] | Mean (SD); Median [IQR] | *xx.xx (xx.xx to xx.xx)* | *0.xxx* | 0.xx |
| **Confident in knowing how to act if experiencing a heart attack** | n/N (%) | n/N (%) | | | |
| Risk difference | | | *xx.xx (xx.xx to xx.xx)* | *0.xxx* | 0.xx |
| Odds ratio | | | *xx.xx (xx.xx to xx.xx)* | *0.xxx* | |
| **EMS use in severe chest pain scenario** | n/N (%) | n/N (%) | | | |
| Risk difference | | | *xx.xx (xx.xx to xx.xx)* | *0.xxx* | 0.xx |
| Odds ratio | | | *xx.xx (xx.xx to xx.xx)* | *0.xxx* | |
| **EMS use in severe chest discomfort scenario** | n/N (%) | n/N (%) | | | |
| Risk difference | | | *xx.xx (xx.xx to xx.xx)* | *0.xxx* | 0.xx |
| Odds ratio | | | *xx.xx (xx.xx to xx.xx)* | *0.xxx* | |

## Table 7. Additional analyses of primary outcome, arrival at hospital via EMS.

| Outcome | Control n/N (%) | Education campaign n/N(%) | Risk difference/OR (95% CI) | P | ICC/interaction p-value |
|---|---|---|---|---|---|
| **Arrival at hospital via EMS** | n/N (%) | n/N (%) | | | |
| **Block-exchangeable within-cluster correlation structure** | | | | | |
| Risk difference | | | xx.xx (xx.xx to xx.xx) | *0.xxx* | ICC= 0.xx<br>CAC = 0.xx |
| Odds ratio | | | xx.xx (xx.xx to xx.xx) | *0.xxx* | |
| **Adjusted analysis** | | | | | |
| Risk difference | | | xx.xx (xx.xx to xx.xx) | *0.xxx* | 0.xx |
| Odds ratio | | | xx.xx (xx.xx to xx.xx) | *0.xxx* | |
| **Subgroup analysis by flooding status: risk differences** | | | | | |
| Not impacted by flooding | n/N (%) | n/N (%) | *xx.xx (xx.xx to xx.xx)* | *0.xxx* | Interaction p-value 0.xxx |
| Impacted by flooding | n/N (%) | n/N (%) | *xx.xx (xx.xx to xx.xx)* | *0.xxx* | |
| **Subgroup analysis by flooding status: odds ratios** | | | | | |
| Not impacted by flooding | n/N (%) | n/N (%) | *xx.xx (xx.xx to xx.xx)* | *0.xxx* | Interaction p-value 0.xxx |
| Impacted by flooding | n/N (%) | n/N (%) | *xx.xx (xx.xx to xx.xx)* | *0.xxx* | |

**Table 8. Analysis of primary outcome, assuming different treatment effects for each number of periods on treatment.**

| **Periods on treatment** | Control n/N (%) | Education campaign n/N(%) | Risk difference (95% CI) | P | Odds ratio (95% CI) | P |
|---|---|---|---|---|---|---|
| 1 | n/N (%) | n/N (%) | xx.xx (xx.xx to xx.xx) | *0.xxx* | xx.xx (xx.xx to xx.xx) | *0.xxx* |
| 2 | n/N (%) | n/N (%) | xx.xx (xx.xx to xx.xx) | *0.xxx* | xx.xx (xx.xx to xx.xx) | *0.xxx* |
| 3 | n/N (%) | n/N (%) | *xx.xx (xx.xx to xx.xx)* | *0.xxx* | *xx.xx (xx.xx to xx.xx)* | *0.xxx* |
| 4 | n/N (%) | n/N (%) | xx.xx (xx.xx to xx.xx) | *0.xxx* | xx.xx (xx.xx to xx.xx) | *0.xxx* |
| 5 | n/N (%) | n/N (%) | *xx.xx (xx.xx to xx.xx)* | *0.xxx* | *xx.xx (xx.xx to xx.xx)* | *0.xxx* |
| 6 | n/N (%) | n/N (%) | *xx.xx (xx.xx to xx.xx)* | *0.xxx* | *xx.xx (xx.xx to xx.xx)* | *0.xxx* |

## 5.2 Figures

### Figure 1. CONSORT diagram

### Figure 2. Proportions of primary outcome in each period by LGA

**Figure 3. Results of sub-group analyses: odds ratios. A forest plot of odds ratios and 95% confidence intervals for each subgroup will be presented, with p-value of the interaction term.**

**Figure 4. Results of sub-group analyses: risk differences. A forest plot of risk differences and 95% confidence intervals for each subgroup will be presented, with p-value of the interaction term.**
