# Supplementary material for: Heart Attack Education and EMS Response in High-Risk, Low EMS Usage Areas: A Stepped-Wedge Cluster-Randomized Trial
Source: JAMA Netw Open. 2026 Apr 27;9(4):e268823. doi: 10.1001/jamanetworkopen.2026.8823 (PMC13122394; doi:10.1001/jamanetworkopen.2026.8823)
Supplement: Supplement 2. — eTable 1. Profiles of the eight local government areas in 2021 eTable 2. Engagement strategy and reach overview eTable 3. Coordinators backgrounds eTable 4. Resources used and content eTable 5. In-person session locations, lengths and adaptations eTable 6. Social media campaign eTable 7. The demographics of in-person session attendees based on self-reported data in the evaluation form eFigure 1. Survey results from participants of the in-person sessions eTable 8. Baseline characteristics of ACS patients arriving at emergency departments by emergency medical services (primary outcome) eTable 9. Number of acute coronary syndrome patients arriving at emergency departments via EMS and total number of acute coronary syndrome patients arriving at emergency departments in Heart Matters and Non-Heart Matters LGAS by period eFigure 2. Predictive margins of EMS use proportions at each study period for non-HM and HM LGAs, with 95% confidence intervals eTable 10. Analysis of primary outcome using the outcomes collected during and after the trial, assuming different treatment effects for each number of periods on treatment eTable 11. Number of acute coronary syndrome patients arriving at emergency departments via EMS and total number of acute coronary syndrome patients arriving at emergency departments in Heart Matters LGAs after the trial had ended [file jamanetwopen-e268823-s002.pdf]

Supplemental Online Content

Bray JE, Nehme Z, Finn JC, et al; Heart Matters Investigators. Heat attack education and EMS response in high-risk, low EMS usage areas: a stepped-wedge cluster randomized trial. *JAMA Netw. Open*. 2026;9(4):e268823. doi:10.1001/jamanetworkopen.2026.8823

eTable 1. Profiles of the eight local government areas in 2021

eTable 2. Engagement strategy and reach overview

eTable 3. Coordinators backgrounds

eTable 4. Resources used and content

eTable 5. In-person session locations, lengths and adaptations

eTable 6. Social media campaign

eTable 7. The demographics of in-person session attendees based on self-reported data in the evaluation form

eFigure 1. Survey results from participants of the in-person sessions

eTable 8. Baseline characteristics of ACS patients arriving at emergency departments by emergency medical services (primary outcome)

eTable 9. Number of acute coronary syndrome patients arriving at emergency departments via EMS and total number of acute coronary syndrome patients arriving at emergency departments in Heart Matters and Non-Heart Matters LGAS by period

eFigure 2. Predictive margins of EMS use proportions at each study period for non-HM and HM LGAs, with 95% confidence intervals

eTable 10. Analysis of primary outcome using the outcomes collected during and after the trial, assuming different treatment effects for each number of periods on treatment

eTable 11. Number of acute coronary syndrome patients arriving at emergency departments via EMS and total number of acute coronary syndrome patients arriving at emergency departments in Heart Matters LGAs after the trial had ended

This supplemental material has been provided by the authors to give readers additional information about their work.

© 2026 Bray JE et al. *JAMA Netw Open*.

eTable 1. Profiles of the eight local government areas in 2021.

LGA name	Area km^2	Adult residents	Index SED	Males (%)	Born overseas (%)
**Greater Bendigo (LGA 1)**	2,999	94,484	985	49	10
**Campaspe (LGA 2)**	4,519	29,312	965	49	8
**Wyndham (LGA 3)**	542	212,983	1,006	50	48
**Maribyrnong (LGA 4)**	31	70,707	1,010	50	38
**Warrnambool (LGA 5)**	121	27, 976	995	50	10
**Wodonga (LGA 6)**	432	31,955	973	49	11
**Whittlesea (LGA 7)**	490	172,623	990	50	38
**Hume (LGA 8)**	504	179,927	941	50	40

Abbreviations: km^2, squared kilometers; LGA, local government area; SED, Index of Relative Socio-economic Disadvantage

© 2026 Bray JE et al. *JAMA Netw Open*.

eTable 2. Engagement strategy and reach overview.

Strategy	Details	Delivered
**Local Coordinators**	Recruitment of local Coordinators for each LGA. Coordinators had existing knowledge and often living in their respective areas.	**8** local coordinators
**Grass-roots engagement**	Going to where community already meet and gather, rather than expecting community to come to us. Reaching out to local community groups, services and businesses.	**1,722 groups** approached**, 934 (54%)** responded
**Health services engagement**	Coordinators contacted local GPs to alert them to the project, so they were ready for community members to approach them for Heart Health Checks.	**148** health professional alert to HMs in areas
**Heart Matters education sessions**	Delivering a Heart Matters education session adapted to the audience. From 10 minutes to 1.5 hours and method of delivery from very informal to formal presentation.	**484** sessions delivered ~**10,088** attendees
**Session evaluation**	Evaluation forms at each session including an option for a follow up email with summary of session and related links.	**6,966 (69%)** attendees completed, **2,381 (34%)** requested emailed information
**Information stands**	Hosting information stands at community events, shopping centres and health services.	**25** stands with **2,643** direct engagements
**Resource development**	Education session slides (x2), video series (in language) (x5), Heart attack action plan (in language) (x5), warning signs standing banner (x2), new webpage (x1), social media tiles (x2) and promotional materials (x8).	**17** new community-facing resources
**Resource distribution**	Throughout the intervention, the heart attack action plans (including wallet cards) and Heart Health Check (HHC) brochures were distributed to session participants, information stand visitors, health professionals in the area, and resource drop offs to groups/services to share with their members/customers.	**27,140** Warning signs magnets **12,810** wallet cards **10,900** HHC brochure
**Priority postcode mailout**	In March 2023, Heart Attack Warning Signs Action Plan were delivered to households in the priority postcodes of all metropolitan LGAs and the Wodonga LGA.	**174,110** resident letterboxes
**Email updates**	New updates (e.g. new resource, updated webpage) were emailed to past session participants, session organisers and groups and services who hadn't responded.	**3,362** emails
**Social media**	Social media campaign 31 January – 31 March 2023, the last two months; 24 promotional posts in local Facebook community group pages. Included videos and tiles.	Social media campaign reached over **180,000** residents
**Media**	Local traditional media (print & radio); state and national media in last month.	**17** media in LGAs; **5** state/national media

© 2026 Bray JE et al. *JAMA Netw Open*.

eTable 3. Coordinators backgrounds

- 4 paramedics, 3 female and 1 male
- 1 nurse, female
- 1 physiotherapist, female
- 1 international medical doctor / MPH, female
- 1 first aid instructor / school teacher, female

Coordinators underwent training in use of the Heart Matters resources and content, community engagement, and media.

© 2026 Bray JE et al. *JAMA Netw Open*.

eTable 4. Resources used and content

**PowerPoint presentation** used during inperson sessions.	Full version covered heart disease as a leading cause of death, risk factors, ACS symptoms, action plan, Heart Health Checks, lifestyle changes.<br>Modifications made based on duration of event and audience - ACS symptoms and EMS use highlighted in every session.
**Videos** made for in-person sessions, social media campaign, website. Feature a well known paramedic and patient's experiences (different demographics).	Specifically developed covered ACS symptoms, importance and barriers to EMS use, patient experiences. Translated subtitles in Arabic, Hindi, Vietnamese, Punjabi (currently available at https://www.heartfoundation.org.au/your-heart/heart-attack-warning-signs).
**Warning Signs Action Plans** - magnets and wallet cards	Broader demographic groups added. Available in English, Arabic, Hindi, Vietnamese, Punjabi.
**Social media tiles**	Covered key messages and short videos, links to Heart Matters website
**Heart Health Check (HHC) brochures**	Handouts detailing free Medicare cover annual heart health checks for Australians 45 years or older, what is included (medical history, cholesterol, blood sugar and blood pressure check, lifestyle advice and how to reduce risk)

© 2026 Bray JE et al. *JAMA Netw Open*.

eTable 5. In-person session locations, lengths and adaptations

	LGA		Bendigo	Campaspe	Wyndham	Mariby’ng	Warrn'bool	Wodonga	Whittlesea	Hume	TOTALS
Session adaption elements	**Sessions delivered**		99	126	34	43	35	63	44	40	**484**
	**Priority postcode**	**No.**	27	37	32	13	34	54	12	9	**218**
		**%**	27%	29%	94%	30%	97%	86%	27%	23%	**45%**
	**Diversity group**	**No.**	3	0	11	30	1	3	21	13	**82**
		**%**	3%	0%	32%	70%	3%	5%	48%	33%	**17%**
	**Low English proficiency**	**No.**	0	0	12	30	1	3	20	15	**81**
		**%**	0%	0%	35%	70%	3%	5%	45%	38%	**17%**
	**Translator**	**No.**	3	0	12	16	0	1	10	9	**51**
		**%**	3%	0%	35%	37%	0%	2%	23%	23%	**11%**
	**AED training**	**No.**	80	3	0	0	0	0	0	0	**83**
		**%**	81%	2%	0%	0%	0%	0%	0%	0%	**17%**
	**Delivered with AV**	**No.**	15	1	0	0	0	0	0	0	**16**
		**%**	15%	1%	0%	0%	0%	0%	0%	0%	**3%**
Session length	**9 or less**		0	0	1	0	2	0	1	0	**4**
	**10 - 19 mins**		1	6	1	0	0	9	0	1	**18**
	**20 - 29 mins**		1	2	0	1	6	3	1	3	**17**
	**30 - 39 mins**		7	29	7	33	14	23	10	15	**138**
	**40 - 49 mins**		10	61	17	9	13	25	8	19	**162**
	**50 - 59 mins**		0	0	0	0	0	1	3	0	**4**
	**1 hr +**		80	28	8	0	0	2	21	2	**141**
Not delivered	**Cancelled**		6	12	2	12	3	6	2	4	**47**
	**Postponed**		7	7	2	0	1	1	0	0	**18**
	**Diary conflict**		1	8	1	3	0	0	4	3	**20**

AED, automatic external defibrillator; AV, Ambulance Victoria; LGA, local government area.

© 2026 Bray JE et al. *JAMA Netw Open*.

eTable 6. Social media campaign

Duration: 31 January – 31 March 2023

- Total **video campaign** reached 108,107 with over 259k views of at least 15 sec or more
    - **English video** reached 76,979 with over 180k views of at least 15 sec or more. 196 reactions, 11 comments and 24 shares.
    - **Vietnamese video** reached 15,123 with over 36k views of at least 15 sec or more. 105 reactions, 2 comments and 20 shares.
    - **Arabic video** reached 13,488 with over 15k views of at least 15 sec or more. 97 reactions, 4 comments and 23 shares.
    - **Punjabi video** reached 2,517 with over 16k views of at least 15 sec or more. 24 reactions, 0 comments and 5 shares.
- Total **campaign tiles** reached 181,566 with 3.21% click through rate (n=9,622). 717 reactions, 53 comments and 148 shares
- Facebook campaign **contributed 83.3% of website views** across the campaign period

© 2026 Bray JE et al. *JAMA Netw Open*.

eTable 7. The demographics of in-person session attendees based on self-reported data in the evaluation form (n=6,966 of ~10,088 attendees).

LGA	Age >65 years N (%)	Male gender N (%) N=6,681	Heart attack exp. N (%)	HHC N (%)	COB overseas N (%)	First Nations N (%)	Total
**Greater Bendigo**	1,334 (81)	580 (35)	846 (52)	1,032 (63)	126 (8)	8 (<1)	1641
**Campaspe**	1,097 (75)	598 (41)	751 (51)	836 (57)	130 (9)	9 (1)	1471
**Wyndham**	428 (72)	250 (42)	232 (39)	364 (61)	340 (57)	7 (1)	594
**Maribyrnong**	364 (57)	162 (25)	194 (31)	294 (46)	471 (74)	3 (<1)	636
**Warrnambool**	202 (71)	93 (33)	135 (48)	157 (55)	26 (9)	13 (5)	284
**Wodonga**	690 (77)	341 (38)	471 (52)	572 (63)	113 (13)	20 (2)	901
**Whittlesea**	581 (71)	245 (30)	312 (38)	434 (53)	485 (59)	2 (<1)	824
**Hume**	404 (66)	157 (26)	282 (46)	351 (57)	325 (53)	2 (<1)	615
**Totals**	**5,100 (73)**	**2,426 (35)**	**3223 (46)**	**4,040 (58)**	**2,016 (29)**	**64 (1)**	**6966**

Heart attack exp., has experienced or is close to someone who has experienced a heart attack; HHC, has had a heart health check or something like it; COB overseas, was born in a country other than Australia; First Nations, Identifies as Aboriginal and/or Torres Strait Islander.

© 2026 Bray JE et al. *JAMA Netw Open*.

eFigure 1. Survey results from participants of the in-person sessions (n=6,966* of ~10,088 attendees)

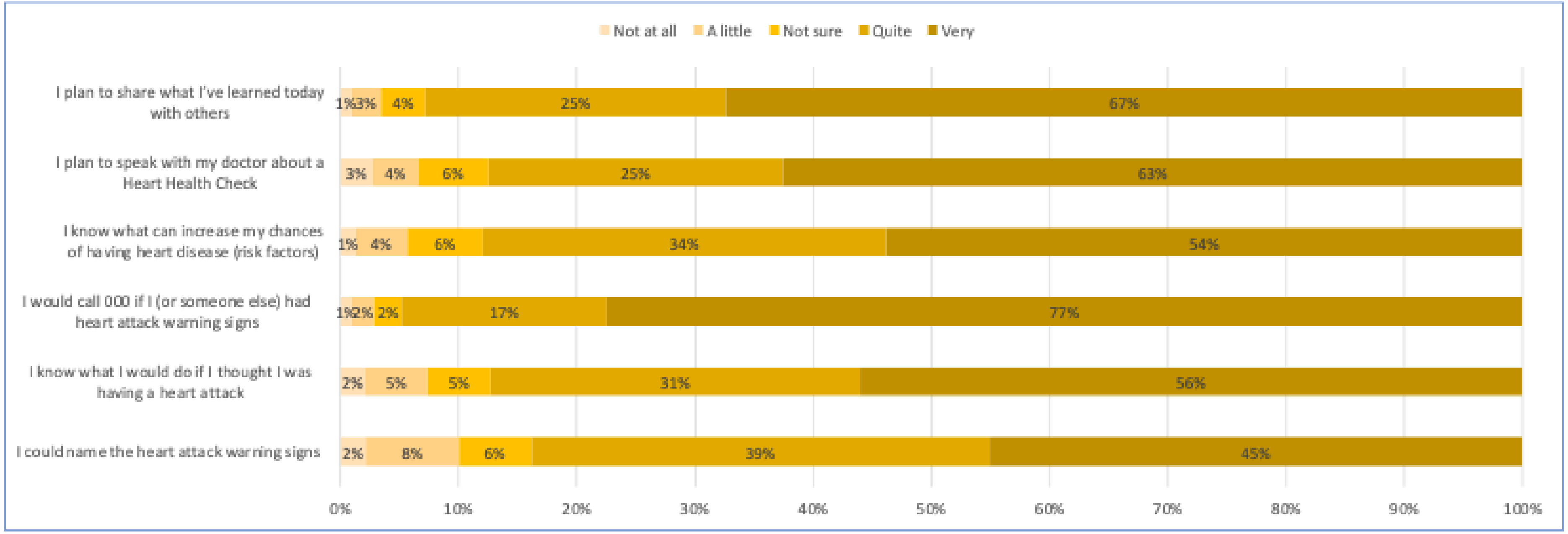

© 2026 Bray JE et al. *JAMA Netw Open*.

eTable 8. Baseline characteristics of ACS patients arriving at emergency departments by emergency medical services (primary outcome).

Characteristic	Control	Education Campaign	Total
N	n=910	n=865	n=1775
**Age group (years)**			
18 to <45	74 (8.1)	71 (8.2)	145 (8.2)
45 to <65	373 (41.0)	333 (38.5)	706 (39.8)
65+	463 (50.9)	461 (53.3)	924 (52.1)
**Sex**			
Female	298 (32.7)	284 (32.8)	582 (32.8)
Male	612 (67.3)	581 (67.2)	1193 (67.2)
**Indigenous Status**			
Indigenous	12 (1.3)	14 (1.6)	26 (1.5)
Not Indigenous	886 (97.4)	836 (96.6)	1722 (97.0)
Unknown	12 (1.3)	15 (1.7)	27 (1.5)
**Country of birth**			
Australia	508 (55.8)	547 (63.2)	1055 (59.4)
Other English speaking	59 (6.5)	56 (6.5)	115 (6.5)
Non-English speaking	331 (36.4)	253 (29.2)	584 (32.9)
Not stated	12 (1.3)	9 (1.0)	21 (1.2)
**Usual accommodation**			
Private Residence - alone	32 (3.5)	58 (6.7)	90 (5.1)
Private Residence - with other(s)	839 (92.2)	770 (89.0)	1609 (90.6)
Residential Aged Care Facility	16 (1.8)	13 (1.5)	29 (1.6)
Other*	6 (0.6)	4 (0.5)	10 (0.6)
Unknown	17 (1.9)	20 (2.3)	37 (2.1)
**Preferred language**			
English	770 (84.6)	777 (89.8)	1547 (87.2)
Other	140 (15.4)	88 (10.2)	228 (12.8)
**Interpreter**			
No	828 (91.0)	794 (91.8)	1622 (91.4)
Yes	82 (9.0)	71 (8.2)	153 (8.6)
**Acute coronary syndrome sub-type**			
Unstable angina	165 (18.1)	102 (11.8)	267 (15.0)
Acute myocardial infarction	745 (81.9)	763 (88.2)	1508 (85.0)

*Other: Boarding/rooming house/hostel/ Community-based supported /Prison/ Remand or Youth Training Centre/ Public Place (Homeless)

© 2026 Bray JE et al. *JAMA Netw Open*.

eTable 9: Number of acute coronary syndrome patients arriving at emergency departments via EMS and total number of acute coronary syndrome patients arriving at emergency departments in Heart Matters and Non-Heart Matters LGAS by period; counts for HM LGAs displayed by intervention status.

	Dec/Jan	Feb/Mar	Apr/May	Jun/Jul	Aug/Sep	Oct/Nov	Dec/Jan	Feb/Mar	Total
**Non HM LGAs**	875/1194 (73.3)	949/1345 (70.6)	982/1438 (68.3)	948/1346 (70.4)	955/1356 (70.4)	962/1366 (70.4)	908/1280 (70.9)	928/1324 (70.1)	7507/10649 (70.5)
**HM LGAs, int = 0**	158/217 (72.8)	126/206 (61.2)	97/142 (68.3)	79/124 (63.7)	79/112 (70.5)	85/109 (78.0)	-	-	624/910 (68.6)
**HM LGAs, int = 1**	-	-	26/42 (61.9)	82/127 (64.6)	72/115 (62.6)	101/158 (63.9)	102/151 (67.5)	165/272 (60.7)	548/865 (63.4)

© 2026 Bray JE et al. *JAMA Netw Open*.

eFigure 2. Predictive margins of EMS use proportions at each study period for non-HM and HM LGAs, with 95% confidence intervals.

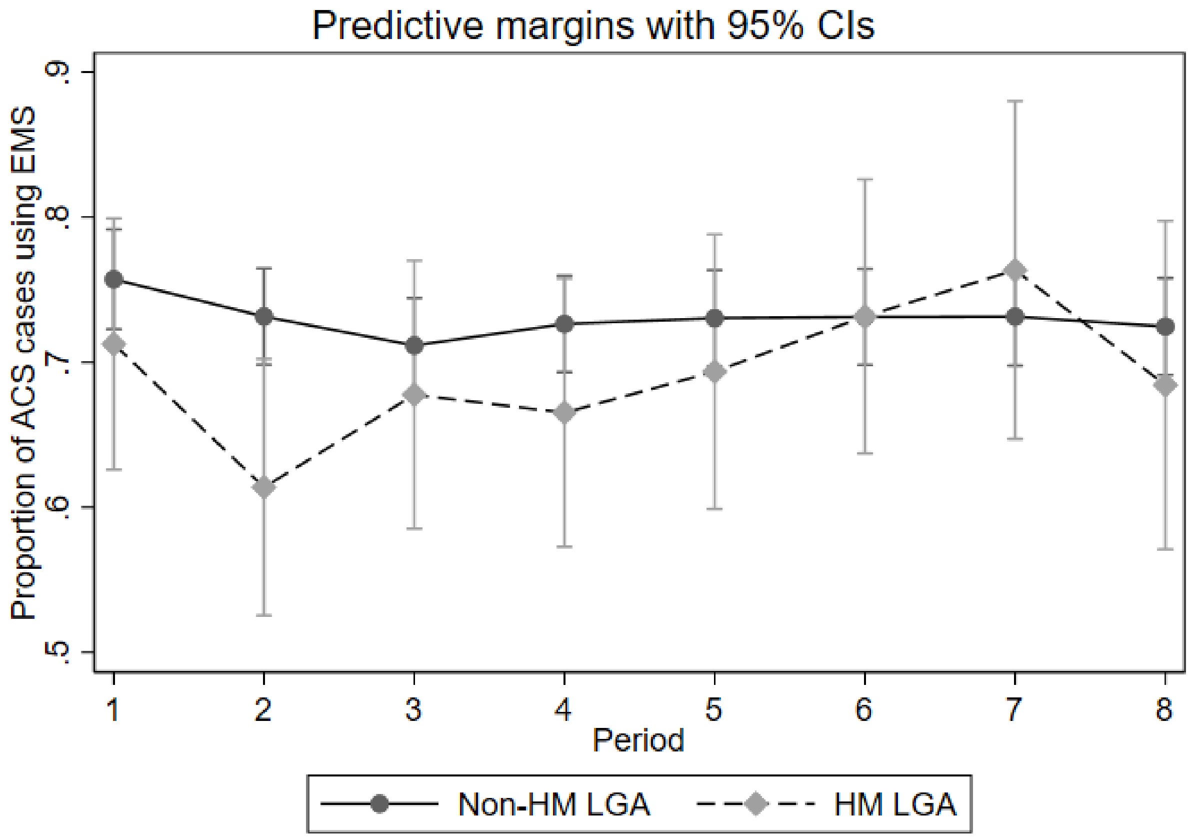

These predictive margins were obtained from a linear mixed model for EMS use including terms for period, a non-HM vs HM indicator, an interaction between these, and an intervention term. Random intercepts were included for LGA.

© 2026 Bray JE et al. *JAMA Netw Open*.

eTable 10: Analysis of primary outcome using the outcomes collected during and after the trial, assuming different treatment effects for each number of periods on treatment

Periods on treatment	Number of events	RD (95% CI)	P-value	OR (95% CI)	P-value
0	624/910 (68.6)				
1	154/262 (58.8)	-7.38 (-16.15,1.39)	0.099	0.67 (0.46,0.96)	0.03
2	169/256 (66.0)	-1.20 (-10.71,8.32)	0.80	0.86 (0.41,1.81)	0.65
3	162/263 (61.6)	-6.64 (-16.89,3.60)	0.20	0.67 (0.38,1.19)	0.14
4	164/255 (64.3)	-3.98 (-15.40,7.43)	0.49	0.76 (0.47,1.22)	0.20
5	170/259 (65.6)	0.41 (-12.04,12.86)	0.95	0.90 (0.51,1.60)	0.68
6 or more	750/1138 (65.9)	3.09 (-10.77,16.94)	0.66	0.96 (0.51,1.79)	0.87

The models fitted here are analogous to that fitted to obtain the time-on-treatment effects in the analysis of the original trial data, with the modification in the number of periods on treatment. Interest here is in the final row: the effect 12 months or more after the introduction of the HM intervention. This indicates that there isn't much difference in EMS use rates 12 months or more after the introduction of the intervention compared to before the intervention. Note: the odds ratios are the R-based results.

© 2026 Bray JE et al. *JAMA Netw Open*.

eTable 11: Number of acute coronary syndrome patients arriving at emergency departments via EMS and total number of acute coronary syndrome patients arriving at emergency departments in Heart Matters LGAs after the trial had ended. Time has been divided into 2-month periods as in the trial.

	Total up to end of the trial	Apr/May	Jun/Jul	Aug/Sep	Oct/Nov	Dec/Jan	Feb/Mar	Total
**LGA 1**	140/197 (71.1)	14/23 (60.9)	18/25 (72.0)	11/15 (73.3)	26/37 (70.3)	19/30 (63.3)	18/29 (62.1)	246/356 (69.1)
**LGA 2**	97/136 (71.3)	12/21 (57.1)	12/17 (70.6)	12/19 (63.2)	16/22 (72.7)	11/17 (64.7)	12/18 (66.7)	172/250 (68.8)
**LGA 3**	285/449 (63.5)	58/72 (80.6)	43/70 (61.4)	47/72 (65.3)	47/73 (64.4)	56/86 (65.1)	40/71 (56.3)	576/893 (64.5)
**LGA 4**	65/109 (59.6)	9/11 (81.8)	11/18 (61.1)	11/14 (78.6)	11/15 (73.3)	6/17 (35.3)	5/8 (62.5)	118/192 (61.5)
**LGA 5**	50/89 (56.2)	6/13 (46.2)	7/11 (63.6)	3/7 (42.9)	10/12 (83.3)	14/21 (66.7)	9/13 (69.2)	99/166 (59.6)
**LGA 6**	75/131 (57.3)	8/19 (42.1)	11/17 (64.7)	6/13 (46.2)	7/16 (43.8)	14/23 (60.9)	13/20 (65.0)	134/239 (56.1)
**LGA 7**	194/284 (68.3)	26/44 (59.1)	36/52 (69.2)	22/35 (62.9)	34/55 (61.8)	23/39 (59.0)	32/41 (78.0)	367/550 (66.7)
**LGA 8**	266/380 (70.0)	38/54 (70.4)	31/52 (59.6)	40/60 (66.7)	35/53 (66.0)	39/50 (78.0)	32/48 (66.7)	481/697 (69.0)
**Total**	1172/1775 (66.0)	171/257 (66.5)	169/262 (64.5)	152/235 (64.7)	186/283 (65.7)	182/283 (64.3)	161/248 (64.9)	2193/3343 (65.6)

© 2026 Bray JE et al. *JAMA Netw Open*.
