# Supplementary material for: Heart Attack Education and EMS Response in High-Risk, Low EMS Usage Areas: A Stepped-Wedge Cluster-Randomized Trial
Source: JAMA Netw Open. 2026 Apr 27;9(4):e268823. doi: 10.1001/jamanetworkopen.2026.8823 (PMC13122394; doi:10.1001/jamanetworkopen.2026.8823)
Supplement: Supplement 3. — Nonauthor Collaborators [file jamanetwopen-e268823-s003.pdf]

Supplemental Online Content: Nonauthor Collaborators
*First name, last name, and suffix (if applicable) are required and will appear in PubMed.

*Group Name(s): Heart Matters Investigators							
***First Name and Middle Initial(s)**	***Last Name**	***Suffix (eg, Jr, III)**	Academic Degrees	Institution	Location (city, state/province, country)	Role or Contribution, eg, chair, principal investigator	Group (if more than 1 Group listed in the byline) and/or Subgroup (eg, Steering Committee)
Karen	Smith		BSc(Hons), Grad Cert Exac BA, Grad Dip Epi & Biostats, PhD	Silverchain	Melbourne, Victoria, Australia	Chief and Site Investigator	Steering Committee
Tony	Walker		BParamedStud, GradDipEmrgHlth (MICA), Master of Educaton	Monash	Melbourne, Victoria, Australia	Associate Investigator	Steering Committee
Adam J	Stormont		Bpharm, MHlthSrvMt, MHlthSrvMt, FCHSM, CHE, GAICD	National Heart Foundation of Australia	Melbourne, VIC, Australia	Steering Committee member	Steering Committee
Amanda K	Buttery		BappSci, MSC, PhD	National Heart Foundation of Australia	Melbourne, Victoria, Australia	Steering Committee member	Steering Committee
Roni	Beauchamp		Grad Dip (Mgmt), MPPMgt	National Heart Foundation of Australia/Australasian Society of Lifestyle Medicine	Melbourne, Victoria, Australia	Steering Committee member	Steering Committee
Erin R	Bowen		EMPA	ANU	Melbourne, Victoria, Australia	Steering Committee member	Steering Committee

*First name, last name, and suffix (if applicable) are required and will appear in PubMed.

***First Name and Middle Initial(s)**	***Last Name**	***Suffix (eg, Jr, III)**	Academic Degrees	Institution	Location (city, state/province, country)	Role or Contribution, eg, chair, principal investigator	Group (if more than 1 Group listed in the byline) and/or Subgroup (eg, Steering Committee)
Roslyn	Cochrane		BHSc(Paramedic), MPH	Ambulance Victoria	Melbourne, Victoria, Australia	LGA Coordinator	Steering Committee
James	Nigro		BHSc(Paramedicine)	Ambulance Victoria	Melbourne, Victoria, Australia	LGA Coordinator	Steering Committee
Donna N	Koik		BEd	National Heart Foundation of Australia	Melbourne, Victoria, Australia	LGA Coordinator	Steering Committee
Alicja T	Najbar-Kaszkiel		Bsci, PhD	National Heart Foundation of Australia	Melbourne, Victoria, Australia	LGA Coordinator	Steering Committee
Nousa	Sooriah		MBBS, MPH	National Heart Foundation of Australia	Melbourne, Victoria, Australia	LGA Coordinator	Steering Committee
Anita	Sterling		BN	Ambulance Victoria	Melbourne, Victoria, Australia	LGA Coordinator	Steering Committee
Georgie	Ockenden		BN	Ambulance Victoria	Melbourne, Victoria, Australia	LGA Coordinator	Steering Committee
Elizabeth	Cerini		Bsci(Physio)	Ambulance Victoria	Melbourne, Victoria, Australia	LGA Coordinator	Steering Committee
Kelly E	Donnelly		BNutDiet	National Heart Foundation of Australia	Melbourne, Victoria, Australia	Study design and funding	Steering Committee
Jesse D	Lewis		Bcomm	National Heart Foundation of Australia	Melbourne, Victoria, Australia	Social media coordinator	Steering Committee
Ararso	Olani		MSc, MPH, PhD (Candidate)	Monash University.	Melbourne, Victoria, Australia	Data collection	Steering Committee
Kelly-Ann	Jolly		MHSci, MPH	National Heart Foundation of Australia	Melbourne, Victoria, Australia	Associate Investigator	Steering Committee
