# Supplementary material for: Heart Attack Education and EMS Response in High-Risk, Low EMS Usage Areas: A Stepped-Wedge Cluster-Randomized Trial
Source: JAMA Netw Open. 2026 Apr 27;9(4):e268823. doi: 10.1001/jamanetworkopen.2026.8823 (PMC13122394; doi:10.1001/jamanetworkopen.2026.8823)
Supplement: Supplement 4. — Data Sharing Statement [file jamanetwopen-e268823-s004.pdf]

Data Sharing Statement

Bray. Heart Attack Education and EMS Response in High-Risk, Low EMS Usage Areas. *JAMA Netw Open*. Published April 27, 2026. doi:10.1001/jamanetworkopen.2026.8823

Data
Additional Information: https://www.clinicaltrials.gov/study/NCT04995900
Data available: No

Additional Information
Explanation for why data not available: Permissions for registry and administrative data do not allow for data sharing.
